# *Tris*-Silanide f-Block Complexes: Insights into Paramagnetic Influence on NMR Chemical Shifts

**DOI:** 10.1021/jacsau.4c00466

**Published:** 2024-07-05

**Authors:** Benjamin L. L. Réant, Fraser J. Mackintosh, Gemma K. Gransbury, Carlo Andrea Mattei, Barak Alnami, Benjamin E. Atkinson, Katherine L. Bonham, Jack Baldwin, Ashley J. Wooles, Iñigo J. Vitorica-Yrezabal, Daniel Lee, Nicholas F. Chilton, Stephen T. Liddle, David P. Mills

**Affiliations:** † Department of Chemistry, The University of Manchester, Oxford Road, Manchester M13 9PL, U.K.; ‡ Department of Chemical Engineering, The University of Manchester, Oxford Road, Manchester M13 9PL, U.K.; § Research School of Chemistry, The Australian National University, Sullivans Creek Road, Canberra 2601, Australian Capital Territory, Australia

**Keywords:** lanthanide, actinide, paramagnetic, NMR spectroscopy, Magic angle spinning, ab initio, DFT calculations

## Abstract

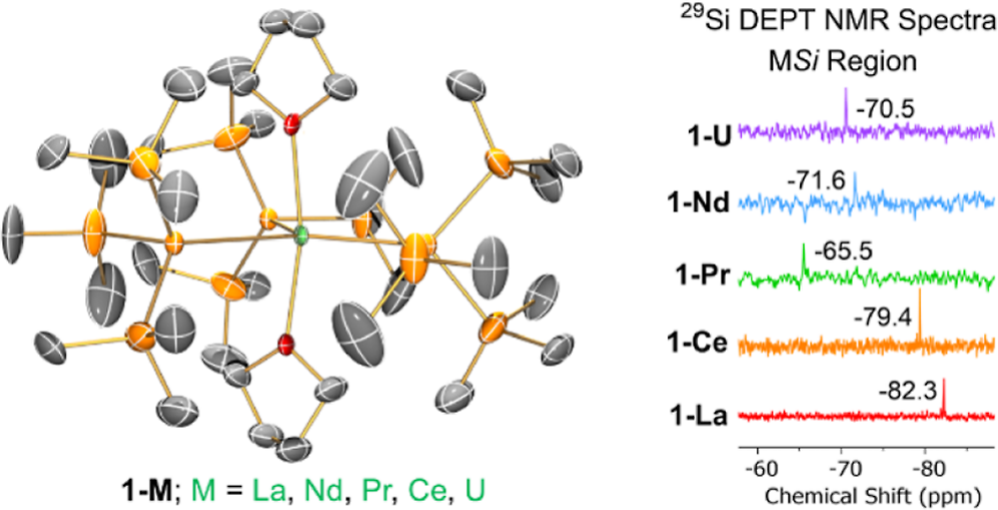

The paramagnetism of f-block ions has been exploited in chiral shift reagents and magnetic resonance imaging, but these applications tend to focus on ^1^H NMR shifts as paramagnetic broadening makes less sensitive nuclei more difficult to study. Here we report a solution and solid-state (ss) ^29^Si NMR study of an isostructural series of locally *D*_3*h*_-symmetric early f-block metal(III) *tris*-hypersilanide complexes, [M{Si(SiMe_3_)_3_}_3_(THF)_2_] (**1-M**; M = La, Ce, Pr, Nd, U); **1-M** were also characterized by single crystal and powder X-ray diffraction, EPR, ATR-IR, and UV–vis–NIR spectroscopies, SQUID magnetometry, and elemental analysis. Only one SiMe_3_ signal was observed in the ^29^Si ssNMR spectra of **1-M**, while two SiMe_3_ signals were seen in solution ^29^Si NMR spectra of **1-La** and **1-Ce**. This is attributed to dynamic averaging of the SiMe_3_ groups in **1-M** in the solid state due to free rotation of the M–Si bonds and dissociation of THF from **1-M** in solution to give the locally *C*_3*v*_-symmetric complexes [M{Si(SiMe_3_)_3_}_3_(THF)_*n*_] (*n* = 0 or 1), which show restricted rotation of M–Si bonds on the NMR time scale. Density functional theory and complete active space self-consistent field spin–orbit calculations were performed on **1-M** and desolvated solution species to model paramagnetic NMR shifts. We find excellent agreement of experimental ^29^Si NMR data for diamagnetic **1-La**, suggesting *n* = 1 in solution and reasonable agreement of calculated paramagnetic shifts of SiMe_3_ groups for **1-M** (M = Pr and Nd); the NMR shifts for metal-bound ^29^Si nuclei could only be reproduced for diamagnetic **1-La**, showing the current limitations of pNMR calculations for larger nuclei.

## 1 Introduction

f-Block metal complexes have been exploited in a wide range of applications that take advantage of their remarkable magnetic and optical behavior.^1^ These applications include chiral shift reagents^2−4^ and PARASHIFT tags for magnetic resonance imaging (MRI),^5−7^ emissive probes for microscopy^8−10^ and biomolecules,^11−16^ and in determining spin–spin coupling in multimetallic systems.^17−19^ Nuclear magnetic resonance (NMR) spectroscopy is widely used to study these parameters and is also a useful technique to assess purity, to investigate exchange coupling and dynamic processes and to extract kinetic and thermodynamic parameters.^20^ As the vast majority of f-block ions are paramagnetic,^1^ the nuclear hyperfine interaction with unpaired electrons in valence f-orbitals give NMR spectra with significant paramagnetic shifts and line broadening.^21−23^ While the ^1^H NMR spectra of paramagnetic f-block complexes can often be assigned and correlated with calculated values to benchmark electronic structures,^21^ less receptive heteroatomic nuclei often give intractable spectra; thus, systematic investigations are rare.^24^

Molecular lanthanide (Ln) and actinide (An) alkyl chemistry is well-developed, with numerous examples of homoleptic Ln(III) *tris*-alkyl complexes.^25,26^ Conversely, f-block silanide chemistry is relatively immature and Ln(III) *tris*-silanide complexes are unknown to date.^27^ More broadly, the applications of f-block silicon chemistry include σ-bond metathesis reactions promoted by Ln silanide complexes,^28,29^ the addition of Ln silicides to low-alloy steels,^30^ and the use of An silicides as high density nuclear fuels.^31−34^ Most structurally authenticated f-block silanide complexes contain the hypersilanide ligand, {Si(SiMe_3_)_3_}^−^, or related derivatives;^27,35^ selected examples include [Ln{Si(SiMe_3_)_3_}_2_(THF)_3_] (Ln = Sm, Eu, Yb),^36^ [Yb(C_5_Me_5_){Si(SiMe_3_)_3_}(THF)_2_],^37^ [Y{Si(SiMe_3_)_3_}(I)_2_(THF)_3_],^38^ [Sc(Cp)_2_{Si(SiMe_3_)_3_}(THF)] (Cp = C_5_H_5_),^39^ [K(18-crown-6)][Ln(Cp)_3_{Si(SiMe_3_)_3_}] (Ln = Ho, Tm; Cp = cyclopentadienyl),^40^ [{K(18-crown-6)}_2_Cp][Ln(Cp)_3_{Si(SiMe_3_)_3_}] (Ln = Ce, Sm, Gd, Tm),^40^ [M(Cp”)_2_{Si(SiMe_3_)_3_}] (M = La, Ce, Nd, U; Cp” = {C_5_H_3_(SiMe_3_)_2_-1,3}),^41^ [Y{C(PPh_2_SiMe_3_)_2_}{Si(SiMe_3_)_3_}(THF)],^42^ [U{N(^t^Bu)(C_6_H_3_Me_2_-3,5)}{Si(SiMe_3_)_3_}],^43^ [U(C_5_H_4_SiMe_3_)_3_{Si(SiMe_3_)_3_}],^44^ and [K(sol1)][U{[Si(SiMe_3_)_2_SiMe_2_]_2_O}(sol2)(I)_2_] (sol1 = (DME)_4_, sol2 = DME; sol1 = 18-crown-6, sol2 = DME or (THF)_2_).^45^ Recently, we used a combination of ^29^Si NMR spectroscopy and density functional theory (DFT) calculations to quantify covalency in diamagnetic *n*f^14^ M(II)–Si bonds for M = Yb and No.^46^ However, these methods are not simply transferable to paramagnetic f-block complexes, where a fully ab initio approach needs to be employed to sufficiently account for open-shell effects in paramagnetic NMR (pNMR) spectra.^47^

Here, we report a solution and solid-state (ss) ^29^Si NMR study of an isostructural series of trigonal bipyramidal early f-block metal(III) *tris*-hypersilanide complexes, [M{Si(SiMe_3_)_3_}_3_(THF)_2_] (**1-M**; M = La, Ce, Pr, Nd, U); **1-M** are also characterized by single crystal and powder X-ray diffraction, EPR, ATR-IR, and UV–vis–NIR spectroscopies, SQUID magnetometry, elemental analysis, and DFT and complete active space self-consistent field spin–orbit (CASSCF-SO) calculations. We find that **1-M** each have only one SiMe_3_ resonance in their ^29^Si ssNMR spectra, which we attribute to dynamic averaging of the SiMe_3_ groups due to free rotation of the M–Si bonds, while dissociation of THF in solution gives the trigonal pyramidal species [M{Si(SiMe_3_)_3_}_3_(THF)_*n*_] (*n* = 0 or 1), which typically exhibit two SiMe_3_ signals in their ^29^Si NMR spectra due to restricted rotation of the M–Si bonds on the NMR time scale. We find excellent agreement of experimental ^29^Si NMR data with DFT-calculated values for diamagnetic **1-La** and reasonable agreement with CASSCF-SO-calculated paramagnetic shifts of SiMe_3_ groups for **1-M** (M = Pr and Nd). The NMR shifts for metal-bound ^29^Si nuclei could only be reproduced for diamagnetic **1-La**, showing the current limitations of predictive pNMR calculations for larger nuclei where significant contact shifts are implicated.

## 2 Results

### 2.1 Synthesis

**Scheme 1:**
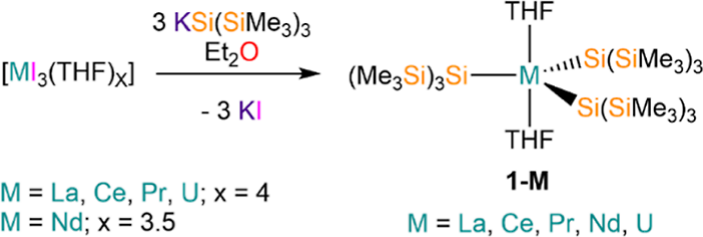
Synthesis of **1-M**

Complexes **1-M** were prepared by salt metathesis reactions between solvated trivalent metal iodide precursors, [MI_3_(THF)_*x*_] (M = La, Ce, Pr, U, *x* = 4; M = Nd, *x* = 3.5), and three equivalents of potassium hypersilanide, [K{Si(SiMe_3_)_3_}], in diethyl ether (Scheme 1). Crystalline samples of **1-M** were obtained in ca. 40% yields (range = 33–51%) following workup and recrystallization from hexane. Microcrystalline **1-M** showed nearly superimposable ATR-IR spectra (see Supporting Information Figures S42–S47), indicating that they have similar solid-state structures. We consistently obtained low carbon values for **1-M** in elemental analyses; we attribute this to incomplete combustion due to silicon carbide formation as this has been postulated for f-block silicon complexes previously, and we note that elemental analysis experiments can be capricious.^48,49^ We therefore interrogated **1-M** by powder XRD and found high phase purity in all cases (see Supporting Information Figures S57–S67 and Table S2).

### 2.2 Structural Characterization

**Figure 1:**
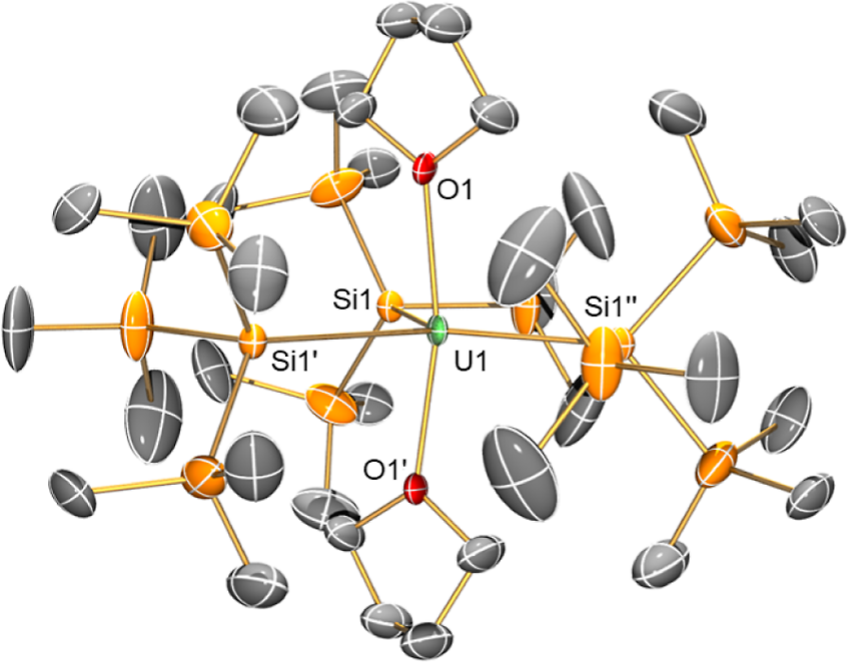
Molecular structure of **1-U** with selective atom labeling collected at 150 K. Displacement ellipsoids set at 30% probability level; hydrogen atoms and disorder components have been removed for clarity.

The molecular structures of **1-M** were confirmed by single crystal XRD (**1-U** is shown in Figure 1 and key metrical parameters for all **1-M** are compiled in Table 1; see Supporting Information Figures S53–S56 for depictions of other **1-M** and Table S3 for additional crystallographic data). Complexes **1-M** exhibit trigonal bipyramidal geometries, with three hypersilanide ligands in the equatorial plane and two axially bound THF molecules. All structures show 120° Si–M–Si angles, with only slight deviations of O–M–Si and O–M–O angles from their respective ideal values (90 and 180°) for local *D*_3*h*_ point symmetry arising from modeling coordinated THF about a 3-fold rotation axis; **1-Pr** shows a larger deviation from *D*_3*h*_ in the solid state as it crystallizes in *P*6_3_, in contrast with the *P*6_3_/*m* space group of other **1-M**. The relative distortions of **1-M** were quantified by calculating τ_5_ parameters, which show the degree of trigonality in a five-coordinate complex (τ_5_ = (β – α)/60; β is the largest and α is the second-largest angle in the coordination sphere); τ_5_ = 1 is ideal trigonal bipyramidal and τ_5_ = 0 is a square based pyramid.^50^ For most of the **1-M** series, τ_5_ = 1, whereas for **1-Pr**, τ_5_ = 0.98.

**Table 1:** M–Si and M–O Bond Distances (Å) and Angles (deg) for **1-M**

	**1-La**	**1-Ce**	**1-Pr**	**1-Nd**	**1-U**
M–Si	3.197(3)	3.172(2)	3.161(3)	3.131(2)	3.114(2)
M–O	2.461(11)	2.389(10)	2.36(2), 2.51(2)	2.407(6)	2.442(6)
Si–M–Si	120	120	120	120	120
O–M–Si	90.03(11)	90.14(7)	87.6(2), 91.3(2)	90.0(4)	90.03(5)
O–M–O	180	180	178.8(2)	180	180

As expected, M–Si and M–O distances decrease across the Ln series with Ln(III) cation size,^51^ with the M–Si distance in 5f^3^**1-U** (3.114(2) Å) shorter than the corresponding distance in 4f^3^**1-Nd** (3.131(2) Å) but longer than the sum of covalent radii of U and Si (2.86 Å).^52^ Complex **1-Pr** contains the first structurally authenticated example of a Pr–Si bond, while the M–Si distances of **1-La**, **1-Ce**, **1-Nd**, and **1-U** are comparable with previously reported hypersilanide complexes of these respective M(III) ions,^27^ e.g., [M(Cp”)_2_{Si(SiMe_3_)_3_}] (M = La, 3.178(2) Å; Ce, 3.153(2) Å; Nd, 3.112(2) Å; U, 3.116(2) Å)^41^ and [{K(18-crown-6)}_2_Cp]][Ce(Cp)_3_{Si(SiMe_3_)_3_}] (3.155(2) Å).^40^ Structurally authenticated U–Si bonds are rare^27^ but have recently been expanded to include the family of U(III) complexes [K(sol1)][U{[Si(SiMe_3_)_2_SiMe_2_]_2_O}(sol2)(I)_2_] (sol1 = (DME)_4_, sol2 = DME; sol1 = 18-crown-6, sol2 = DME or (THF)_2_) (range U–Si: 3.1149(6)–3.1713(14) Å).^45^ In all solid-state structures of **1-M**, three Si–Si bonds are located in the plane defined by the central MSi_3_ motifs, and the remaining six Si–Si bonds are equally arranged above and below this plane in a fan arrangement.

### 2.3 Solution NMR Spectroscopy

Despite the paramagnetism of **1-Ce**, **1-Pr**, **1-Nd**, and **1-U**, we were able to assign solution ^1^H, ^13^C{^1^H} and ^29^Si DEPT90 NMR spectra for all complexes (see Supporting Information Figures S1–S28 for all solution NMR spectra and Table S1 for selected parameters; selected data are compiled in Table 2); these assignments were verified by ^1^H COSY, ^1^H–^13^C HMBC and HSQC, and ^1^H–^29^Si HMBC experiments. Although ^1^H → ^29^Si polarization transfer using the DEPT90 approach is expected to increase the sensitivity of ^29^Si signals, we note that it is not optimal for concomitant ^2^*J* and ^3^*J* scalar coupling; so the metal-bound Si resonances have low intensities. However, during previous investigations of paramagnetic metal complexes using ^29^Si NMR spectroscopy, we have consistently found that signals are most frequently observed in DEPT90 experiments.^41,44,46^ C_6_D_6_ solutions of **1-M** fully decomposed within 10 min to HSi(SiMe_3_)_3_ (assigned by comparison with an authentic sample (δ_Si_ in C_6_D_6_ = −11.6 ppm, SiMe_3_; −115.6 ppm, HSi)^53^); and other products. Resonances associated with a potential impurity, [K{Si(SiMe_3_)_3_}] (δ_Si_ in THF = −4.55 ppm, SiMe_3_; −194.10 ppm, KSi),^54^ were not observed. NMR data were therefore acquired on ca. 9:1 C_6_D_6_: C_4_D_8_O (by volume) solutions of **1-M** (50 mM) that have decomposition *t*_1/2_ > 2 h, allowing reliable ^1^H NMR integrals to be extracted. We found that C_4_D_8_O had to be added before C_6_D_6_ to obtain acceptable NMR spectra, which still contained signals consistent with HSi(SiMe_3_)_3_ and silicone grease impurities; other decomposition products that reproducibly formed were not identified (solutions of **1-Pr** showed the fastest decomposition, see Supporting Information Section 1.6. and Figures S29–S31 for a systematic study).

**Table 2:** Selected ^1^H, ^29^Si DEPT90, and ^13^C{^1^H} NMR Chemical Shifts (ppm) for **1-M**

complex	^1^H NMR			^29^Si DEPT90 NMR		^13^C{^1^H} NMR
	δ_H_(Si(C***H***_3_)_3_) group 1	δ_H_(Si(C***H***_3_)_3_) group 2	Δ(δ_H_)	δ_Si_(M***Si***)	δ_Si_(***Si***Me_3_)	δ_C_(Si(***C***H_3_)_3_)
**1-La**	0.23	0.41	0.18	–82.3	–13.1 (group 1), −5.3 (group 2)	1.39 (group 1), 6.78 (group 2)
**1-Ce**	1.55	–1.43	2.98	–79.4	–11.4 (group 1), −6.4 (group 2)	2.78 (group 1), 6.30 (group 2)
**1-Pr**	8.13	–7.66	15.79	–65.5	–2.9 (group 1)	9.74 (group 1)
**1-Nd**	5.08	–4.63	9.71	–71.6	–6.8 (group 1)	6.51 (group 1)
**1-U**	5.63	–6.84	12.47	–70.5	–6.0 (group 1)	7.05 (group 1)

A sample of **1-La** with an internal standard of 1,3,5-tri*tert*-butylbenzene was dissolved in neat C_4_D_8_O in an effort to reduce sample decomposition prior to collection of NMR spectra and to investigate THF exchange dynamics further. However, this did not provide NMR data that were easier to interpret than those obtained for the 9:1 C_6_D_6_/C_4_D_8_O solution due to the complex exchange behavior, thus we did not extend this study to C_4_D_8_O solutions of paramagnetic **1-M** (see Supporting Information Section S1.7. and Figures S32–S34 for an extended discussion). The ^1^H and ^13^C{^1^H} NMR spectra of **1-La**, **1-Ce**, and **1-U** exhibit only slightly shifted THF resonances compared to free THF; the THF signals in the ^1^H NMR spectra are broadened with unreliable integrals, and THF resonances are not assigned in samples of **1-Pr** and **1-Nd**. We note that a number of signals are observed in the ^1^H and ^13^C{^1^H} NMR spectra of **1-Pr** and **1-Nd** that may arise from THF, but we do not assign them as their identity is uncertain due to sample decomposition and complex exchange equilibria. The lower gyromagnetic ratio of ^13^C nuclei in combination with the generally short relaxation times in these samples precluded the assignment of THF signals via correlation experiments.

Together, these data indicate that THF may dissociate from **1-M** in solution to give [M{Si(SiMe_3_)_3_}_3_(THF)_*n*_] (*n* = 0 or 1), which hereafter we, respectively, refer to as M(THF)_0_ and M(THF)_1_. We posit that the loss of coordinated THF and generation of vacant coordination site(s) is the origin of the relatively facile solution decomposition of **1-M**, as previously found for [Ln(Si^t^Bu_2_R)_2_(THF)_3_] (Ln = Sm, Eu, Yb, R = Me; Ln = Sm, Eu, R = ^*t*^Bu).^46^ The ^1^H NMR spectra of **1-M** each contain two resonances for the SiMe_3_ groups in a 1:2 ratio (herein referred to as group 1 and group 2, respectively), confirming that the time-averaged structures are still 3-fold symmetric; we note that the resonances associated with group 2 are broad (fwhm > 65 Hz) for the most paramagnetic M(III) ions in **1-Nd** and **1-U**.

**Figure 2:**
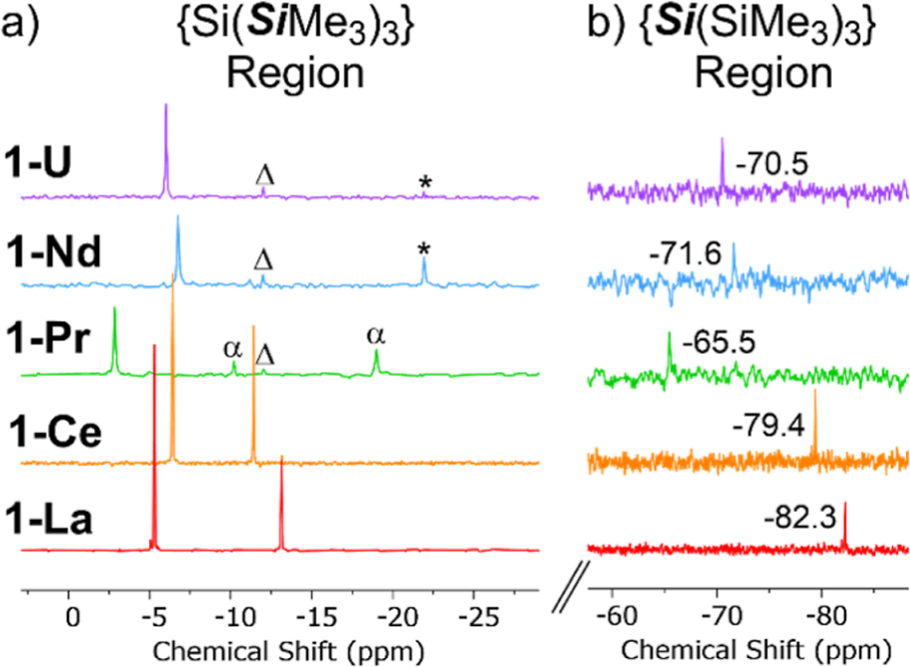
Stacked truncated ^29^Si DEPT90 NMR spectra (79.48 MHz) of **1-M** in 9:1 C_6_D_6_: C_4_D_8_O by volume showing (a) SiMe_3_ resonances between 0 and −25 ppm and (b) MSi resonances between −60 and −85 ppm. α = unidentifiable species, Δ = HSi(***Si***Me_3_)_3_ impurity, * = silicone grease impurity.

While two resonances were seen for SiMe_3_ groups in both the ^13^C{^1^H} and ^29^Si DEPT90 NMR spectra of **1-La** and **1-Ce**, only resonances associated with group 1 were observed in the corresponding spectra of **1-Pr**, **1-Nd**, and **1-U**; this is likely due to the increased anisotropic paramagnetic broadening associated with these ions.^1^ Resonances between δ_Si_ values of −65.5 and −82.3 ppm in the ^29^Si DEPT90 NMR spectra of **1-M** were assigned to the metal-bound silicon atoms (Figure 2); the ^29^Si DEPT NMR spectral windows were +175 to −225 ppm. The ^29^Si shifts observed fall within a relatively narrow range, considering the differences in both paramagnetism and size of the metal ions across the **1-M** series, as the exchange dynamics will vary with metal–ligand distances. The consistent observation of metal-bound silicon resonances for the most paramagnetic **1-M** when some SiMe_3_ resonances were not seen is in accord with increased line-broadening for the latter signals from dynamic THF exchange outweighing the relative effects of paramagnetic broadening. In the ^1^H–^29^Si HMBC NMR spectra of **1-M**, correlations were seen between δ_Si_(MSi) and the δ_H_(Si(CH_3_)_3_) signals corresponding to group 1, but no cross-peaks were observed between δ_Si_(MSi) and the group 2 SiMe_3_ groups. For **1-La** and **1-Ce**, cross-peaks were seen between δ_H_(Si(CH_3_)_3_) and δ_Si_(Si(CH_3_)_3_) for both the group 1 and group 2 SiMe_3_ groups, whereas for **1-Pr**, **1-Nd** and **1-U** correlations were only observed for the group 1 SiMe_3_ groups, as the group 2 SiMe_3_ resonances were not observed by ^13^C{^1^H} and ^29^Si DEPT90 NMR spectroscopy (see above).

### 2.4 Solid-State NMR Spectroscopy

**Figure 3:**
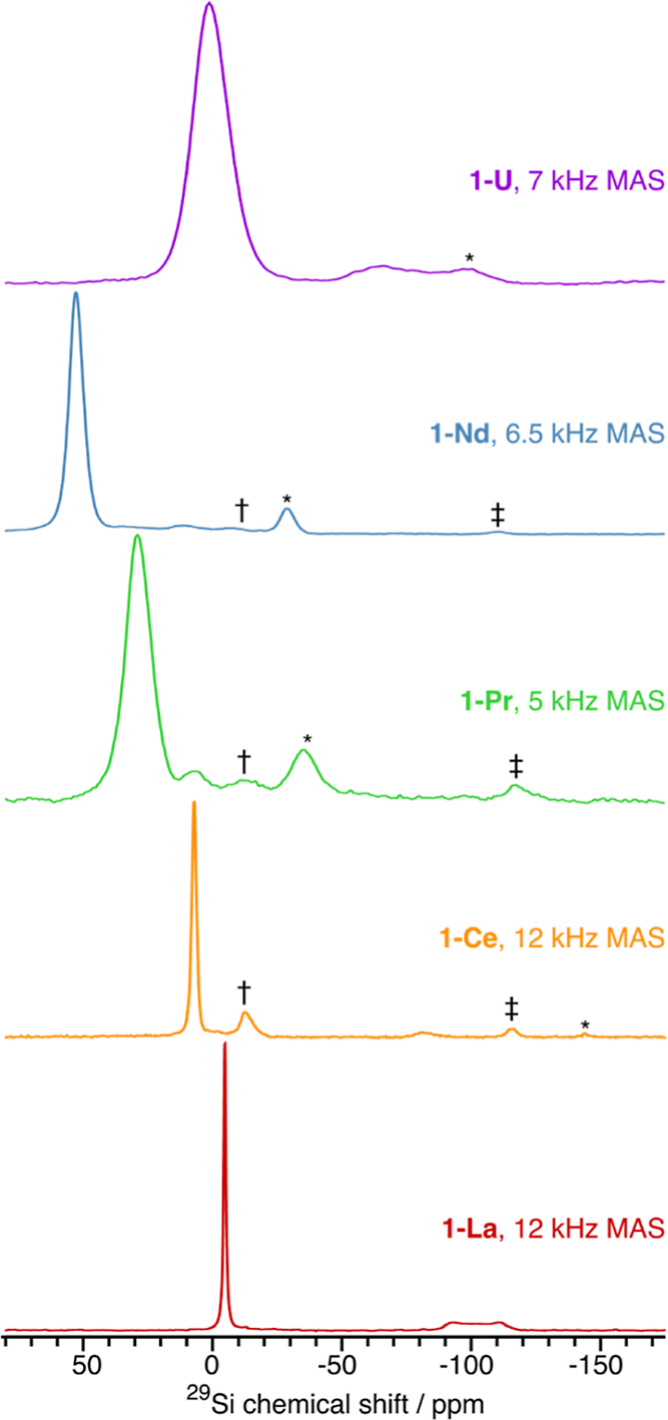
9.4 T solid-state ^29^Si MAS NMR spectra of **1-M** recorded at ambient temperature using the indicated MAS frequencies. The spectra of **1-La**, **1-Ce**, and **1-Pr** were recorded with {^1^H−}^29^Si cross-polarization, while the spectra of **1-Nd** and **1-U** were recorded with direct ^29^Si excitation. Asterisks (*) denote spinning side bands, whereas daggers (†) and double daggers (‡) denote HSi(***Si***Me_3_)_3_ and H***Si***(SiMe_3_)_3_^29^Si resonances from a degradation product, respectively.

As solution NMR spectroscopy indicated that THF readily dissociates from **1-M** in solution to give [M{Si(SiMe_3_)_3_}_3_(THF)_*n*_] (*n* = 0 or 1), we turned to ssNMR spectroscopy to characterize trigonal bipyramidal **1-M** with a known geometry (see Table 3 and Supporting Information Table S2 for selected parameters, Figure 3 for the ^29^Si ssNMR spectra, and Supporting Information Figures S35–S41 for all other spectra). Magic angle spinning (MAS) conditions were employed with spinning frequencies between 5 and 12 kHz; frequencies were selected depending on the sample to give adequate resolution and signal-to-noise ratios, while shifting the spinning side bands from the spectral regions of interest and limiting excessive spinning rates to avoid rotor crashes. The resolution (fwhm) of the ^29^Si ssNMR resonances did not change with MAS frequency [slow (5 kHz) vs moderate (12 kHz) spinning], but the ^1^H ssNMR spectral resolution varied dramatically as expected (see Supporting Information).

**Table 3:** Selected ssNMR Parameters for **1-M**^a^

complex	resonance	^29^Si δ_iso_/ppm	^29^Si δ_11_/ppm	^29^Si δ_22_/ppm	^29^Si δ_33_/ppm	^29^Si resonance fwhm/Hz	^29Si^*T*_2_*/ms	^1H^*T*_1_/ms
**1-La**	M***Si***	–102.6						
	***Si***Me_3_	–4.6	19	–16	–16	29	10.8	710 ± 1
**1-Ce**	***Si***Me_3_	7.2	56	–18	–18	169	1.9	24 ± 1
**1-Pr**	***Si***Me_3_	28.8	122	–18	–18	909	0.4	25 ± 2
**1-Nd**	***Si***Me_3_	52.5	142	35	–20	516	0.6	20 ± 1
**1-U**	***Si***Me_3_	1.1	79	–38	–38	1377	0.2	3.9 ± 0.2

a δ_iso_ = isotropic shift; δ_11_, δ_22_, δ_33_ = principal components of chemical shift tensor; ^1H^T_1_ = ^1^H nuclei spin–lattice relaxation time constant, determined from a saturation-recovery experiment; ^29Si^T_2_* = ^29^Si apparent transverse relaxation time, determined from the peak fwhm. Note that the principal components of the chemical shift tensor are not definitive due to large relative errors in the fitting, particularly for δ_22_ and δ_33_.

For diamagnetic **1-La**, the signal in the ^29^Si MAS NMR spectrum at δ_iso_ = −102.6 ppm is assigned to the metal-bound Si atoms (Figure 3). The line shape of this signal is consistent with an eight-line multiplet owing to the *J* coupling to ^139^La (*I* = 7/2, 99.9% naturally abundant) where unequal lifetimes of the ^139^La Zeeman states, which are on the order of the reciprocal of the *J* coupling (∼290 Hz), cause variable broadening of the multiplet lines (see Supporting Information Figure S35). The isotropic shift of the metal-bound silicon resonance in the ^29^Si MAS NMR spectrum, δ_iso-ss_, is comparable but not identical to the corresponding signal seen in solution, δ_iso-sol_ (Δ_sol-ss_ = 19.5 ppm), which is indicative of a different Si environment. The ^29^Si MAS NMR data of **1-La** can be compared with those recently reported for a series of La(III) Cp silanide anions, though for the majority of these complexes the metal-bound Si atom signals were resolved octets and *J* coupling constants could be extracted straightforwardly: [La(Cp)_3_(SiR_3_)]^−^ (SiR_3_ = Si(H)(C_6_H_2_Me_3_-2,4,6)_2_, δ_iso_: −36.0 ppm, ^1^*J*_LaSi_ = 335 Hz; Si(Me)Ph_2_, δ_iso_: −1.7 ppm, ^1^*J*_LaSi_ = 337 Hz; SiPh_3_, δ_iso_: 7.0 ppm, ^1^*J*_LaSi_ = 318 Hz; Si{Si(H)Ph_2_}Ph_2_, δ_iso_: −21.1 ppm, ^1^*J*_LaSi_ not observed).^55^ Only one signal was observed for the SiMe_3_ groups of **1-La** at δ_iso_ = −4.6 ppm, which is at a similar chemical shift to the group 2 SiMe_3_ groups in the solution ^29^Si DEPT90 NMR spectrum (δ_iso_ = −5.3 ppm). However, given the good intensity and resolution of this spectrum and the clear absence of a second SiMe_3_ signal, we posit that there is dynamic averaging of the SiMe_3_ groups in the solid state at ambient temperature, as seen previously for a Cr(II) borylene complex, [Cr{=BSi(SiMe_3_)_3_}(CO)_5_].^56^

For all paramagnetic **1-M**, we do not observe signals that can be reliably assigned to metal-bound Si atoms, with resonances in the expected region attributed to minor diamagnetic impurities in the sample including HSi(SiMe_3_)_3_^53^ and another signal at −82 ppm that could not be identified. As the relaxation rate that causes paramagnetic broadening is ∝1/*r*^6^ (where *r* is the M–Si distance), we assume that the M*Si* signals are hidden in the baseline due to the degree of magnetic anisotropy of the M(III) ion; this is evidenced by the broad *Si*Me_3_ resonances, e.g., Ce*Si* expected fwhm ∼4 kHz (32 ppm) and U*Si* expected fwhm ∼25 kHz (200 ppm). In solution, these effects are averaged due to molecular tumbling, allowing metal-bound Si resonances to be observed (see above). Only one signal associated with the SiMe_3_ groups is seen in the ^29^Si MAS NMR spectra of all paramagnetic **1-M**, consistent with the spectrum of **1-La** (see above); these signals are paramagnetically shifted from **1-La** to various extents depending on the identity of the M(III) ion (δ_iso_{^29^Si} = 7.2 ppm, **1-Ce**; 28.8 ppm, **1-Pr**; 52.5 ppm, **1-Nd**; 1.1 ppm, **1-U**), with the smaller magnetic anisotropy of Ce(III) resulting in a relatively sharp signal and the larger magnetic anisotropies of Pr(III), Nd(III), and U(III) giving broader resonances. These shifts essentially arise from a large deshielding of the δ_11_ component of the ^29^Si chemical shift tensor (see Table 3), as the δ_22_ and δ_33_ components typically display negligible change (except for **1-Nd** and **1-U**). This infers a large anisotropy of the ^29^Si chemical shielding of the SiMe_3_ moieties and thus asymmetry of the electron distribution along the Si–Si bond, which is influenced by the f-block ion. For **1-Ln**, the magnitude of the deshielding of the δ_11_ component is inversely proportional to the apparent ionic radius (taken from the M–Si bond distance, Table 1) of the f-ion (i.e., δ_11_{^29^*Si*Me_3_} Nd > Pr > Ce > La). However, this is not the case for **1-U**, where a relative shielding of the δ_22_ and δ_33_ components of the ^29^*Si*Me_3_ groups is observed; this could arise from the intrinsic larger covalent effects of 5f vs 4f orbitals, 6d mixing, or a spin–orbit coupling effect.

For the majority of paramagnetic **1-M**, the corresponding ^1^H MAS NMR spectra are relatively uninformative (see Supporting Information Figure S40). However, resonances from metal-bound THF can be observed for **1-Ce** (δ_iso_{^1^H} = 11.3 and 5.9 ppm), which are clearly deshielded compared to those from **1-La** (δ_iso_{^1^H} = 5.2 and 2.6 ppm, see Supporting Information Figure S41), with the CH_2_ protons either side of the O atom showing the largest shift. This arises from the proximity of these atoms to the paramagnetic Ce and results in a large deshielding of the δ_11_ component of the CSA tensor (see Supporting Information Table S2); it appears that the corresponding δ_22_ component becomes more shielded.

### 2.5 UV–Vis–NIR Spectroscopy

**Figure 4:**
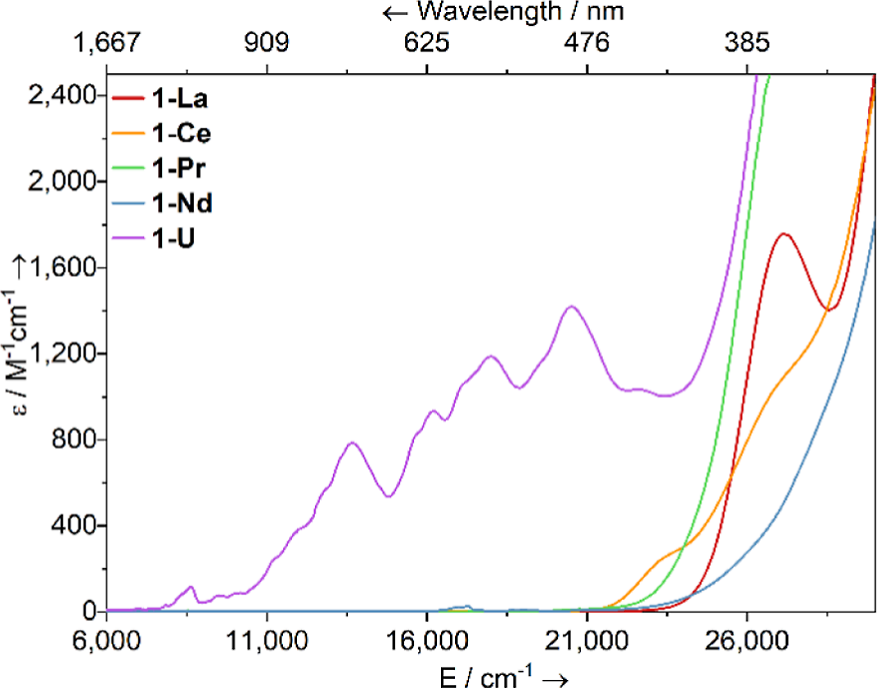
UV–vis–NIR spectrum of **1-M** in THF (2 mM) between 6000 and 30,000 cm^–1^ (1667–333 nm). Legend: M = La (red), Ce (orange), Pr (green), Nd (blue), U (purple).

Complexes **1-M** range in color from pale yellow (**1-La**) to dark green (**1-U**); thus, UV–vis–NIR spectra of **1-M** were recorded at room temperature as 2 mM THF solutions (Figure 4, see Supporting Information Figures S48–S52 for individual spectra); spectra were collected within 15 min of sample preparation at 0 °C; thus, complex solution equilibria are in operation and some sample decomposition will have occurred (see above), so we restrict our interpretation of these data accordingly. All spectra showed intense charge transfer (CT) absorptions tailing in from the UV region to various extents; for **1-La**, a maximum was observed at υ̃_max_ = 27,150 cm^–1^ (ε = 1760 M^–1^ cm^–1^), and for **1-Ce**, two shoulders were seen at υ̃_max_ = 26,750 cm^–1^ (ε = 1010 M^–1^ cm^–1^) and 23,300 cm^–1^ (ε = 240 M^–1^ cm^–1^); the latter of these absorptions can be assigned to a 4f^1^ → 5d^1^ transition, as previously seen for [Ce(Cp″)_2_{Si(SiMe_3_)_3_}].^41^ The spectra of **1-La**, **1-Ce**, and **1-Pr** are otherwise featureless, while **1-Nd** additionally displays a weak set of absorptions in the visible region (υ̃_max_ = 17,250 cm^–1^; ε = 30 M^–1^ cm^–1^) that we assign as f–f transitions arising from the ^4^I_9/2_ → ^4^G_5/2_ states.^57^ In contrast, **1-U** exhibits strong absorptions throughout the visible and NIR regions, which gradually decrease in intensity with increasing wavelength (e.g., υ̃_max_ = 20,500 cm^–1^, ε = 1420 M^–1^ cm^–1^; υ̃_max_ = 8650 cm^–1^, ε = 120 M^–1^ cm^–1^); similarly strong and broad absorptions were recently observed for [K(18-crown-6)][U{[Si(SiMe_3_)_2_SiMe_2_]_2_O}(THF)_2_(I)_2_] (e.g., υ̃_max_ = 17,240 cm^–1^, ε = 1250 M^–1^ cm^–1^; υ̃_max_ = 13,330 cm^–1^, ε = 820 M^–1^ cm^–1^).^45^ While such intense absorptions in U(III) complexes can be assigned as 5f → 6d transitions,^1^ some of these features are likely due to formally Laporte forbidden 5f → 5f transitions,^58−60^ with the “intensity-stealing” due to significant mixing of U 5f orbitals with both 6d orbitals and ligand orbitals.^61−63^

### 2.6 Magnetism

The effective magnetic moment (μ_eff_) and molar magnetic susceptibility (χ_M_*T*) of powdered samples of paramagnetic **1-M** suspended in eicosane were examined by variable-temperature DC SQUID magnetometry and CASSCF-SO (see below) calculations (selected parameters compiled in Table 4, see Supporting Information Figures S68–S70 and Table S5 for all magnetic data). There is good agreement between measured and calculated magnetization values and those expected for free M(III) ions Ce(III) (4f^1 2^F_5/2_), Nd(III) (4f^3 4^I_9/2_), and U(III) (4f^3 4^I_9/2_). However, low magnetic susceptibility values were observed for several different batches of **1-Pr**; at 300 K, the discrepancy between the experimental data presented and the expected value for a Pr(III) ion (4f^2 3^H_4_) and the CASSCF-SO predicted value (see below) is ca. 0.3 cm^3^ mol^–1^ K, which may warrant future investigation.^1^ A gradual decrease in χ*T* with temperature was observed for all **1-M**, due to thermal depopulation of excited crystal field states, with a sharper drop in χ*T* < ca. 30 K attributed to poor thermal equilibration of the sample at lower temperatures. Magnetic saturation (*M*_sat_) was not reached at 2 K for **1-Ce** or **1-Pr** in fields up to 7 T, but this was effectively reached for **1-Nd** and **1-U** under the same conditions, with 2 and 4 K magnetization vs field traces in good agreement with those calculated for **1-Ce**, **1-Nd**, and **1-U**. Both **1-Nd** and **1-U** exhibit similar waist-restricted hysteresis loops at 2 K (see Supporting Information Figure S70).

**Table 4:** Powder Magnetic Moment and Variable-Temperature Molar Susceptibility, μ_eff_ (μ_B_) and χ_M_*T* (cm^3^ mol^–1^ K), of **1-M** Measured by SQUID Magnetometry at 1.8 and 300 K^a^

	SQUID magnetometry				CASSCF-SO calculations			
	1.8 K		300 K		2 K	300 K	free ion at 300 K^1,2^	
complex	μ_eff_	χ_M_*T*	μ_eff_	χ_M_*T*	χ_M_*T*	χ_M_*T*	μ_eff_	χ_M_*T*
**1-Ce**	0.45	0.03	2.33	0.68	0.23	0.70	2.54	0.81
**1-Pr**	1.68	0.35	3.07	1.18	0.56	1.55	3.58	1.60
**1-Nd**	0.82	0.08	3.60	1.62	1.15	1.64	3.62	1.64
**1-U**	1.11	0.16	3.38	1.43	1.16	1.46	3.62	1.64

a χ_M_*T* at 2 and 300 K determined by CASSCF-SO calculations; free ion calculated μ_eff_ and χ_M_*T* values at 300 K.

### 2.7 EPR Spectroscopy

**Figure 5:**
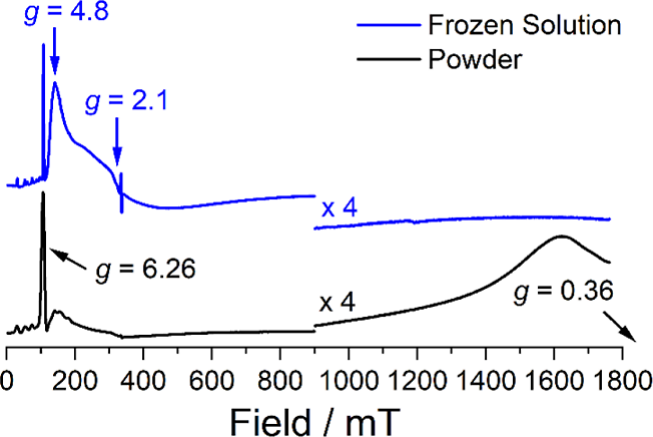
Comparison of X-band EPR spectra of **1-Nd** as a powder at 5 K (black) and as a frozen solution in 2-Me-THF at 7 K (blue).

The electronic structures of the Kramers ion complexes **1-Ce**, **1-Nd**, and **1-U** were probed further by continuous wave X-band (ca. 9.4 GHz) EPR spectroscopy, with spectra modeled using EasySpin.^64^ The easy-axis powder EPR spectrum for **1-Ce** at 7 K (Table 5, see Supporting Information Figure S71) is best modeled as an effective *S* = 1/2 with *g*_1_ = 2.445 and *g*_2_ = 0.786 which are clearly observed, while *g*_3_ ∼ 0.57 is broadened into the baseline. An easy-axis powder EPR spectrum was also observed for **1-Nd** at 5 K, where hyperfine coupling to *I* = 7/2 ^143^Nd (12.2%) and ^145^Nd (8.3%) nuclei could be modeled for the sharp low-field feature (*g*_1_ = 6.26, *A*_1_ = 1860 MHz) and a broad absorption at high field to account for *g*_2_ (0.36), *g*_3_ was not observed (<0.4; Table 5, Figure 5). A frozen solution EPR spectrum of **1-Nd** in 2-Me-THF at 7 K reveals a sharp easy-axis component, consistent with the powder EPR spectrum, in addition to a broad rhombic signal spanning 115–850 mT with a peak centered at *g*_1_ ∼ 4.8, a broad derivative-like feature at *g*_2_ ∼ 2.1, and no resolved *g*_3_ feature (broadened into *g*_2_ or <0.4; Table 5, Figure 5). The broad rhombic signal, which deviates significantly from the solid-state data, could arise from Nd(THF)_*n*_ (*n* = 0 or 1) and/or a range of intermediate geometries; this assertion is supported by the significant change in the *g*-values of the ground Kramers doublet when THF ligands are removed; see below (see Supporting Information Table S23). The powder EPR spectrum of **1-U** is dominated by a broad feature from 100–600 mT that shows two lower-field features at *g* ∼ 6.6 and 5.4 and contains a sharp organic radical signal at *g* = 2.0023 (Table 5, see Supporting Information Figure S72). This spectrum suggests the presence of multiple species from sample decomposition with allowed EPR transitions.

**Table 5:** X-Band EPR Parameters of **1-Ce**, **1-Nd**, and **1-U**

complex	state/temperature	*g*_1_	*g*_2_	*g*_3_	*A*_1_/MHz
**1**-Ce	powder/7 K	2.445	0.786	0.57	
	CASSCF/-	2.378	0.946	0.715	
**1**-Nd	powder/5 K	6.26	0.36		1860
	solution/7 K	∼4.8	∼2.1		
	CASSCF/-	6.032	0.403	0.325	
**1-U**	powder/4 K	6.6, 5.4			
	CASSCF/-	6.097	0.055	0.045	

### 2.8 DFT Calculations

We performed restricted spin orbit relativistic DFT calculations on diamagnetic **1-La** and [La{Si(SiMe_3_)_3_}_3_(THF)_*n*_] (*n* = 0 or 1) using the Amsterdam Density Functional (ADF) suite version 2017 with standard convergence criteria,^65−67^ in order to probe their electronic structures (see Experimental Section for details). The model for **1-La** used the metrical parameters observed by single crystal XRD with geometry-optimized H atom positions, while in the absence of crystallographic metrical data the atomic positions of models of La(THF)_1_ and La(THF)_0_ desolvated analogues were fully geometry-optimized (see Supporting Information Tables S6–S8 for atomic coordinates of geometry-optimized structures). We calculated the δ_Si_ chemical shifts of both the metal-bound Si atoms and the SiMe_3_ groups for **1-La**, La(THF)_1_ and La(THF)_0_ using BP86, PBE0, SAOP and B3LYP functionals, with a range of hybrid density functionals for the latter incorporating between 10 and 50% of the exact exchange energy from Hartree–Fock (HF) theory.^68^ While PBE0 has been used often to calculate NMR chemical shifts,^69−76^ and SAOP often provides good correlation with experimental data despite being less computationally demanding,^46,77,78^ we find that B3LYP with 40% HF exchange energy (B3LYPHF40) gives the best agreement for the complexes investigated herein, noting that in our hands B3LYPHF20-40 calculations have well-reproduced experimental f-element-bound ^13^C, ^15^N, and ^31^P chemical shift parameters.^79−81^ Here, we report mean values of calculated δ_Si_ chemical shifts for metal-bound Si atoms and weighted averages for those within SiMe_3_ groups from all hypersilanide ligands to account for dynamic averaging (Table 6; see Supporting Information Tables S9–S14 for results from other functionals). However, we note that for **1-La**, La(THF)_1_, and La(THF)_0_, there are consistently two sets of predicted SiMe_3_^29^Si NMR signals in a 1:2 ratio separated by between 6 and 10 ppm, in agreement with the solution ^29^Si DEPT90 NMR spectral data (Table 2).

**Table 6:** Experimental and Weighted Averages of Calculated (Using the B3LYPHF40 Functional) Isotropic ^29^Si NMR Chemical Shifts (δ_iso_ in ppm vs SiMe_4_) for **1-La** and [La{Si(SiMe_3_)_3_}_3_(THF)_*n*_] (*n* = 0, La(THF)_0_; *n* = 1, La(THF)_1_)^a^

complex	resonance	^29^Si (exp) δ_iso_/ppm	^29^Si (calcd) δ_iso_/ppm	^29^Si δ_11_/ppm	^29^Si δ_22_/ppm	^29^Si δ_33_/ppm	skew, κ	span, Ω/ppm
**1-La**	M***Si***	–102.6^b^	–102.7	–44.5	–80.4	–183.2	0.48	138.75
	***Si***Me_3_	–4.6^b^	4.0	16.4	5.9	–10.4	0.19	26.78
La(THF)_1_	M***Si***	–82.3^c^	–69.2	–13.6	–44.2	–149.7	0.55	136.07
	***Si***Me_3_	–13.1^c^ (group 1)	2.1^d^	21.0^d^	1.4^d^	–16.2^d^	–0.06^d^	37.17^d^
		–5.3^c^ (group 2)						
La(THF)_0_	M***Si***	–82.3^c^	–43.8	8.5	7.4	–147.3	0.99	155.84
	***Si***Me_3_	–13.1^c^ (group 1)	3.4^d^	20.1^d^	2.3^d^	–12.2^d^	–0.11^d^	32.22^d^
		–5.3^c^ (group 2)						

a δ_11_, δ_22_, δ_33_ = principal components of chemical shift tensor; skew, κ = [3(δ_22_–δ_iso_)]/(δ_11_–δ_33_); span, Ω = δ_11_–δ_33_.

b Solid-state ^29^Si MAS NMR data, corresponding to *n* = 2.

c Solution ^29^Si DEPT90 NMR data, corresponding to *n* = 1.

d Only one value obtained upon averaging.

The computed MDC_q_ charges for La and Si_Si3_ (av.) in **1-La** are 1.14 and −0.47, consistent with their formal +3 and −1 charge states, and reflect net donation of electron density from the ligands to La. The mean Nalewajski-Mrozek La–Si bond indices are 0.56, reflecting the polar-covalent nature of those bonds; for comparison, the corresponding mean La–O_THF_ and Si–Si values are 0.19 and 0.95.

The Frontier Kohn–Sham molecular orbitals (KSMOs) of **1-La** (Figure S73) are as expected, with the HOMO to HOMO–2 reflecting the symmetric and antisymmetric combinations of the three La–Si bonds. However, these KSMOs are rather delocalized, and so we turned to bond localization methods. The Natural Bond Orbital (NBO) method^82^ finds three essentially identical La–Si bonds (Figure S74) of 11 and 89% La and Si character, respectively. The La components are comprised of 25/1/71/3% s/p/d/f character and the Si contributions are 40/60% s/p character; similar orbital breakdowns have previously been calculated for other La silanide complexes.^55^ These data are very similar to the Natural Localized Molecular Orbital (NLMO) representations of **1-La** (Figure S75), which return 11 and 87% La and Si character, respectively. The La and Si components are 35/1/62/2% s/p/d/f and 41/59% s/p. Thus, while the NLMO report increased s-character at the expense of d-contributions for La compared to the NBO interpretation, a fairly consistent bonding picture emerges of La binding to sp hybridized Si atoms utilizing sd^3^ hybrid orbitals.

We examined the La–Si bond topologies of **1-La** using the Quantum Theory of Atoms in Molecules (QTAIM)^83,84^ and found three essentially identical La–Si 3,–1-bond critical points. These exhibit ρ, ∇^2^ρ, H, and ε values of 0.04, 0.02, −0.01, and 0.11. These reflect rather polar and weak La–Si interactions, since covalent bonds tend to have ρ values >0.1 and more negative H terms. The ε term is normally zero, or close to zero, for single and triple bonds, with larger values for double bonds;^85^ the modest ε values here reflect that the silanide ligands each bind to the La center in a slightly skewed manner, which is apparent in the NBO and NLMO visualizations of **1-La** (Figures S74 and S75), and thus their bond ellipticities are not ideal.

### 2.9 Ab Initio Calculations

The electronic structures of paramagnetic **1-M** were investigated by minimal active space CASSCF-SO calculations using OpenMolcas (see Experimental Section and Supporting Information Tables S15–S18 for details).^86^ The ground state for **1-Ce** is dominated by |±3/2⟩ (97%) making it only weakly magnetic with *g*_1_ = 2.38, *g*_2_ = 0.95, *g*_3_ = 0.71, where the largest *g*-value is coincident with the O–Ce–O axis, suggesting that the intermediate |±3/2⟩ *m*_J_ state is stabilized^87,88^ by competition between the hard Lewis base O-donor atoms of THF and the equatorial field imposed by the three softer silanide ligands. The calculated *g*-values are in good agreement with experimental EPR data (Table 5). The CASSCF-SO ground state of **1-Pr** is a mixed 49% |±4⟩ + 49% |∓4⟩ pseudodoublet with a splitting of 6 cm^–1^. The principal magnetic axis is coincident with O–Pr–O, indicating the oblate spheroid |±4⟩ *m*_J_ state is stabilized^87,88^ by O-donor atoms of THF to a greater extent than the silanide ligands. The ground state for **1-Nd** is relatively high purity (90% |±9/2⟩ + 7% |±5/2⟩ + 3% |∓3/2⟩) with *g*_1_ = 6.03 and *g*_2,3_ < 0.4. Similarly for **1-U**, a relatively pure ground doublet was found (89% |±9/2⟩ + 11% |±5/2⟩). The EPR data for **1-U** exhibit overlapping features from decomposition products that obscure the spectrum, although features observed at *g* = 6.6 and 5.4 are comparable to the calculated axial *g*-value (Table 5). The orientation of the magnetic axes for the *m*_J_ = ±9/2 Kramers doublets for **1-Nd** and **1-U** are the same as for **1-Pr** and **1-Ce**.

### 2.10 pNMR Calculations

We calculated the pNMR shifts using two methods (see Experimental Section for details): (i) a point-dipole approximation based on the CASSCF-SO-calculated magnetic susceptibility tensor [pseudocontact shift (PCS) approximation, δ_PCS_^para^];^89^ and (ii) a full sum-over-states expression derived from the derivative of the Helmholtz free energy (van den Heuvel and Soncini’s method δ_vdH-S_^para^).^47^ The latter method is calculated directly based on the CASSCF-SO wave function and implicitly includes all through-bond (i.e., contact) and through-space (i.e., pseudocontact) terms, as well as the relativistic paramagnetic spin–orbit (PSO) terms.^90^ To calculate the experimental paramagnetic shift δ_exp_^para^, we subtracted the diamagnetic NMR signals measured for **1-La** from the respective signals in the paramagnetic compounds. We report calculated δ^para^ values obtained by averaging the calculated shift for all atoms in the same chemical environment, to approximate conformational averaging in the solution phase. We performed calculations using the XRD structure as well as a gas-phase optimized geometry (which retains the same disposition of ligands as in the XRD structure), and model compounds with one or two THF molecules removed (see Experimental Section for details). Upon dissociation of one THF, the central M(Si)_3_ core slightly pyramidalizes and the M–Si bond lengths decrease, which becomes more pronounced when the second THF is displaced (see Supporting Information Tables S19–S21). As suspected, removing THF ligands from **1-M** changes the magnetic anisotropy; this is most clearly observed in the effective *g*-values of the ground Kramers doublets in **1-Ce** and **1-Nd**, which change from easy-axis anisotropy when there are two THF ligands coordinated, to rhombic anisotropy with one THF, and finally to easy-plane anisotropy when both THF ligands are removed (see Supporting Information Tables S22 and S23).

For calculation of δ^para^ in the M(THF)_1_ and M(THF)_0_ structures, there are several models that can be used to account for the solution ^1^H NMR spectra of **1-M** showing a 2:1 ratio for the SiMe_3_ signals. For the M(THF)_1_ structures, one can consider one SiMe_3_ group of each hypersilanide ligand on the same side of the M(Si_M_)_3_ plane as the remaining THF ligand (“cis”) and the other two SiMe_3_ groups on the opposite side (“trans”), as well as the opposite arrangement (two “cis” and one “trans”). For the M(THF)_0_ structures, one can consider one SiMe_3_ group of each hypersilanide ligand closer to the pyramidalized M atom (“proximal”), and the other two further away (“distal”), or vice versa (two “proximal” and one “distal”). In some of our model structures, the assignment is not obvious between the three ligands; thus to address this uncertainty, and to account for dynamic rotation of each of the σ-bonds in the hypersilanide framework, we have used three approaches to account for all the possible scenarios. We define the “plane” as the average plane formed by the four M(Si_M_)_3_ atoms and then determine which nonbound Si atom on each ligand is closest to the plane. In the first approach, these are defined as the group 1 (“in-plane”) SiMe_3_ groups and the other two as the group 2 (“out-of-plane”) groups (see Supporting Information Tables S24–S26). In the second approach, we located the nonbound Si atoms that are farthest from the plane to define group 1, whereas the other two Si atoms that are closer to the plane are defined as group 2 (see Supporting Information Tables S27–S29). In the third approach, we consider the only remaining option for a 2:1 ratio, which is where the SiMe_3_ group closest to the plane is averaged with the one that is farthest from the plane to define group 2, and the intermediate SiMe_3_ group defines group 1 (see Supporting Information Tables S30–S32).

## 3 Discussion

Calculation of the ssNMR data for **1-La** using DFT methods (see Experimental Section) gives mean isotropic ^29^Si NMR chemical shifts of δ_iso_ = −102.7 ppm for the metal-bound Si atoms, in excellent agreement with the experimentally observed ^29^Si MAS NMR shift of −102.6 ppm. By contrast, the weighted average of the calculated SiMe_3_^29^Si shifts for **1-La** (δ_iso_ = 4.0 ppm) do not reproduce the experimental ssNMR value of −4.6 ppm, suggesting that ambient temperature dynamics may need to be considered. Though there is little variance in the mean calculated ^29^Si δ_iso_ values of SiMe_3_ groups between **1-La** and desolvated La(THF)_0_ and La(THF)_1_ (Table 6), the mean of the calculated ^29^Si NMR shifts for the metal-bound Si atoms of **1-La** and desolvated La(THF)_1_ of δ_iso_ = −85.9 ppm is similar to the experimentally obtained solution ^29^Si DEPT NMR value (δ_iso_ = −82.3 ppm); we note that the calculated δ_iso_ = −43.8 ppm for the metal-bound Si atoms of the La(THF)_0_ derivative, which has poor agreement with experiment. Furthermore, the difference in δ_Si_ values of Group 1 and Group 2 SiMe_3_ groups obtained from a 9:1 C_6_D_6_/C_4_D_8_O solution of **1-La** at room temperature (Δ*Si*Me_3_ = 7.8 ppm) is close to the mean (Δ*Si*Me_3_ = 8.1 ppm) of equivalent differences calculated for **1-La** (Δ*Si*Me_3_ = 9.9 ppm; δ_iso_ = −2.6 and 7.3 ppm) and its M(THF)_1_ analogue (Δ*Si*Me_3_ = 6.3 ppm; δ_iso_ = −2.1 and 4.2 ppm). These data are in accord with **1-La** forming a dynamic equilibrium with the desolvated La(THF)_1_ form and deuterated analogues in the solution NMR experiments. We therefore posit that the presence of two signals for SiMe_3_ groups in solutions of **1-M** is due to restricted rotation of the M–Si bonds, likely due to the desolvated species showing stronger interactions of coordinatively unsaturated M(III) ions with hypersilanide ligands and the resultant metal coordination spheres being more congested, consistent with the optimized gas-phase geometries.

**Figure 6:**
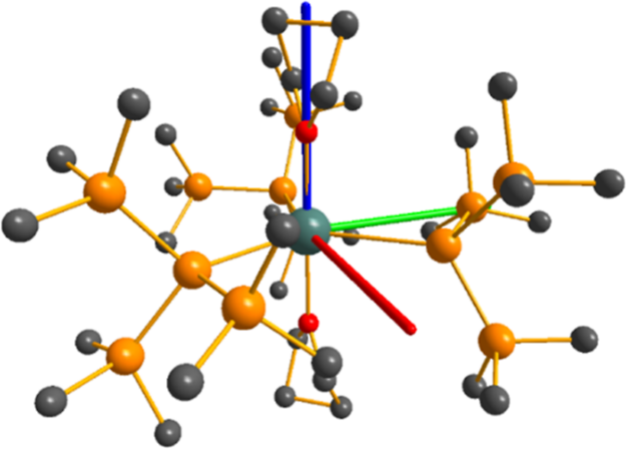
CASSCF-SO-calculated magnetic axes for **1-Ce** (blue: *g*_1_, most magnetic; green: *g*_2_, intermediate; red: *g*_3_, least magnetic) for complexes. Metal, silicon, carbon, and hydrogen atoms shown as metallic green, orange, gray, and light gray, respectively.

The solid-state magnetic and EPR data of paramagnetic **1-M** are consistent with the CASSCF-SO calculations performed on the solid-state XRD structures, which show easy-axis magnetic ground states in all cases, seemingly dictated by the axial THF ligands (Figure 6). As with the solution NMR data of diamagnetic **1-La** (see above), the solution EPR and NMR data of paramagnetic **1-M** indicate that THF may be lost in solution, and this scenario can be probed further by analysis of the solution phase pNMR shifts. Although solvent effects were included for the DFT calculations of **1-La** by introducing a benzene continuum, they were not for the ab initio calculations of paramagnetic **1-M**. The local structure was used for pNMR calculations, as this has the largest effect on the magnetic anisotropy and hence pNMR shifts; the dynamic THF equilibrium will have a far greater influence than “outer sphere” solvent effects. We focus here on pNMR shifts of **1-Ce**, **1-Pr**, and **1-Nd**, which have simpler electronic structures than **1-U** (Figure 4).^91^

Comparing the two CASSCF-SO-based theoretical methods for calculation of pNMR shifts to the solution data of paramagnetic **1-Ln** (see Supporting Information Tables S24–S32), we find that the δ_PCS_^para^ and δ_vdH-S_^para^ values are in close agreement for all ^1^H pNMR signals, which is to be expected as these nuclei are far away from the spin density of the buried 4f orbitals. However, the PCS approximation will be less accurate for nuclei closer to the metal ion where delocalization of spin-density is nonzero, and indeed, this is what we observe for the ^29^Si pNMR shifts, even for the noncoordinated ^29^Si nuclei. The ^29^Si NMR resonances of the metal-bound silicon atoms were not well-reproduced as they are hugely sensitive to contact shift. The contact shift contribution to chemical shift is nontrivial, arising from core spin polarization and dynamic electron correlation, intersecting with relativistic core effects, thus we cannot rationalize any trends.

Comparing the experimental solution phase ^1^H δ_exp_^para^ to the calculated δ^para^ results using either XRD or gas-phase optimized structures, we find that the group 1 SiMe_3_ shifts have the incorrect sign and the group 2 SiMe_3_ shifts are under-predicted in magnitude by several ppm (see Supporting Information Tables S24–S26). As the solution pNMR data show local C_3*v*_ point symmetry of the complex (borne out as a 1:2 ratio of the SiMe_3_^1^H signals, viz., groups 1 and 2, respectively, with restricted rotation around the M–Si bonds due to steric clashing in the presence of shorter M–Si bonds), the molecules must possess axially symmetric magnetic anisotropy. Furthermore, owing to the good applicability of the PCS approximation for the ^1^H nuclei in this case (i.e., the difference between δ_PCS_^para^ and δ_vdH-S_^para^ is small), we can therefore write .^89^ Because the δ_exp_^para^ are positive for the group 1 and negative for the group 2 ^1^H nuclei, and χ_*z*_ – χ̅ is a constant common to both sets of ^1^H environments, then the structural part  must have a different sign for each group of protons. Assuming first that the disposition of the SiMe_3_ groups does not change significantly in solution, the group 1 “in-plane” protons would on average be closer to θ = 90°, while the group 2 “out-of-plane” protons would be closer to θ = 0° or θ = 180°. This indicates that the structural part of the term for group 1 is < 0, while for group 2, it is > 0, and thus, χ_*z*_ – χ̅ must be < 0 to be consistent with δ_exp_^para^. This implies easy-plane anisotropy of the magnetic moment, which would indeed be consistent with Ln(III) ions for Ln = Ce, Pr, and Nd with a trigonal planar ligand field dominated by the three anionic equatorial ligands.^87^ However, CASSCF-SO calculations using the XRD structure predict easy-axis anisotropy for **1-Ce**, **1-Pr**, and **1-Nd**, dictated by the axial THF ligands (see above; the outcome does not change for the gas-phase optimized structures), which is indeed confirmed by EPR spectroscopy on solid state samples. This apparent disconnect could be resolved if one or both THF molecules dissociate in solution, which is already suggested by missing solution ^1^H resonances for some of the THF ligands and **1-La** results (see above). Hence, we computationally removed one or both THF ligands(s), optimized the structure in the gas phase, and performed CASSCF-SO pNMR calculations for these structural models to assess if this possibility is consistent with the data.

For **1-Ce**, none of the pNMR shift calculations give a clear agreement with solution experimental data (see Supporting Information Tables S24, S27 and S30); as Ce(III) has the smallest magnetic moment of all Ln(III) ions in the periodic table, it also has the smallest paramagnetic shifts, and hence approximations in our electronic structure method and our gas-phase structural models are insufficient to capture the subtleties of these data. Considering **1-Nd**, both ^1^H and ^29^Si solution pNMR results show discrepancies between the experimental values and the values predicted using the XRD or optimized M(THF)_2_ structures. For the ^1^H resonances, calculations using the XRD structure (optimized structure) predict shifts of −6.4 and ca. –0.5 ppm (−2.3 and ca. –0.4 ppm) for the “in-plane”/group 1 and “out-of-plane”/group 2 signals, respectively, compared to the experimental values of 4.9 and −5.0 ppm, respectively. Upon removing one THF ligand and maintaining the “in-plane”/“out-of-plane” averaging, the calculations give chemical shifts of ca. 5.8 and −4.2 ppm, respectively, or ca. 5.8 and −5.6 ppm when both THF ligands are removed (see Supporting Information Table S26). For the ^29^Si “in-plane”/group 1 resonances (group 2 not observed; now considering only δ_vdH-S_^para^), the predicted shift using the XRD structure (optimized structure) is −22.3 ppm (−11.7 ppm), which varies from the experimental value of 6.3 ppm. When one THF ligand is removed, the shift increases to 8.7 ppm, and then to 8.0 ppm when the second THF ligand is removed (see Supporting Information Table S26). Hence, both the ^1^H and ^29^Si pNMR data suggest a formulation of either Nd(THF)_1_ or Nd(THF)_0_ for **1-Nd** in solution. All chemical shifts show pronounced discrepancy compared to the experiment for the second averaging option (see Supporting Information Table S29), while the third averaging option (see Supporting Information Table S32) is also compatible with Nd(THF)_1_; however, the agreement of calculated values with experiment is not as close as for “in-plane”/“out-of-plane” averaging.

For **1-Pr**, both ^1^H and ^29^Si calculated pNMR results show discrepancies compared to the experimental solution values when employing *bis*-THF structures (see Supporting Information Table S25). Using the XRD structure (optimized structure) for ^1^H gives shifts of ca. −14.5 and ca. −1.9 ppm (ca. −4.4 and ca. −0.5 ppm) for the “in-plane”/group 1 and “out-of-plane”/group 2 signals, respectively, compared to the experimental values of 7.9 and −8.1 ppm, respectively. By removing one THF ligand and maintaining the “in-plane”/“out-of-plane” averaging, the calculations give chemical shifts of ca. 11.0 and ca. −8.0 ppm, or ca. 7.6 and ca. −6.2 ppm when removing the second THF ligand (see Supporting Information Table S25). Hence, the models with some THF lost better align with the experimental data. Similarly, for solution ^29^Si pNMR “in-plane”/group 1 resonances (group 2 not observed; now considering only δ_vdH-S_^para^), the XRD structure (optimized structure) gives a shift of −40.9 ppm (optimized structure: −15.0 ppm), deviating substantially from the experimental value of 10.2 ppm. The shifts for Pr(THF)_1_ and Pr(THF)_0_ are 24.0 and 16.0 ppm, respectively; here, the Pr(THF)_0_ model is in better agreement with the experimental data (see Supporting Information Table S25). Using the second averaging method, the pNMR shifts are predicted adequately using a Pr(THF)_0_ model (see Supporting Information Table S28), or using a Pr(THF)_1_ model with the third averaging method (see Supporting Information Table S31), but in both cases, the calculated parameters are not as close to experiment as for the “in-plane”/“out-of-plane” averaging approach.

We note that in none of the cases are the pNMR shifts of the coordinated ^29^Si_M_ atoms correctly predicted for paramagnetic **1-Ln** and that the choice of structural model has a significant effect on the calculated values (see Supporting Information Tables S24–S26). This indicates substantial influence of the contact spin density based on structural models that we cannot capture with these simplified static models and minimal CASSCF-SO calculations. For paramagnetic **1-Ln**, only one nonmetal-bound ^29^Si resonance is observed, again likely a result of dynamic averaging. The weighted average paramagnetic shifts are 11.8, 33.4, and 57.1 ppm for **1-Ce**, **1-Pr**, and **1-Nd**, respectively, while the calculated pNMR shifts using the XRD geometries and CASSCF-SO methods (δ_vdH-S_^para^, weighted average of nonmetal-bound ^29^Si resonances) are −4.8, −23.6, and −13.8 ppm, for **1-Ce**, **1-Pr**, and **1-Nd**, respectively. Clearly, these values are poorly predicted, which shows that nontrivial spin density is transferred from the metal ions beyond the first coordination sphere and that extensive active space methods would be required to approach experimental accuracy in even these simple complexes.

The ^29^Si NMR chemical shifts of a number of uranium complexes have previously been compiled, and though there is no ^29^Si NMR data of a U(III) hypersilanide complex for comparison, we note that SiMe_3_ groups were assigned in the ^29^Si NMR spectra of [K(18-crown-6)][U{[Si(SiMe_3_)_2_SiMe_2_]_2_O}(THF)_2_(I)_2_] (−50.0 ppm)^45^ and trigonal pyramidal [U{N(SiMe_3_)_2_}_3_] (−219 ppm).^92^ The δ_Si_ values of the SiMe_3_ groups (−6.0 ppm) and metal-bound silicon atoms (−70.5 ppm) of U(THF)_*n*_ (*n* = 0 or 1) are far downfield of most of the previously reported range of δ_Si_ values for U(III) complexes (between −116 and −247 ppm), which tend to exhibit chemical shifts >100 ppm upfield of parent group 1 ligand transfer agents.^92^ The comparatively small paramagnetic shift of the SiMe_3_ groups and the silanides in U(THF)_*n*_ (*n* = 0 or 1) from HSi(SiMe_3_)_3_ (δ_Si_ in C_6_D_6_ = −11.6 ppm, SiMe_3_; −115.6 ppm, HSi)^53^ correlates with the paramagnetic line broadening being relatively minor in the M(THF)_*n*_ (*n* = 0 or 1) series herein, which delivers the first examples of M(III)-bound solution ^29^Si DEPT90 NMR resonances for all of the paramagnetic M herein to the best of our knowledge (we note that a signal was tentatively assigned for the silanide atom in [La(Cp”)_2_{Si(SiMe_3_)_3_}] at δ_Si_ = −130.25 ppm, but data from correlation experiments were ambiguous).^41^

## 4 Conclusions

The rich multinuclear solution and ssNMR spectra of the M(III) *tris*-hypersilanide complexes [M{Si(SiMe_3_)_3_}_3_(THF)_2_] for M = La, Pr, Ce, Nd, and U, coupled with the high local symmetries of their metal sites, has provided a rare opportunity to study paramagnetic shifts in an isostructural series of f-block complexes by ^29^Si NMR spectroscopy. We find by a combination of single crystal XRD and EPR spectroscopy that in the solid state, these complexes show trigonal bipyramidal geometries, with local *D*_3*h*_ symmetries of the central MSi_3_O_2_ cores and easy-axis magnetic anisotropy. The ^29^Si MAS NMR spectra of these complexes each show only one signal for the trimethylsilyl groups; this equivalency indicates that dynamic averaging of these environments occurs at ambient temperature due to free rotation of M–Si bonds. The ^29^Si resonance for the metal-bound silicon atoms is only seen for the diamagnetic La(III) analogue in the solid state, due to the magnetic anisotropy of the paramagnetic M(III) ions broadening the signal into the baseline. Using a combination of characterization methods, we find that the coordinated THF molecules in [M{Si(SiMe_3_)_3_}_3_(THF)_2_] are readily displaced in solution to give the desolvated species [M{Si(SiMe_3_)_3_}_3_(THF)_*n*_] (*n* = 0 or 1), which show local *C*_3*v*_-symmetric cores and easy-plane magnetic anisotropy. The solution ^1^H NMR spectra of [M{Si(SiMe_3_)_3_}_3_(THF)_2_] in 9:1 C_6_D_6_/C_4_D_8_O solutions each show two trimethylsilyl environments in a 1:2 ratio, and for M = La and Ce, we also observe two signals for SiMe_3_ groups in both the ^13^C{^1^H} and ^29^Si DEPT solution NMR spectra. We attribute this observation to restricted rotation of M–Si bonds on the NMR time scale upon loss of THF from coordination spheres; unusually, metal-bound silicon resonances are seen in all solution ^29^Si DEPT90 NMR spectra.

The DFT-calculated ^29^Si NMR shifts of [La{Si(SiMe_3_)_3_}_3_(THF)_2_] and desolvated [La{Si(SiMe_3_)_3_}_3_(THF)_*n*_] (*n* = 0 or 1) showed excellent agreement with experimentally obtained values and were in accord with a dynamic equilibrium of [La{Si(SiMe_3_)_3_}_3_(THF)_2_] and [La{Si(SiMe_3_)_3_}_3_(THF)] persisting in the presence of a large excess of THF. The CASSCF-SO-calculated pNMR shifts of trimethylsilyl groups in paramagnetic [M{Si(SiMe_3_)_3_}_3_(THF)_2_] and desolvated [M{Si(SiMe_3_)_3_}_3_(THF)_*n*_] (*n* = 0 or 1) show reasonable agreement for M = Pr and Nd existing as either [M{Si(SiMe_3_)_3_}_3_(THF)_1_] or [M{Si(SiMe_3_)_3_}_3_] in 9:1 C_6_D_6_/C_4_D_8_O solutions, but assigning the experimental data to one of these two structures is challenging given the likelihood of dynamic equilibria in solution, cf. the La homologue. We also cannot rule out the limitations inherent to the computational method used; for instance, gas-phase optimization does not consider the effect of explicit solvent interaction which is in a huge excess for experimentally obtained data. Furthermore, the experimental chemical shifts represent an average over large number of molecules that move over a relativity long time scale, as opposed to the chemical shift from single stationary molecule optimized in the gas phase as computed here; the time scales required for examining these equilibria are out of reach for the ab initio molecular dynamics that would be required to model the M-THF dissociation processes. Finally, the structural ambiguity and complex nature of the paramagnetic shifts does not allow us to accurately model metal-bound silicon atoms directly for any of the paramagnetic complexes herein.

## 5 Experimental Section

### 5.1 General Methods and Materials

All manipulations were conducted under argon with the strict exclusion of oxygen and water by using Schlenk line and glovebox techniques. Solvents were dried by refluxing over Na/K alloy (diethyl ether) or potassium (hexane) and stored over a potassium mirror and then degassed before use. To make up solution samples for NMR, EPR, and UV–vis–NIR spectroscopy, hexane, toluene, THF, Me-THF, C_6_D_6_, and C_4_D_8_O were dried by refluxing over K and were vacuum transferred and degassed by three freeze–pump–thaw cycles before use. Elemental analysis (C and H) was carried out either by Mr Martin Jennings and Mrs Anne Davies at the Microanalytical service, Department of Chemistry, the University of Manchester, or the Elemental Analysis Services Team, Science Centre, London Metropolitan University. The starting materials [K{Si(SiMe_3_)_3_}],^54^ [MI_3_(THF)_*x*_] (M = La, Ce, Pr, *x* = 4; M = Nd, *x* = 3.5)^93^ and [UI_3_(THF)_4_]^94^ were prepared according to literature procedures. ATR-IR spectra were recorded as microcrystalline powders using a Bruker Alpha spectrometer with Platinum-ATR module. UV–vis–NIR spectroscopy was performed on samples in Youngs tap-appended 10 mm path length quartz cuvettes on an Agilent Technologies Cary Series UV–vis–NIR spectrophotometer from 175 to 3300 nm. Caution: Natural abundance uranium is a weak α-emitter; thus, we recommend the use of suitable designated radiochemical laboratories with α-counting equipment available for safe manipulation of compounds containing this element.

Powder XRD data were obtained on small batches of microcrystalline **1-M** that were suspended in Fomblin oil to prevent sample decomposition from oxygen and water. These samples were mounted on a Micromount and placed on a goniometer head under a cryostream to cool the sample to 100 K, freezing the Fomblin to suspend the crystallites for the duration of the experiment. The PXRD data were measured on a Rigaku FR-X diffractometer, operating in powder diffraction mode using Cu Kα radiation (λ = 1.5418 Å) with a Hypix-6000HE detector and an Oxford Cryosystems nitrogen flow gas system. Data were collected between 3 and 20° θ, with a detector distance of 150 mm and a beam divergence of 1.5 mRad.^95^ X-ray data were collected using CrysAlisPro.^96^ For data processing, the instrument was calibrated using silver behenate as standard. Then, X-ray data were reduced and integrated using CrysAlisPro.^96^ Pawley refinements with the unit cells obtained from the crystal structures were performed using TOPAS.^97,98^

X-ray diffraction data for single crystals of **1-La**, **1-Ce**, **1-Pr**, **1-Nd**, and **1-U** in Fomblin on a Micromount were examined using a Rigaku FR-X diffractometer, equipped with a HyPix 6000HE photon counting pixel array detector with graphite-monochromated Mo Kα (λ = 0.71073 Å) (**1-U**) or Cu Kα (λ = 1.5418 Å) (**1-La**, **1-Ce**, **1-Pr**, **1-Nd**) radiation. Intensities were integrated from data recorded on 1° frames by ω rotation. Cell parameters were refined from the observed positions of all strong reflections in each data set. A Gaussian grid face-indexed with a beam profile was applied for all structures.^96^ The structures were solved using SHELXT;^99^ the data sets were refined by full-matrix least-squares on all unique *F*^2^ values,^100^ with anisotropic displacement parameters for all non-hydrogen atoms and with constrained riding hydrogen geometries; *U*_iso_(H) was set at 1.2 (1.5 for methyl groups) times *U*_eq_ of the parent atom. The largest features in final difference syntheses were close to heavy atoms and were of no chemical significance. CrysAlisPro^96^ was used for control and integration, and SHELX^99,100^ was employed through OLEX2^101^ for structure solution and refinement. ORTEP-3^102^ and POV-Ray^103^ were employed for molecular graphics.

Solution NMR spectra were recorded on a Bruker AVIII HD 400 spectrometer operating at 400.07 (^1^H), 100.60 (^13^C), or 79.48 (^29^Si) MHz; sample concentrations used were ∼50 mM. All solution NMR spectra (^1^H, ^13^C, ^29^Si) were referenced to tetramethylsilane (TMS). Solid-state ^1^H, ^29^Si direct excitation, and {^1^H-}^29^Si cross-polarization (CP) NMR spectra were recorded using a Bruker 9.4 T (400 MHz ^1^H Larmor frequency) AVANCE III spectrometer equipped with a 4 mm HFX MAS probe. Experiments were acquired at ambient temperature using various MAS frequencies. For the frequencies employed (5 to 12 kHz), the sample temperature was determined using an external reference of KBr to be 300 ± 3 K. Samples were packed into 4 mm o.d. zirconia rotors in a glovebox and sealed with a Kel-F rotor cap. The ^1^H (π/2)- and π-pulse durations were 2.5 and 5.0 μs, respectively, and the ^29^Si (π/2)- and π-pulse durations were 5.5 and 11.0 μs, respectively. ^29^Si spin-locking was applied for 4 ms at ∼45 kHz, with corresponding ramped (70–100%) ^1^H spin-locking at 50–60 kHz (depending on MAS frequency) for CPMAS experiments. 100 kHz SPINAL-64^104^ heteronuclear ^1^H decoupling was used throughout signal acquisition. A Hahn-echo τ_r_–π–τ_r_ sequence of two rotor periods total duration was applied to ^29^Si after CP to circumvent receiver dead-time for **1-La**. 1312, 20768, 189440, 61440, and 22796 transients were coadded for **1-La**, **1-Ce**, **1-Pr**, **1-Nd**, and **1-U**, respectively, with repetition delays of 3, 0.2, 0.03, 0.2, and 0.2 s, respectively. Spectral simulations were performed in the solid line-shape analysis (SOLA) module v2.2.4 in Bruker TopSpin v4.0.9. All ssNMR spectra were referenced to TMS (^1^H and ^29^Si). Experiments were acquired at ambient temperature using various MAS frequencies. For the frequencies employed (5 to 12 kHz), the sample temperature was determined using an external reference of KBr to be 301 ± 10 K.

Magnetic measurements were performed on a Quantum Design MPMS3 superconducting quantum interference device (SQUID) magnetometer. Finely ground powder samples (28–36 mg) were restrained in eicosane (14–18 mg) and flame-sealed in a borosilicate tube under vacuum (see Supporting Information Table S5). Sealed samples were loaded into plastic straws and held in place by friction between diamagnetic tape at the top of the tube and the straw. Raw magnetic data were scaled for the shape of the sample using Quantum Design MPMS3 Geometry Correction Simulator, corrected for the diamagnetic contribution of the sample holder (straw and borosilicate tube) and corrected for the mass of eicosane. The molar susceptibility was corrected for the intrinsic diamagnetic contribution of the sample, estimated as the molecular weight (g mol^–1^) multiplied by 0.5 × 10^–6^ cm^3^ K mol^–1^.^105^ Measurements were performed in DC scan mode with 40 mm scan length, except susceptibility measurements on **1-Ce** and **1-Pr** which were performed in VSM mode with 5 mm amplitude. Susceptibility measurements were performed on cooling in 5 kOe (**1-Ce**) or 1 kOe (**1-Pr**, **1-Nd**, **1-U**) DC field. Hysteresis measurements were performed at 2 K in continuous sweep mode with sweep rates of 91 Oe s^–1^ (2 < |*H*| < 7 T), 54 Oe s^–1^ (1 < |*H*| < 2 T) and 22 Oe s^–1^ (|*H*| < 1 T).

Continuous wave electron paramagnetic resonance (EPR) spectra were recorded at X-band (ca. 9.4 GHz) frequency on a Bruker EMXPlus spectrometer with 1.8 T electromagnet and Stinger closed-cycle helium gas cryostat. Powders of **1-Ce**, **1-Nd**, and **1-U** were finely ground in a glovebox, and 1–2 cm of each sample was loaded into to a 4 mm outer diameter (OD) quartz tube and sealed under vacuum. A solution of **1-Nd** in 2-Me-THF (15 mM) was prepared in a glovebox. Quartz tubes of OD 4 mm were filled with ∼3 cm of solution, which was immediately frozen and flame-sealed under vacuum, and then rapidly transferred to the spectrometer within ca. 15 min. Spectra were obtained at base temperature (4–7 K), and powder spectra were obtained for two rotations at ∼90° to one another to identify any features due to polycrystallinity. The field was corrected using a strong pitch sample (*g* = 2.0028). Spectra were simulated in EasySpin 6.0.0-dev.51.^64^ Powder spectra of **1-Ce** and **1-Nd** were simulated in the EasySpin function pepper as an effective *S* = 1/2, with rhombic *g* values, *g* = [*g*_1_, *g*_2_, *g*_3_] and *g*Strains (distribution of *g* values) to account for all line broadenings effects. For **1-Nd**, *g*_*3*_ was not observed (<0.4) and so was fixed to 0.2. Hyperfine coupling on the *g*_1_ feature (*A*_1_) for **1-Nd** was included for the most abundant isotope and scaled for other isotopes based on nuclear *g*-factors.

The H-only structural optimization of diamagnetic **1-La**, and full geometry optimization of desolvated La(THF)_1_ and La(THF)_0_ analogues were performed using ADF 2017;^65−67^ structural optimization of paramagnetic **1-M** (where performed) and desolvated M(THF)_1_ and M(THF)_0_ analogues were conducted in the gas phase using DFT with Gaussian 16,^106^ and electron correlation were described using the PBE functional.^107^ All central lanthanide atoms were treated with the Stuttgart RSC-ANO ECP basis set,^108−110^ and all remaining atoms with cc-pVDZ.^111^ Diamagnetic NMR chemical shifts were computed with ADF 2017.^65−67^ Spin–orbit relativistic, single-point calculations, using the optimized geometries described above, employed either the BP86, PBE0, SAOP, or B3LYPHFXX (XX = 10, 15, 20, 25, 30, 35, 40, 45, 50) hybrid functionals. All-electron Slater-type orbital triple-ζ quality basis sets (TZ2P) were employed in conjunction with the two-component zero-order regular approximation (ZORA) Hamiltonian.^112−114^ The ^29^Si NMR chemical shifts are reported relative to TMS. Scalar relativistic approaches (spin–orbit neglected) were used within the ZORA Hamiltonian to include relativistic effects,^112,113,115^ and a benzene solvent continuum was added. The local density approximation (LDA) with a correlation potential was used in all calculations.^116^ Generalized gradient approximation corrections were performed using the functionals of Becke and Perdew.^117,118^ NBO analysis was carried out using NBO6.^82^ QTAIM analysis^83,84^ was performed within the ADF package; the MOs and NBOs were visualized using ADFView.^66,67^

CASSCF-SO calculations were performed with OpenMolcas^77^ on XRD structures of paramagnetic **1-M**. Basis sets from the ANO-RCC library^119−121^ were used with VTZP quality on the metal atom, VDZP quality on the coordinating atoms and VDZ quality on all other atoms, employing the second-order DKH transformation. Cholesky decomposition of the two-electron integrals with a threshold of 10^–8^ was performed to save disk space and reduce computational demand. The molecular orbitals (MOs) were averaged in state-averaged CASSCF calculations with active spaces of (1,7) (**1-Ce**), (2,7) (**1-Pr**), and (3,7) (**1-Nd** and **1-U**) averaging over seven doublets (**1-Ce**), 21 triplets and 28 singlets (**1-Pr**), and 35 quartets and 112 doublets (**1-Nd** and **1-U**). The spin-free wave functions obtained from these CASSCF calculations were mixed by spin–orbit coupling in the RASSI module. The *g*-values, magnetization, and magnetic susceptibility (isotropic value and tensor) were calculated using SINGLE_ANISO,^122^ and the spin–orbit wave functions were decomposed into their crystal field wave functions, with the quantization axis defined by the *g*_1_ direction in the ground doublet. From the magnetic susceptibility tensor, we calculate the pseudocontact shift (δ_PCS_^para^ for the ^29^Si and ^1^H nuclei as
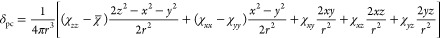
where χ̅ = 1/3Tr(χ).^89^ To calculate δ_PCS_^para^, we have implemented the paramagnetic component of the van den Heuvel and Soncini method^47^ in our HYPERION code,^123^ which uses a full sum-over-states expression derived from the derivative of the Helmholtz free energy
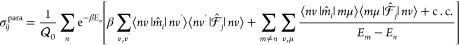
where the paramagnetic shielding tensor σ_*ij*_^para^ is related to the paramagnetic shift by , *m̂* and  are the Zeeman and hyperfine operators, respectively, and are levels with degenerate states *v*, μ, respectively.

### 5.2 General Procedure for the Synthesis of **1-M**

A Schlenk flask was charged with [MI_3_(THF)_*x*_] (M = La, Ce, Pr, U, *x* = 4; M = Nd, *x* = 3.5) and diethyl ether (20 mL/mmol) and was cooled to −78 °C. [K{Si(SiMe_3_)_3_}] (3 equiv) was dissolved in diethyl ether (5 mL/mmol) in another Schlenk flask and was added dropwise to the cooled [MI_3_(THF)_*x*_] suspension with stirring. The yellow reaction mixture was stirred for 1 h at −78 °C and then allowed to warm to room temperature. During this time, the color of the reaction mixture either remained yellow (M = La) or darkened to orange (M = Ce, Pr), brown (M = Nd), or dark green (M = U). After reaching room temperature, all volatiles were immediately removed in vacuo and the product was extracted with hexane (40 mL/mmol). Concentration and storage of the filtrate at −25 °C led to the formation of needles of [M{Si(SiMe_3_)_3_}_3_(THF)_2_] (**1-M**).

#### 5.2.1 [La{Si(SiMe_3_)_3_}_3_(THF)_2_] (**1-La**)

Prepared according to the general procedure with [LaI_3_(THF)_4_] (0.744 g, 1 mmol) and [K{Si(SiMe_3_)_3_}] (0.860 g, 3 mmol); **1-La** was obtained as yellow needles (0.425 g, 0.41 mmol, 41%). Anal. Calcd for C_35_H_97_LaO_2_Si_12_: C, 40.97; H, 9.53. Found: C, 39.99; H, 9.83. ^1^H NMR (C_6_D_6_/C_4_D_8_O (9:1), 400 MHz): δ 3.62 (OC*H*_2_CH_2_), 1.46 (OCH_2_C*H*_2_), 0.41 (s, 54H, Si(C*H*_3_)_3_ group 2), 0.23 (s, 27H, Si(C*H*_3_)_3_ group 1). ^13^C{^1^H} NMR (C_6_D_6_/C_4_D_8_O (9:1), 101 MHz): δ 68.18 (O*C*H_2_CH_2_), 25.80 (OCH_2_*C*H_2_), 6.78 (Si(*C*H_3_)_3_ group 2), 1.39 (Si(*C*H_3_)_3_ group 1). ^29^Si{^1^H} NMR (C_6_D_6_/C_4_D_8_O (9:1), 79 MHz): δ −5.3 (*Si*Me_3_ group 2), −13.1 (*Si*Me_3_ group 1), −82.3 (La*Si*). ATR-IR (microcrystalline, cm^–1^) ν̃: 2945 (m), 2891 (m), 1237 (s), 1004 (w), 820 (s), 743 (w), 676 (m), 622 (m), 421 (m). UV–vis–NIR (2 mM in THF, cm^–1^) υ̃ _max_: 27,150 (ε = 1760 M^–1^ cm^–1^).

#### 5.2.2 [Ce{Si(SiMe_3_)_3_}_3_(THF)_2_] (**1-Ce**)

Prepared according to the general procedure with [CeI_3_(THF)_4_] (0.745 g, 1 mmol) and [K{Si(SiMe_3_)_3_}] (0.860 g, 3 mmol); **1-Ce** was obtained as orange needles (0.425 g, 0.41 mmol, 41%). Anal. Calcd for C_35_H_97_CeO_2_Si_12_: C, 40.92; H, 9.52. Found: C, 37.01; H, 9.02. ^1^H NMR (C_6_D_6_/C_4_D_8_O (9:1), 400 MHz): δ 3.74 (OC*H*_2_CH_2_), 1.55 (s, 27H, Si(C*H*_3_)_3_ group 1), 1.51 (OCH_2_C*H*_2_), −1.43 (s, 54H, Si(C*H*_3_)_3_ group 2). ^13^C{^1^H} NMR (C_6_D_6_/C_4_D_8_O (9:1), 101 MHz): δ 68.18 (O*C*H_2_CH_2_), 25.95 (OCH_2_*C*H_2_), 6.30 (Si(*C*H_3_)_3_ group 2), 2.78 (Si(*C*H_3_)_3_ group 1). ^29^Si{^1^H} NMR (C_6_D_6_/C_4_D_8_O (9:1), 79 MHz): δ −6.4 (*Si*Me_3_ group 2), −11.4 (*Si*Me_3_ group 1), −79.4 (Ce*Si*). ATR-IR (microcrystalline, cm^–1^) ν̃: 2945 (m), 2891 (m), 1237 (s), 1004 (w), 820 (s), 743 (w), 676 (m), 622 (m), 421 (m). UV–vis–NIR (2 mM in THF, cm^–1^) υ̃_max_: 26,750 (ε = 1010 M^–1^ cm^–1^), 23,300 (ε = 240 M^–1^ cm^–1^).

#### 5.2.3 [Pr{Si(SiMe_3_)_3_}_3_(THF)_2_] (**1-Pr**)

Prepared according to the general procedure with [PrI_3_(THF)_4_] (0.747 g, 1 mmol) and [K{Si(SiMe_3_)_3_}] (0.860 g, 3 mmol); **1-Pr** was obtained as yellow needles (0.362 g, 0.35 mmol, 35%). Anal. Calcd for C_35_H_97_PrO_2_Si_12_: C, 40.89; H, 9.51. Found: C, 36.84; H, 9.18. ^1^H NMR (C_6_D_6_/C_4_D_8_O (9:1), 400 MHz): δ 8.13 (s, 27H, Si(C*H*_3_)_3_ group 1), −7.66 (s, 54H, Si(C*H*_3_)_3_ group 2), OC*H*_2_CH_2_ and OCH_2_C*H*_2_ signals not observed. ^13^C{^1^H} NMR (C_6_D_6_/C_4_D_8_O (9:1), 101 MHz): δ 9.74 (Si(*C*H_3_)_3_, group 1), O*C*H_2_CH_2_, OCH_2_*C*H_2_ and Si(*C*H_3_)_3_ group 2 signals not observed. ^29^Si{^1^H} NMR (C_6_D_6_/C_4_D_8_O (9:1), 79 MHz): δ –2.9 (*Si*Me_3_, group 1), −65.5 (Pr*Si*), *Si*(CH_3_)_3_ group 2 signals not observed. ATR-IR (microcrystalline, cm^–1^) ν̃: 2945 (m), 2891 (m), 1237 (s), 1004 (w), 820 (s), 743 (w), 676 (m), 622 (m), 421 (m). UV–vis–NIR (2 mM in THF, cm^–1^) υ̃_max_: no transitions observed, tail from 22,200 cm^–1^.

#### 5.2.4 [Nd{Si(SiMe_3_)_3_}_3_(THF)_2_] (**1-Nd**)

Prepared according to the general procedure with [NdI_3_(THF)_3.5_] (0.721 g, 1 mmol) and [K{Si(SiMe_3_)_3_}] (0.860 g, 3 mmol); **1-Nd** was obtained as red needles (0.336 g, 0.33 mmol, 33%). Anal. Calcd for C_35_H_97_NdO_2_Si_12_: C, 40.76; H, 9.48. Found: C, 38.42; H, 9.46. ^1^H NMR (C_6_D_6_/C_4_D_8_O (9:1), 400 MHz): δ 5.08 (s, 27H, Si(C*H*_3_)_3_ group 1), −4.63 (br, s, fwhm = 69.5 Hz, 54H, Si(C*H*_3_)_3_ group 2), OC*H*_2_CH_2_ and OCH_2_C*H*_2_ signals not observed. ^13^C{^1^H} NMR (C_6_D_6_/C_4_D_8_O (9:1), 101 MHz): δ 6.51 (Si(*C*H_3_)_3_, group 1), O*C*H_2_CH_2_, OCH_2_*C*H_2_ and Si(*C*H_3_)_3_ group 2 signals not observed. ^29^Si{^1^H} NMR (C_6_D_6_/C_4_D_8_O (9:1), 79 MHz): δ –6.8 (*Si*Me_3_, group 1), −71.6 (Nd*Si*), *Si*(CH_3_)_3_ group 2 signals not observed. ATR-IR (microcrystalline, cm^–1^) ν̃: 2945 (m), 2891 (m), 1237 (s), 1004 (w), 820 (s), 743 (w), 676 (m), 622 (m), 421 (m). UV–vis–NIR (2 mM in THF, cm^–1^) υ̃_max_: 17,250 (ε = 30 M^–1^ cm^–1^, ^4^I_9/2_ → ^4^G_5/2_).

#### 5.2.5 [U{Si(SiMe_3_)_3_}_3_(THF)_2_] (**1-U**)

Prepared according to the general procedure with [UI_3_(THF)_4_] (0.843 g, 1 mmol) and [K{Si(SiMe_3_)_3_}] (0.860 g, 3 mmol); **1-U** was obtained as dark green needles (0.569 g, 0.51 mmol, 51%). Anal. Calcd for C_35_H_97_UO_2_Si_12_: C, 37.36; H, 8.69. Found: C, 35.48; H, 8.59. ^1^H NMR (C_6_D_6_/C_4_D_8_O (9:1), 400 MHz): δ 5.63 (s, 27H, Si(C*H*_3_)_3_ group 1), 4.24 (OC*H*_2_CH_2_), 1.75 (OCH_2_C*H*_2_), −6.84 (br, s, fwhm = 65.4 Hz, 54H, Si(C*H*_3_)_3_ group 2). ^13^C{^1^H} NMR (C_6_D_6_/C_4_D_8_O (9:1), 101 MHz): δ 25.71 (OCH_2_*C*H_2_), 7.05 (Si(*C*H_3_)_3_, group 1), OCH_2_*C*H_2_ and Si(*C*H_3_)_3_ group 2 signals not observed. ^29^Si{^1^H} NMR (C_6_D_6_/C_4_D_8_O (9:1), 79 MHz): δ –6.0 (*Si*Me_3_, group 1), −70.5 (U*Si*), *Si*(CH_3_)_3_ group 2 signals not observed. ATR-IR (microcrystalline, cm^–1^) ν̃: 2945 (m), 2891 (m), 1237 (s), 1004 (w), 820 (s), 743 (w), 676 (m), 622 (m), 421 (m). UV–vis–NIR (2 mM in THF, cm^–1^) υ̃_max_: 20,500 (ε = 1420 M^–1^ cm^–1^), 19,500 (ε = 1150 M^–1^ cm^–1^), 18,000 (ε = 1190 M^–1^ cm^–1^), 17,100 (ε = 1050 M^–1^ cm^–1^), 16,200 (ε = 930 M^–1^ cm^–1^), 15,700 (ε = 830 M^–1^ cm^–1^), 13,650 (ε = 790 M^–1^ cm^–1^), 12,750 (ε = 560 M^–1^ cm^–1^), 12,000 (ε = 380 M^–1^ cm^–1^), 11,250 (ε = 250 M^–1^ cm^–1^), 10,050 (ε = 90 M^–1^ cm^–1^), 9500 (ε = 80 M^–1^ cm^–1^), 8650 (ε = 120 M^–1^ cm^–1^).

## Supporting Information Available

Additional experimental details, materials, methods, and data (PDF)
