# Supplementary material for: Tris-Silanide f-Block Complexes: Insights into Paramagnetic Influence on NMR Chemical Shifts
Source: JACS Au. 2024 Jul 5;4(7):2695–711. doi: 10.1021/jacsau.4c00466 (PMC11267535; doi:10.1021/jacsau.4c00466)
Supplement: Supplementary file 1 — au4c00466_si_001.pdf [file au4c00466_si_001.pdf]

- *Supporting Information* -

Tris-Silanide f-Block Complexes: Insights into Paramagnetic Influence on NMR Chemical Shifts

Benjamin L. L. Réant,[1] Fraser J. Mackintosh,[1] Gemma K. Gransbury,[1] Carlo Andrea Mattei,[1] Barak Alnami,[1] Benjamin E. Atkinson,[1] Katherine L. Bonham,[2] Jack Baldwin,[1] Ashley J. Wooles,[1] Iñigo J. Vitorica-Yrezabal,[1] Daniel Lee,[2,]* Nicholas F. Chilton,[1,3,]* Stephen T. Liddle,[1,]* and David P. Mills[1,]*

[1]*Department of Chemistry, The University of Manchester, Oxford Road, Manchester, M13 9PL, U.K.*

[2]*Department of Chemical Engineering, The University of Manchester, Oxford Road, Manchester, M13 9PL, U.K.*

[3]*Research School of Chemistry, The Australian National University, Sullivans Creek Road, Canberra, 2601, ACT, Australia.*

**Email: daniel.lee@manchester.ac.uk; nicholas.chilton@anu.edu.au; steve.liddle@manchester.ac.uk; david.mills@manchester.ac.uk*

Contents

1. Solution NMR Spectroscopy S2
1.1. ^{1}H NMR Spectra of 1-M S2
1.2. ^{29}Si DEPT90 NMR Spectra of 1-M S4
1.3. ^{13}C{^{1}H} NMR Spectra of 1-M S6
1.4. ^{1}H-^{29}Si HMBC NMR Spectra of 1-M S8
1.5. Other 2D NMR Spectra of 1-M S10
1.6. Solvent Stability S13
1.7. Use of neat C_4D_8O *vs.* C_6D_6/C_4D_8O (9:1) S15
2. Solid-state NMR Spectroscopy S18
3. ATR-IR Spectroscopy S23
4. UV-Vis-NIR Spectroscopy S26
5. Single Crystal X-ray Diffraction S29
6. Powder X-ray Diffraction S32
7. Magnetism S38
8. EPR Spectroscopy S40
9. Density Functional Theory (DFT) Calculations S41
10. Complete Active Space Self-Consistent Field (CASSCF) Calculations S54
11. pNMR Calculations S57
12. References S65

1. Solution NMR Spectroscopy

Table S1. Experimental NMR parameters for the ^{1}H, ^{13}C and ^{29}Si spectra reported in this study.

Parameter	Nuclei		
	^{1}H	^{13}C	^{29}Si
NS[a]	16	16	128
D1[b]	0.1	2.0	5.0
SW[c]	130	320	400
O1P[d]	– 5	115	– 25

[a] NS = number of scans.

[b] D1 = relaxation decay time in s.

[c] SW = sweep width in ppm.

[d] O1P = irradiation (carrier) frequency offset in ppm

1.1. ^{1}H NMR Spectra of 1-M - α = unidentifiable species, Δ = $HSi(SiMe_3)_3$ impurity.

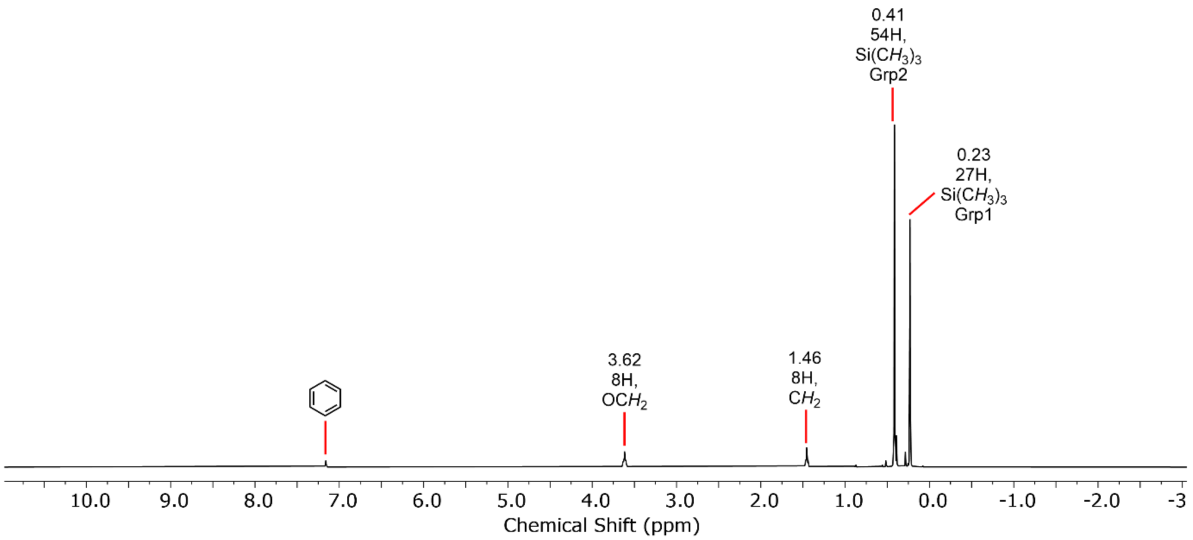

Figure S1. ^{1}H NMR spectrum (400.07 MHz) of **1-La** in C_6D_6/C_4D_8O (9 : 1 by volume).

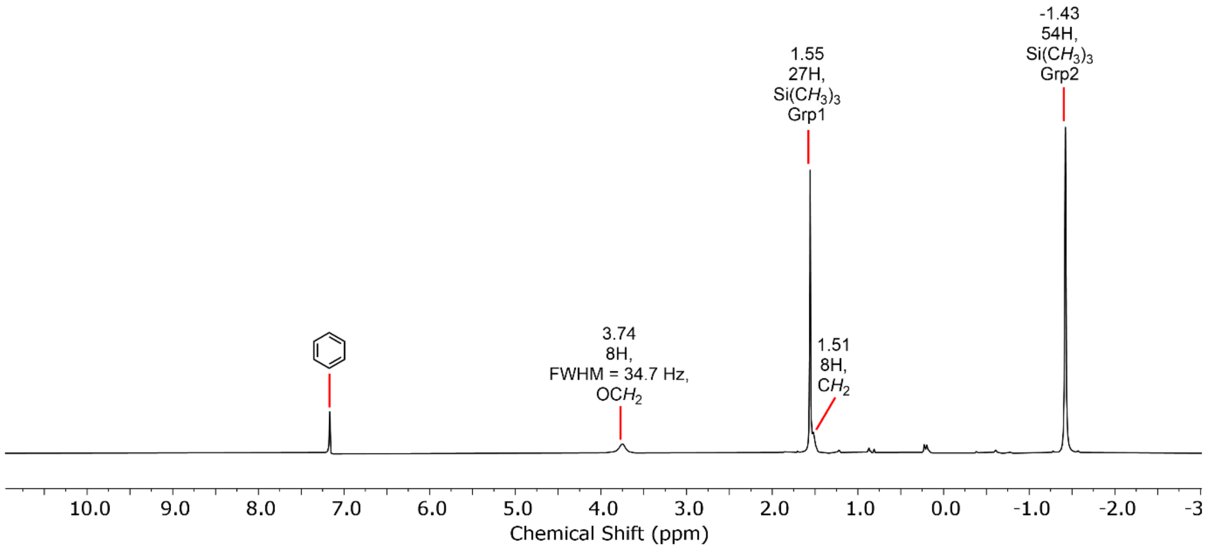

Figure S2. ^{1}H NMR spectrum (400.07 MHz) of **1-Ce** in C_6D_6/C_4D_8O (9 : 1 by volume).

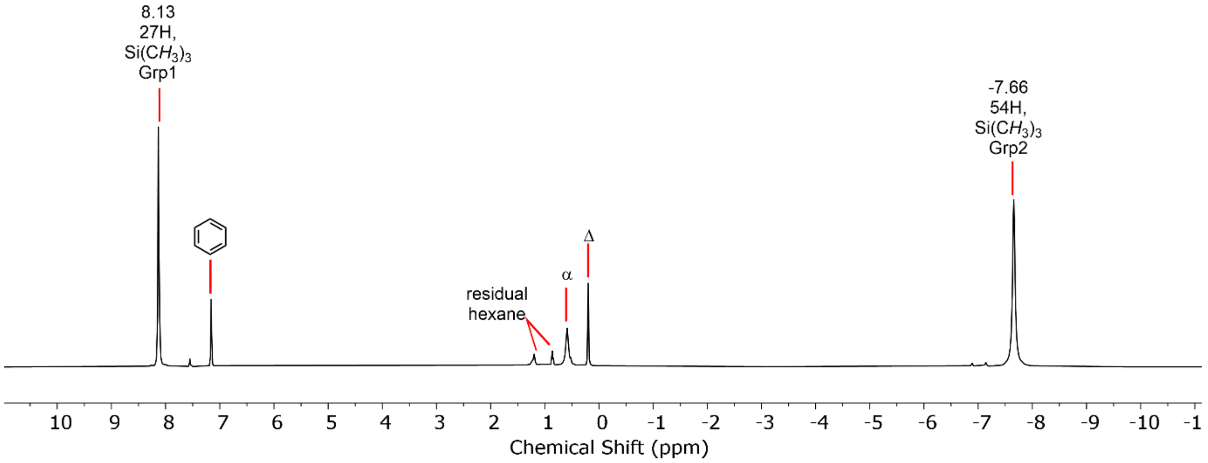

Figure S3. ^{1}H NMR spectrum (400.07 MHz) of **1-Pr** in C_6D_6/C_4D_8O (9 : 1 by volume).

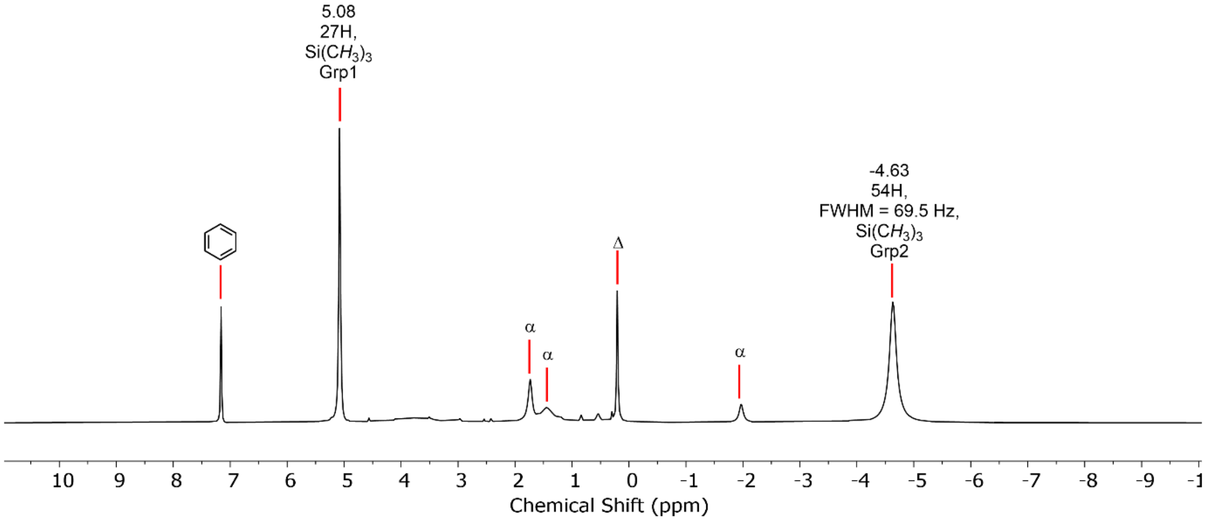

Figure S4. ^{1}H NMR spectrum (400.07 MHz) of **1-Nd** in C_6D_6/C_4D_8O (9 : 1 by volume).

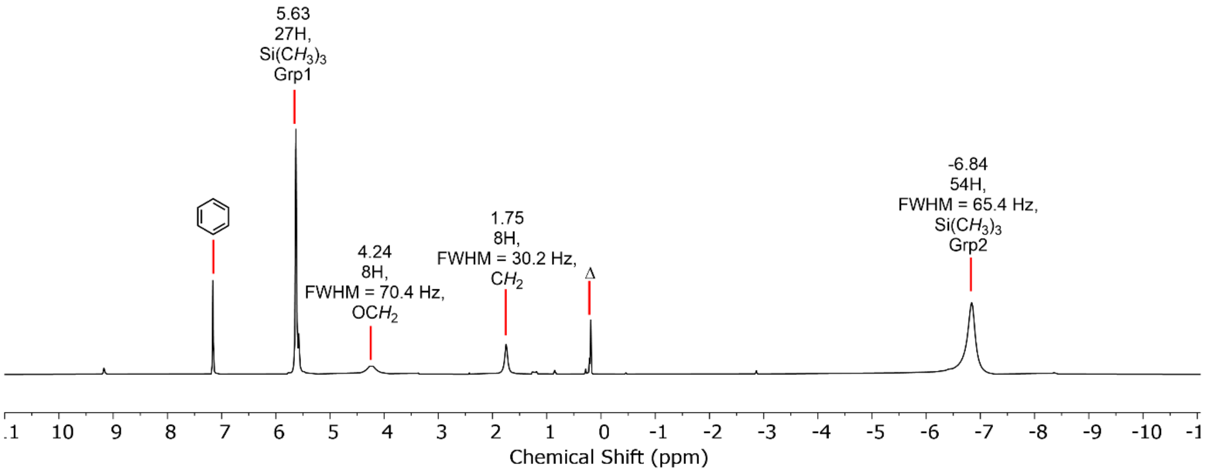

Figure S5. ^{1}H NMR spectrum (400.07 MHz) of **1-U** in C_6D_6/C_4D_8O (9 : 1 by volume).

1.2. ^{29}Si DEPT90 NMR Spectra of 1-M - α = unidentifiable species, Δ = $HSi(SiMe_3)_3$ impurity, * = silicone grease impurity.

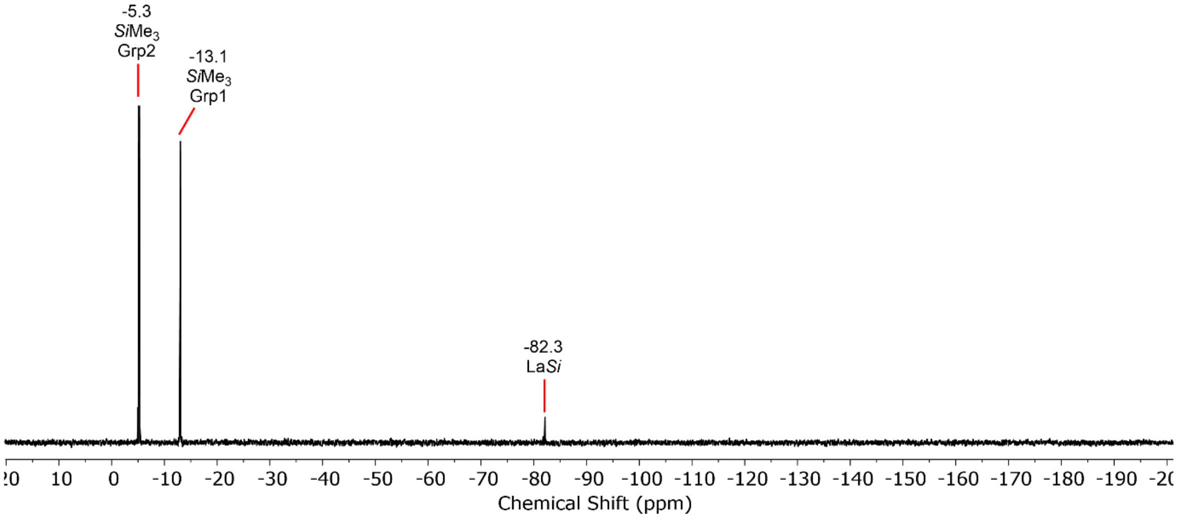

Figure S6. ^{29}Si DEPT90 NMR spectrum (79.48 MHz) of **1-La** in C_6D_6/C_4D_8O (9 : 1 by volume).

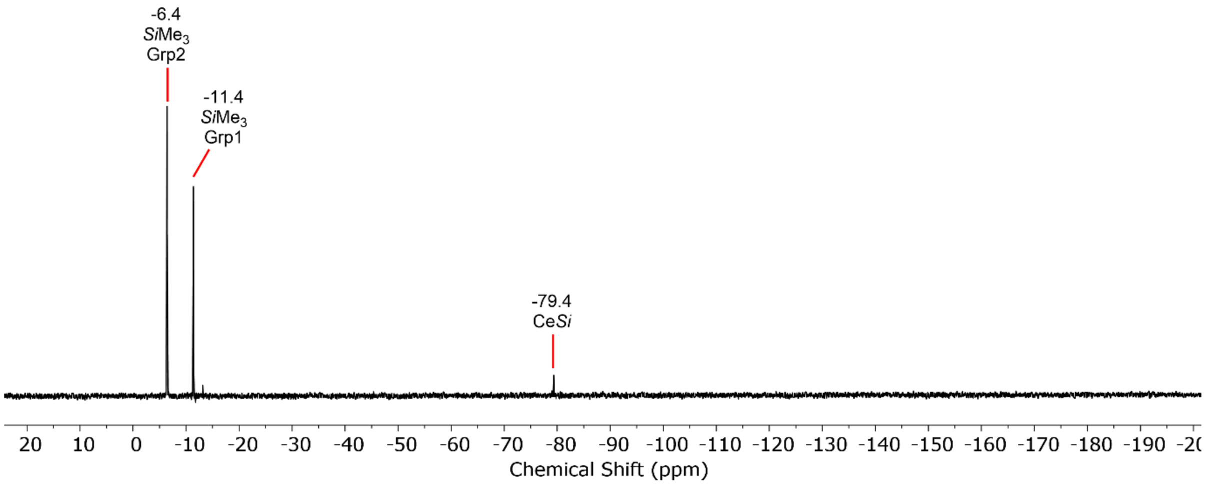

Figure S7. ^{29}Si DEPT90 NMR spectrum (79.48 MHz) of **1-Ce** in C_6D_6/C_4D_8O (9 : 1 by volume).

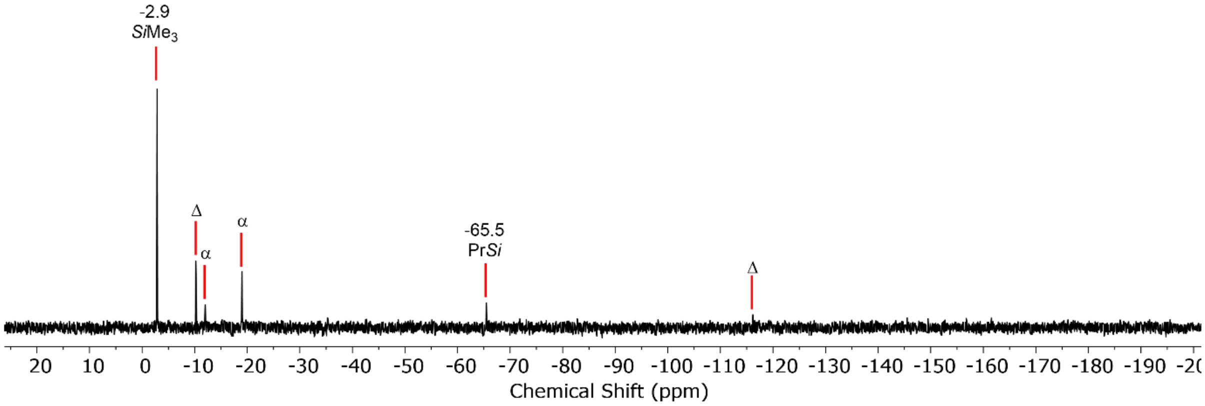

Figure S8. ^{29}Si DEPT90 NMR spectrum (79.48 MHz) of **1-Pr** in C_6D_6/C_4D_8O (9 : 1 by volume).

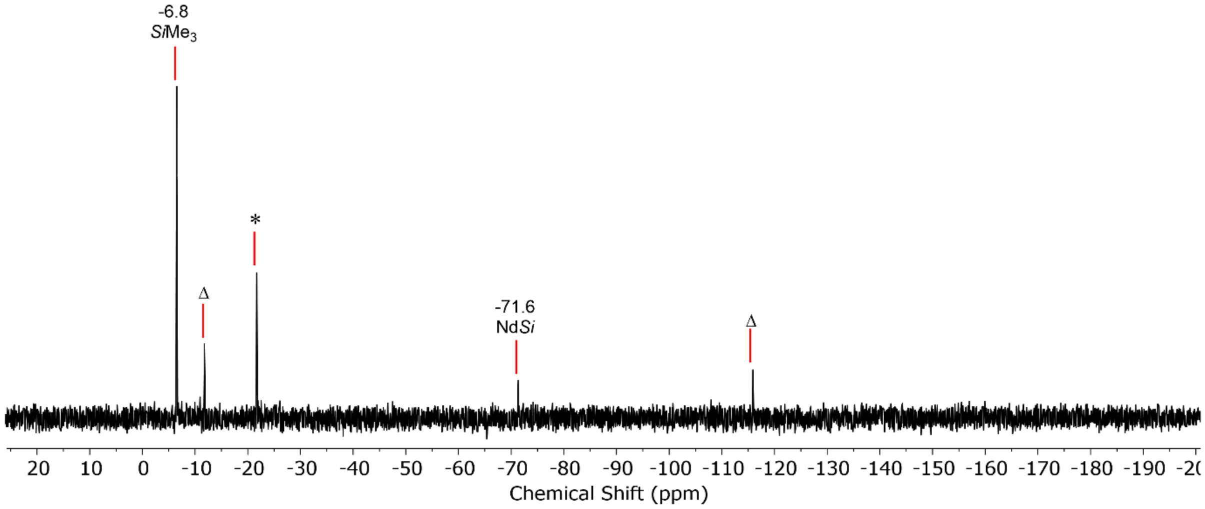

Figure S9. ^{29}Si DEPT90 NMR spectrum (79.48 MHz) of **1-Nd** in C_6D_6/C_4D_8O (9 : 1 by volume).

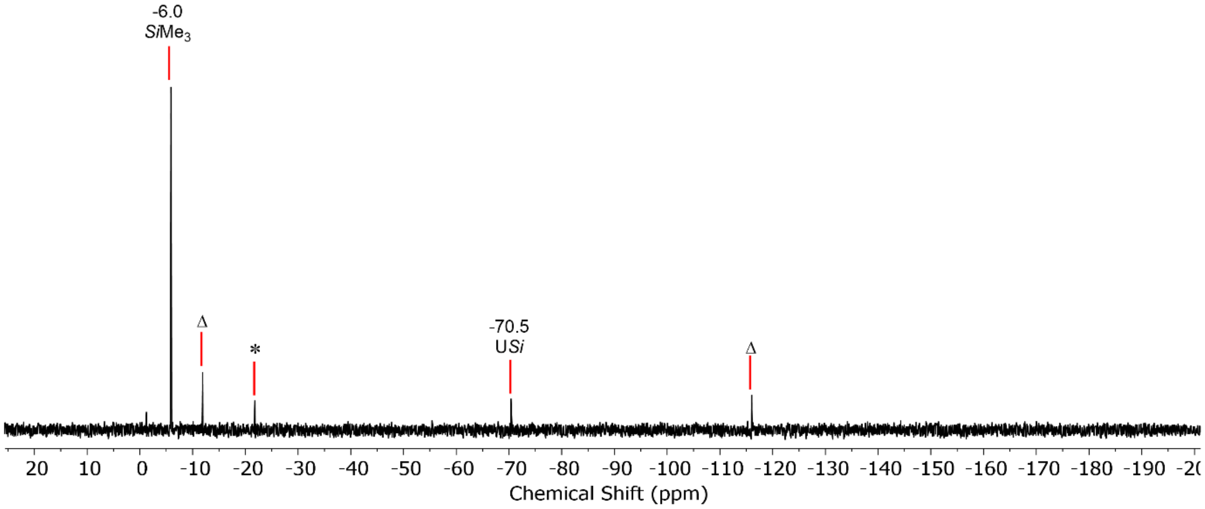

Figure S10. ^{29}Si DEPT90 NMR spectrum (79.48 MHz) of **1-U** in C_6D_6/C_4D_8O (9 : 1 by volume).

1.3. $^{13}C\{^1H\}$ NMR Spectra of 1-M - α = unidentifiable species, Δ = $HSi(SiMe_3)_3$ impurity.

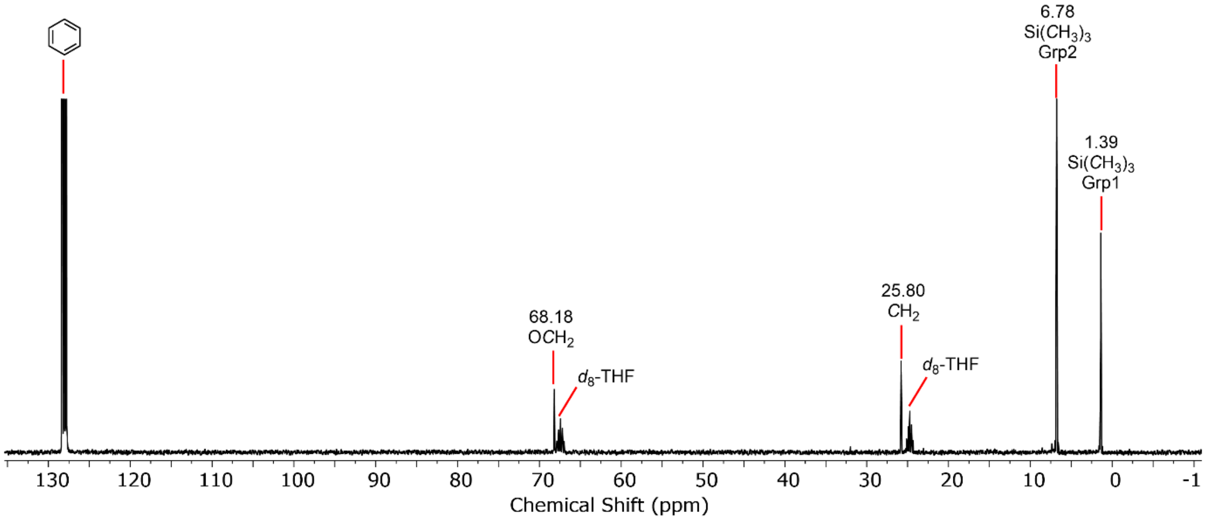

Figure S11. $^{13}C\{^1H\}$ NMR spectrum (125.77 MHz) of **1-La** in C_6D_6/C_4D_8O (9 : 1 by volume).

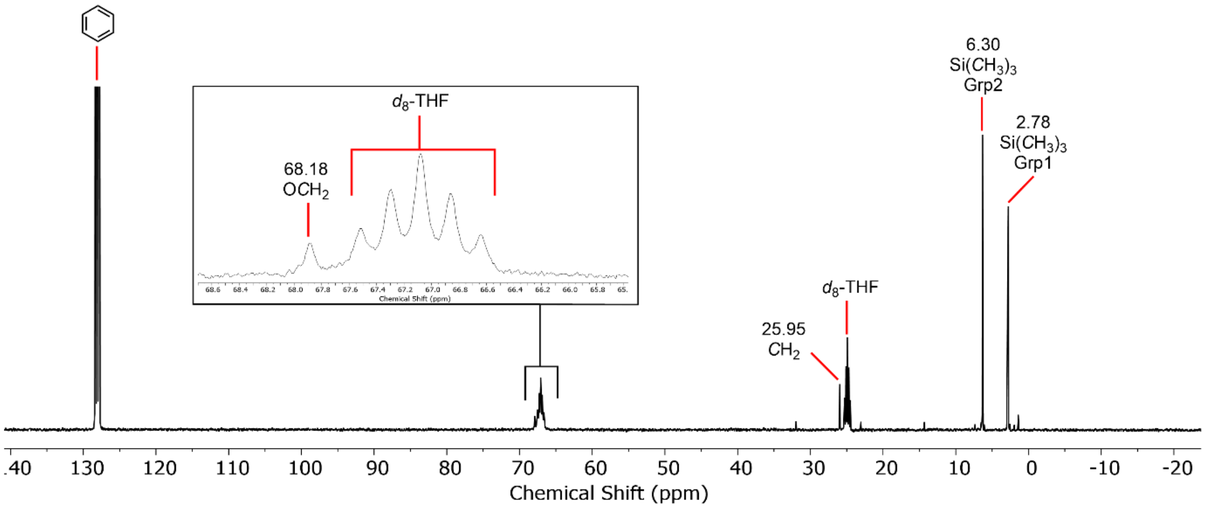

Figure S12. $^{13}C\{^1H\}$ NMR spectrum (125.77 MHz) of **1-Ce** in C_6D_6/C_4D_8O (9 : 1 by volume).

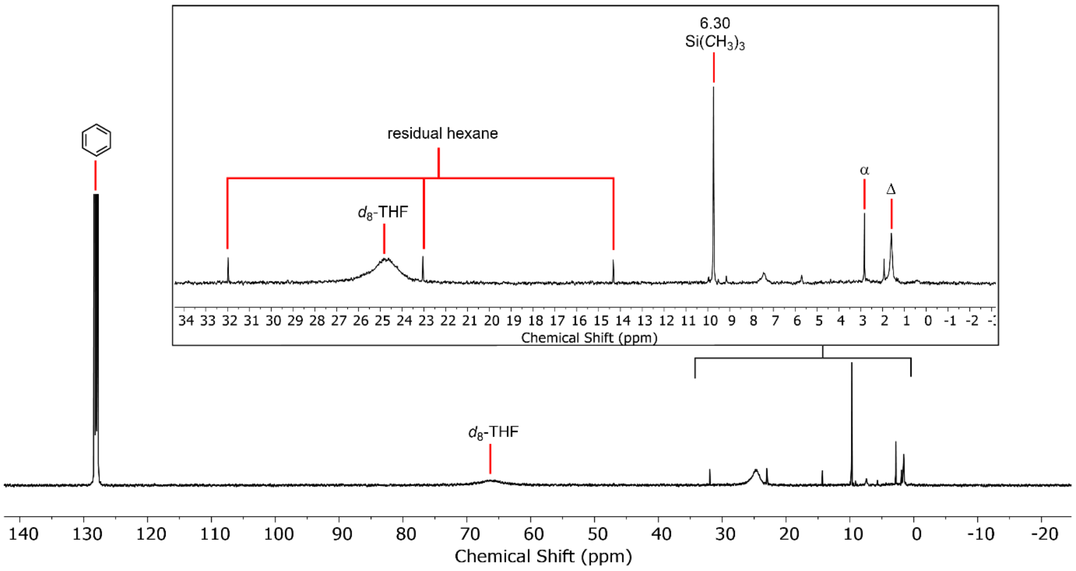

Figure S13. $^{13}C\{^1H\}$ NMR spectrum (125.77 MHz) of **1-Pr** in C_6D_6/C_4D_8O (9 : 1 by volume).

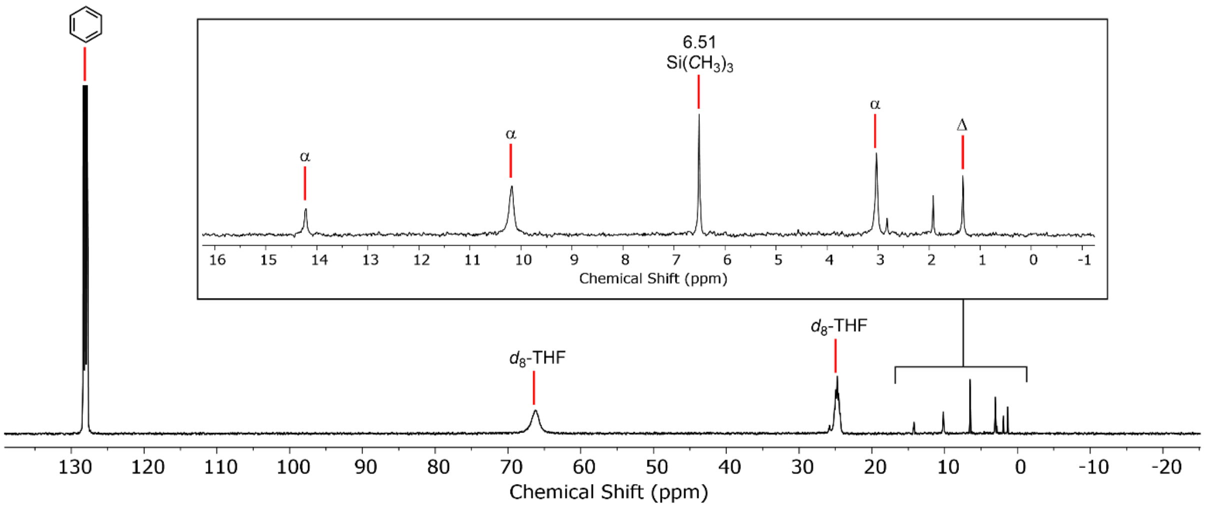

Figure S14. $^{13}C\{^{1}H\}$ NMR spectrum (125.77 MHz) of **1-Nd** in C_6D_6/C_4D_8O (9 : 1 by volume).

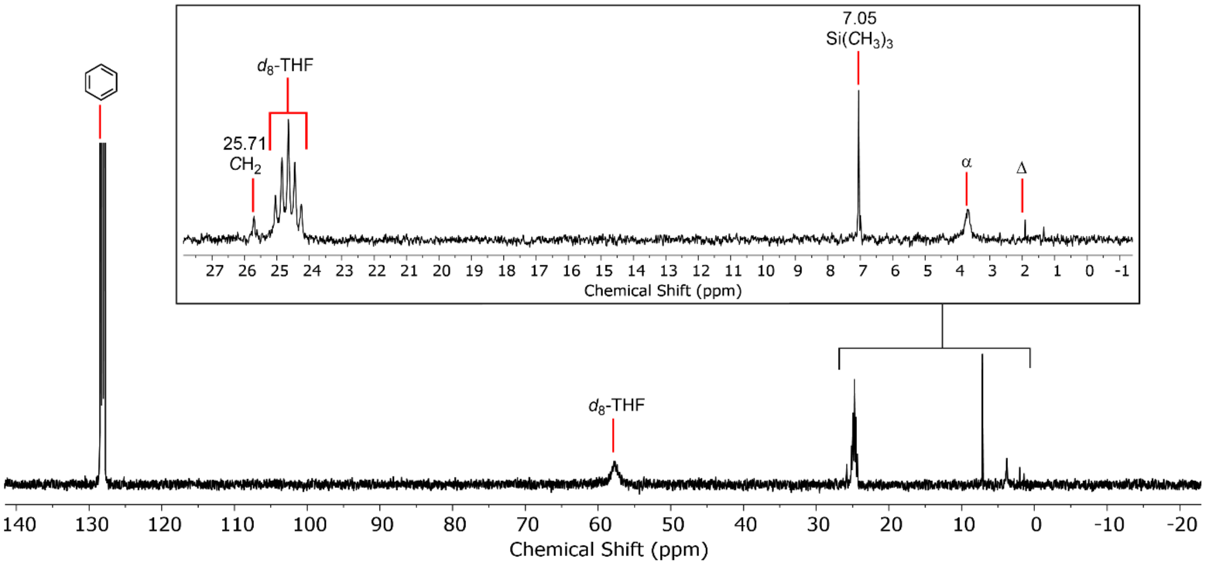

Figure S15. $^{13}C\{^{1}H\}$ NMR spectrum (125.77 MHz) of **1-U** in C_6D_6/C_4D_8O (9 : 1 by volume).

1.4. ^{1}H-^{29}Si HMBC NMR Spectra of 1-M

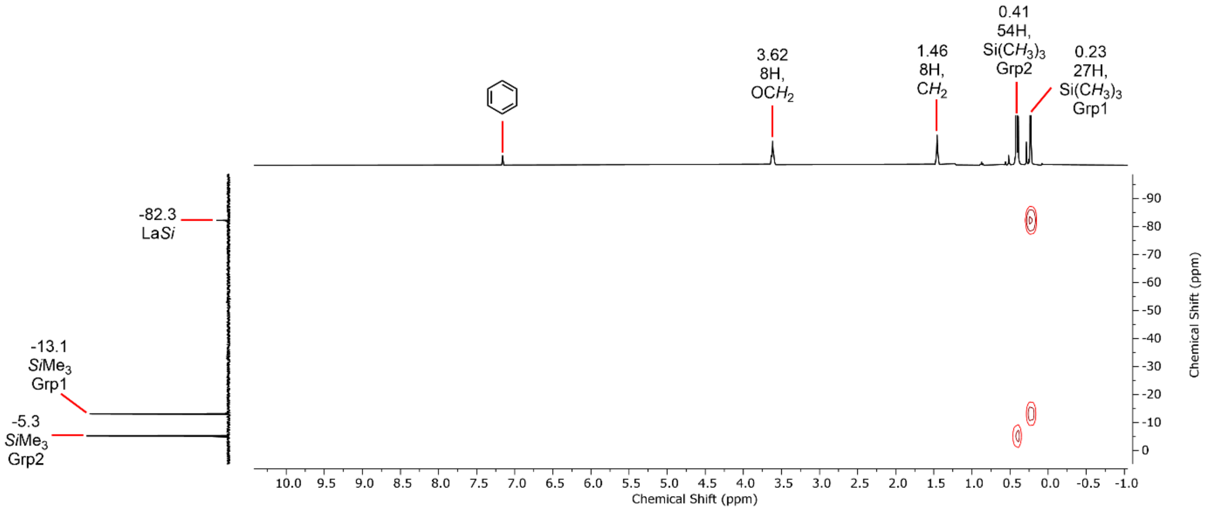

Figure S16. ^{1}H–^{29}Si HMBC NMR spectrum of **1-La** in C_6D_6/C_4D_8O (9 : 1 by volume).

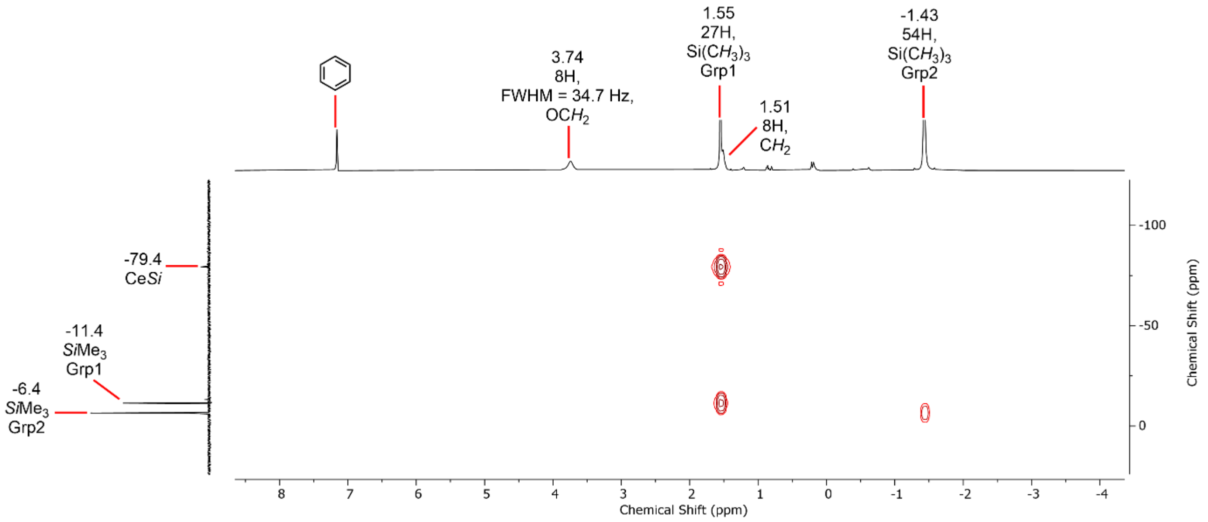

Figure S17. ^{1}H–^{29}Si HMBC NMR spectrum of **1-Ce** in C_6D_6/C_4D_8O (9 : 1 by volume).

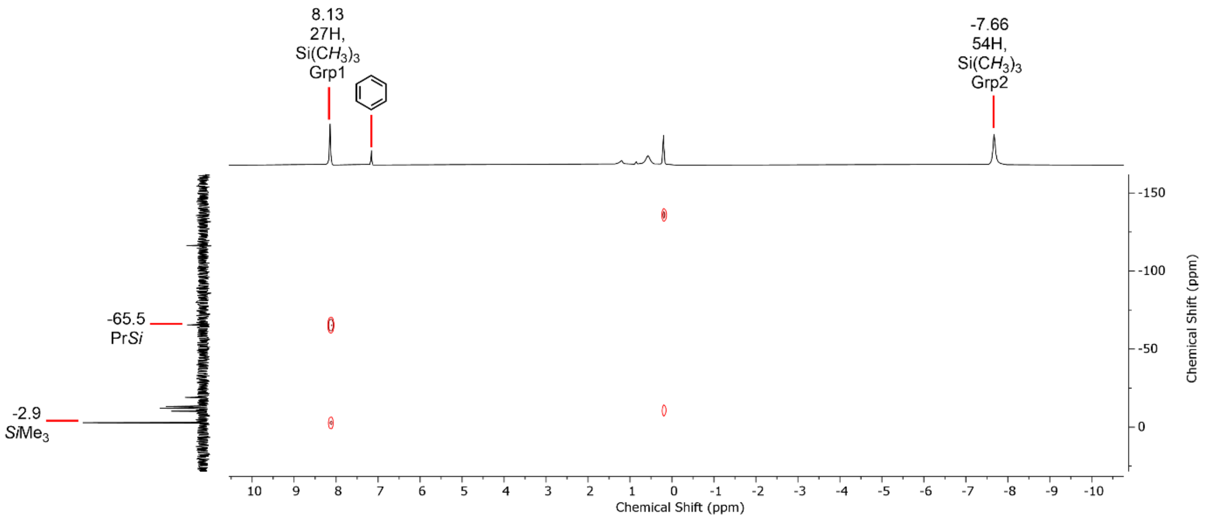

Figure S18. ^{1}H–^{29}Si HMBC NMR spectrum of **1-Pr** in C_6D_6/C_4D_8O (9 : 1 by volume).

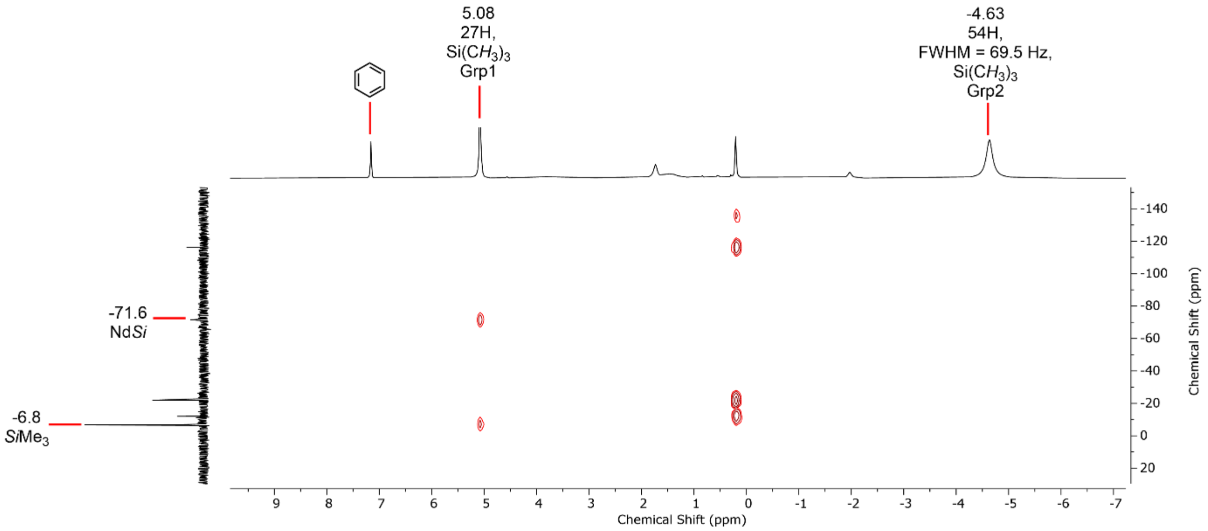

Figure S19. ^{1}H–^{29}Si HMBC NMR spectrum of **1-Nd** in C_6D_6/C_4D_8O (9 : 1 by volume).

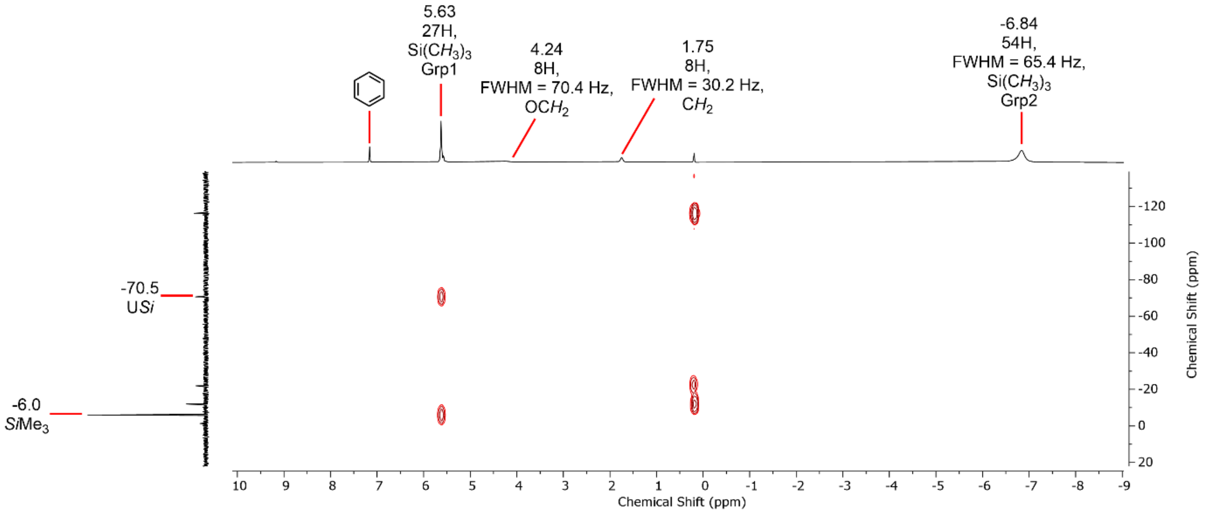

Figure S20. ^{1}H–^{29}Si HMBC NMR spectrum of **1-U** in C_6D_6/C_4D_8O (9 : 1 by volume).

1.5. Other 2D NMR Spectra of 1-M

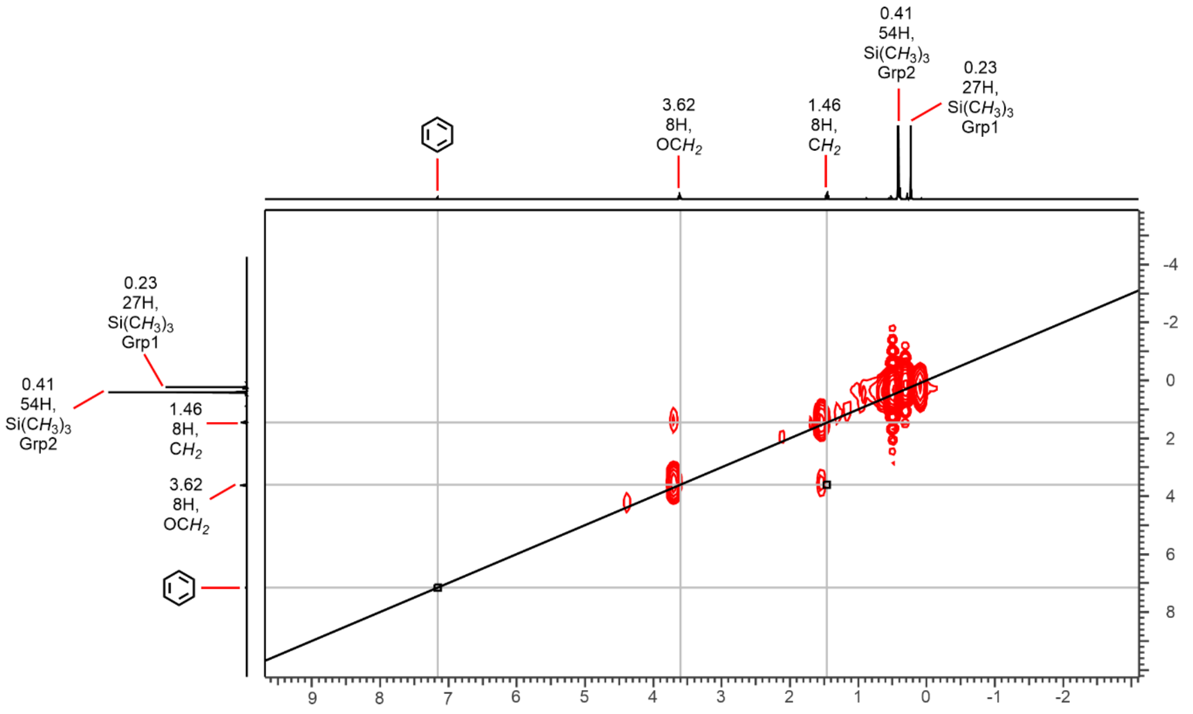

Figure S21. ^{1}H COSY NMR spectrum of **1-La** in C_6D_6/C_4D_8O (9 : 1 by volume).

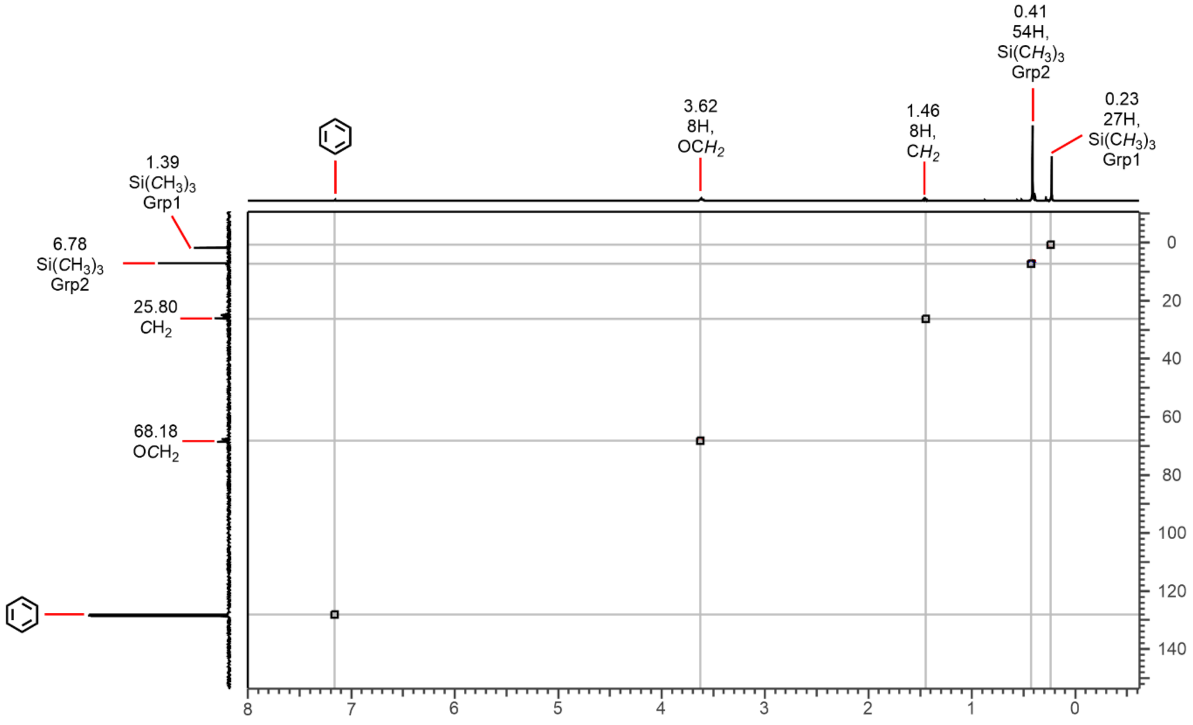

Figure S22. ^{1}H–^{13}C HSQC NMR spectrum of **1-La** in C_6D_6/C_4D_8O (9 : 1 by volume).

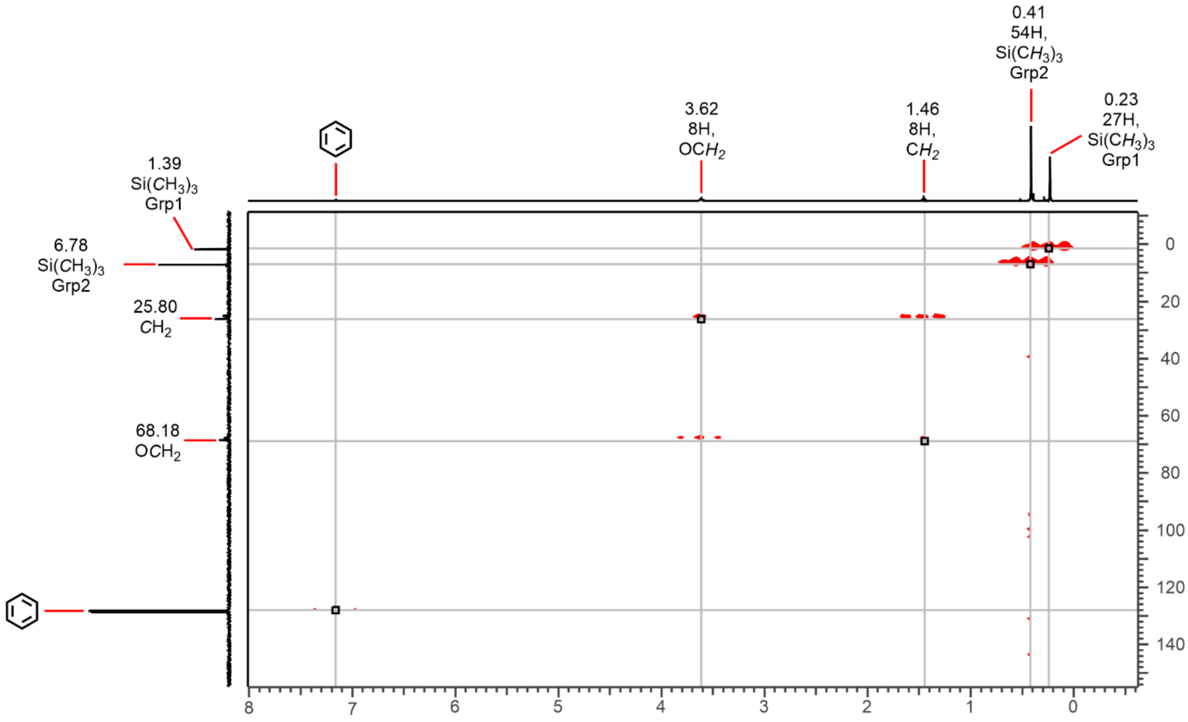

Figure S23. ^1H–^{13}C HMBC NMR spectrum of **1-La** in C_6D_6/C_4D_8O (9 : 1 by volume).

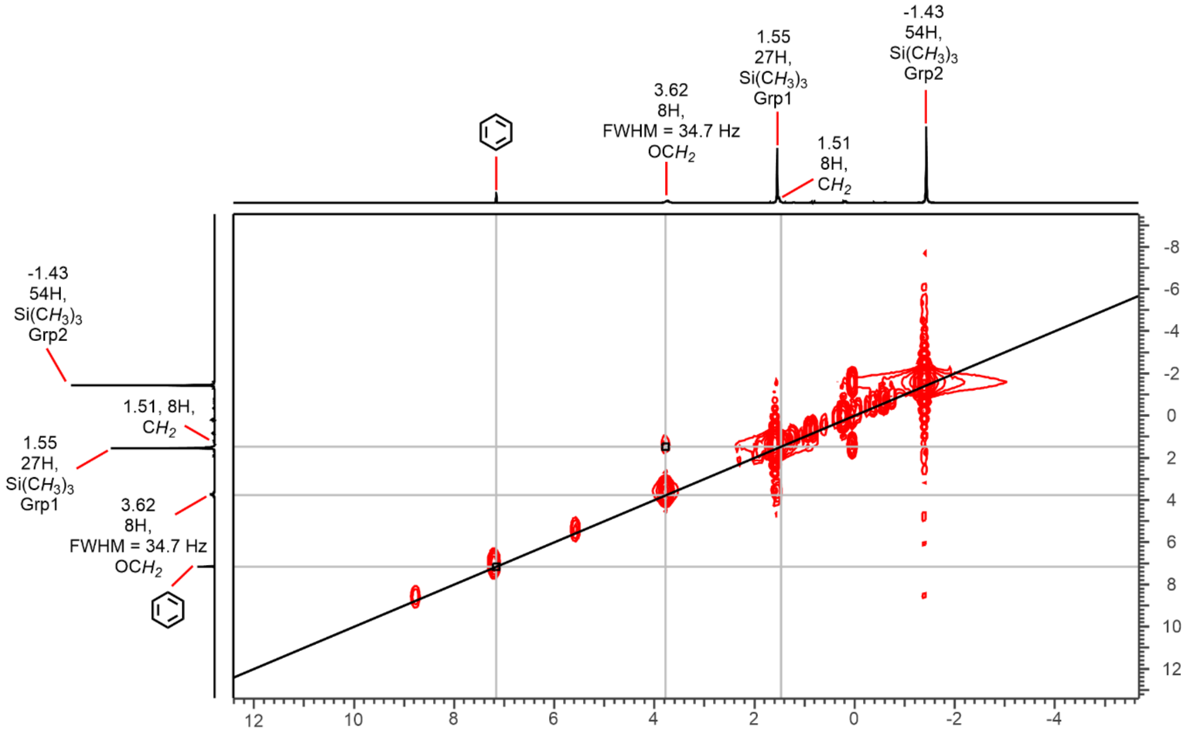

Figure S24. ^1H COSY NMR spectrum of **1-Ce** in C_6D_6/C_4D_8O (9 : 1 by volume).

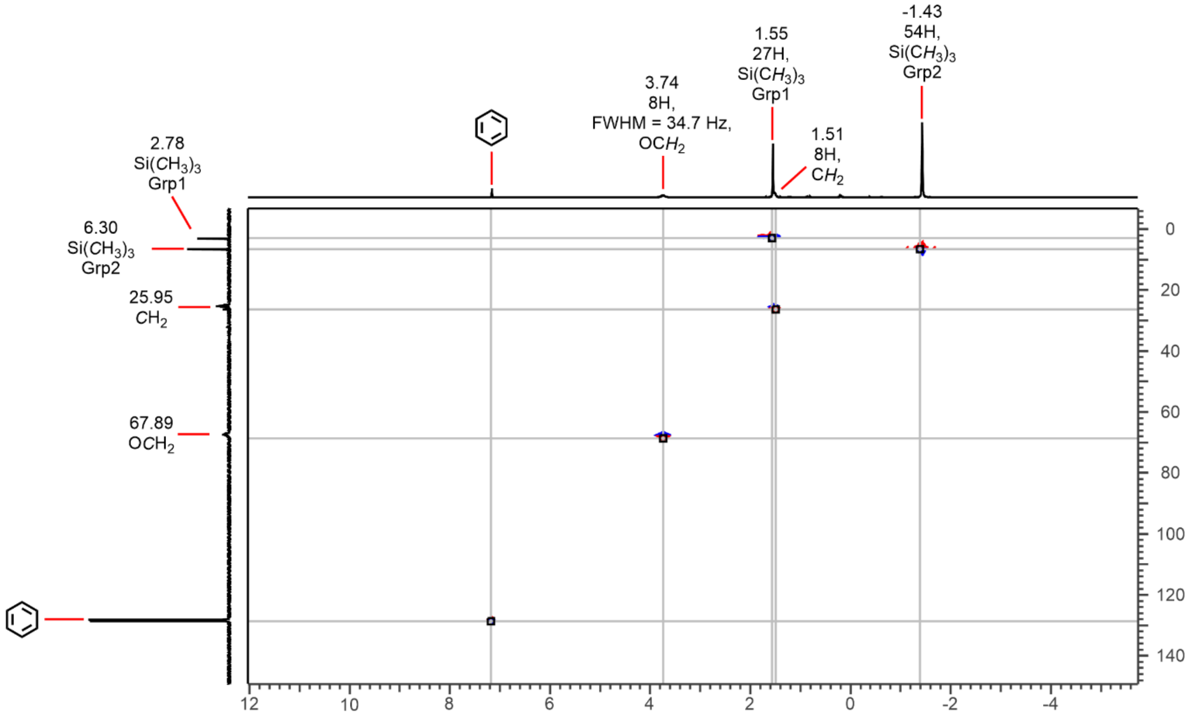

Figure S25. ^1H–^{13}C HSQC NMR spectrum of **1-Ce** in C_6D_6/C_4D_8O (9 : 1 by volume).

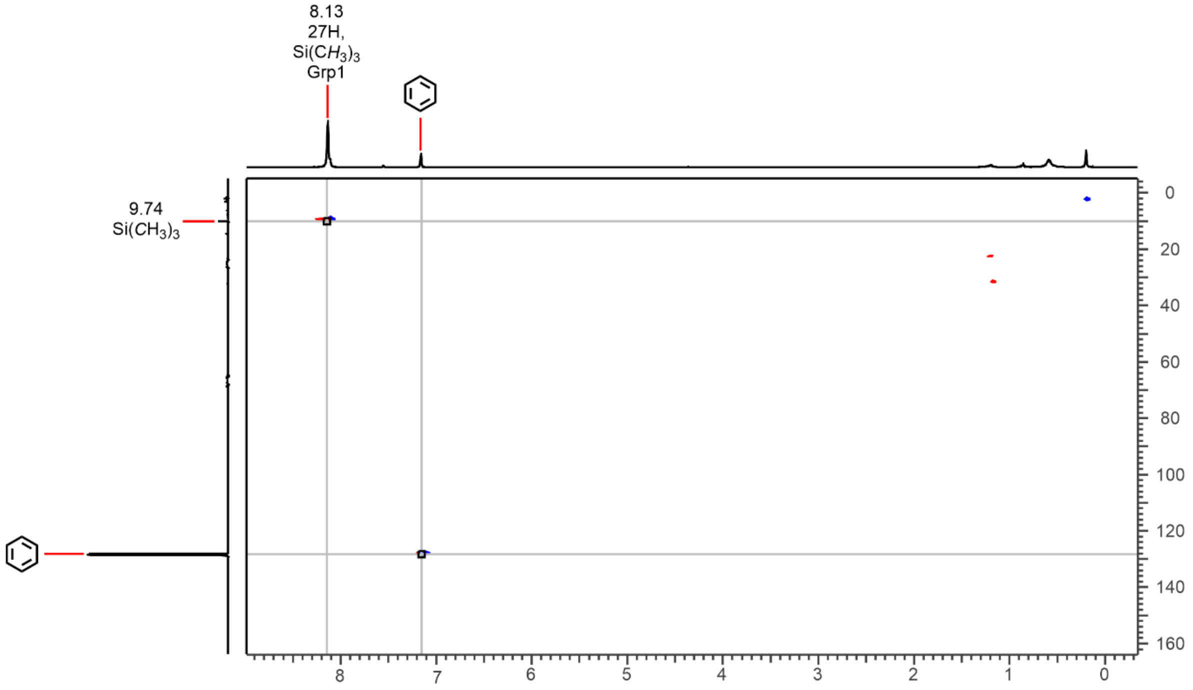

Figure S26. ^1H–^{13}C HSQC NMR spectrum of **1-Pr** in C_6D_6/C_4D_8O (9 : 1 by volume).

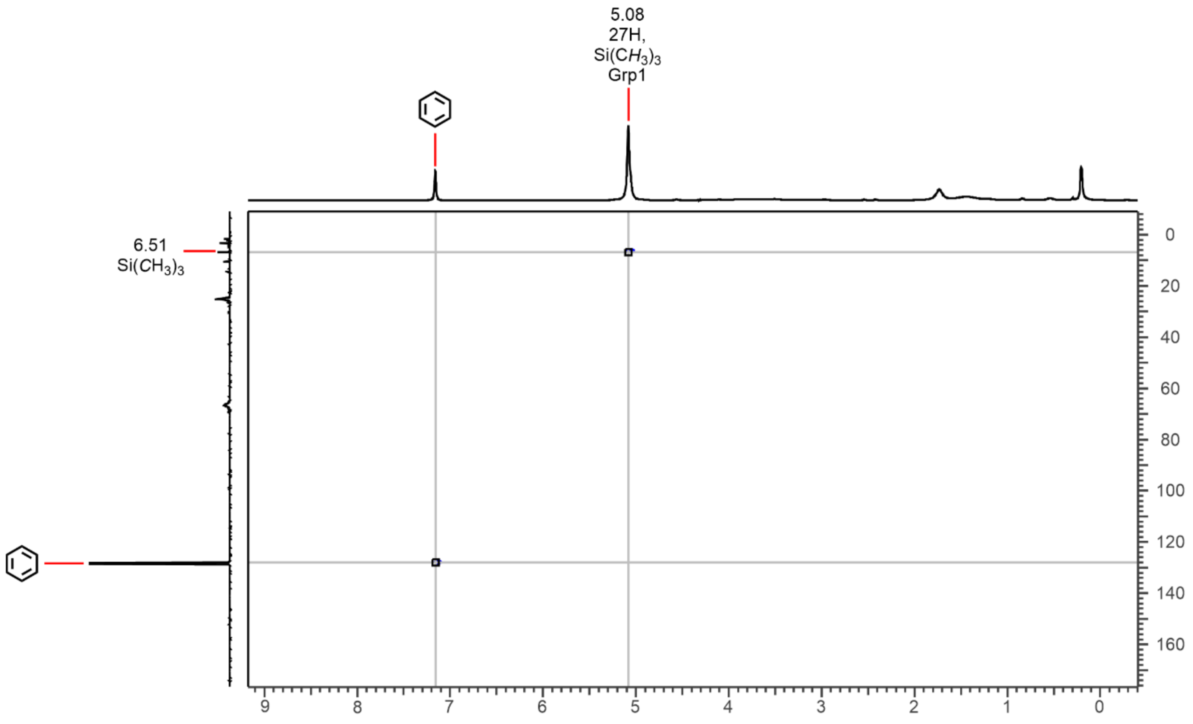

Figure S27. ^1H–^{13}C HSQC NMR spectrum of **1-Nd** in C_6D_6/C_4D_8O (9 : 1 by volume).

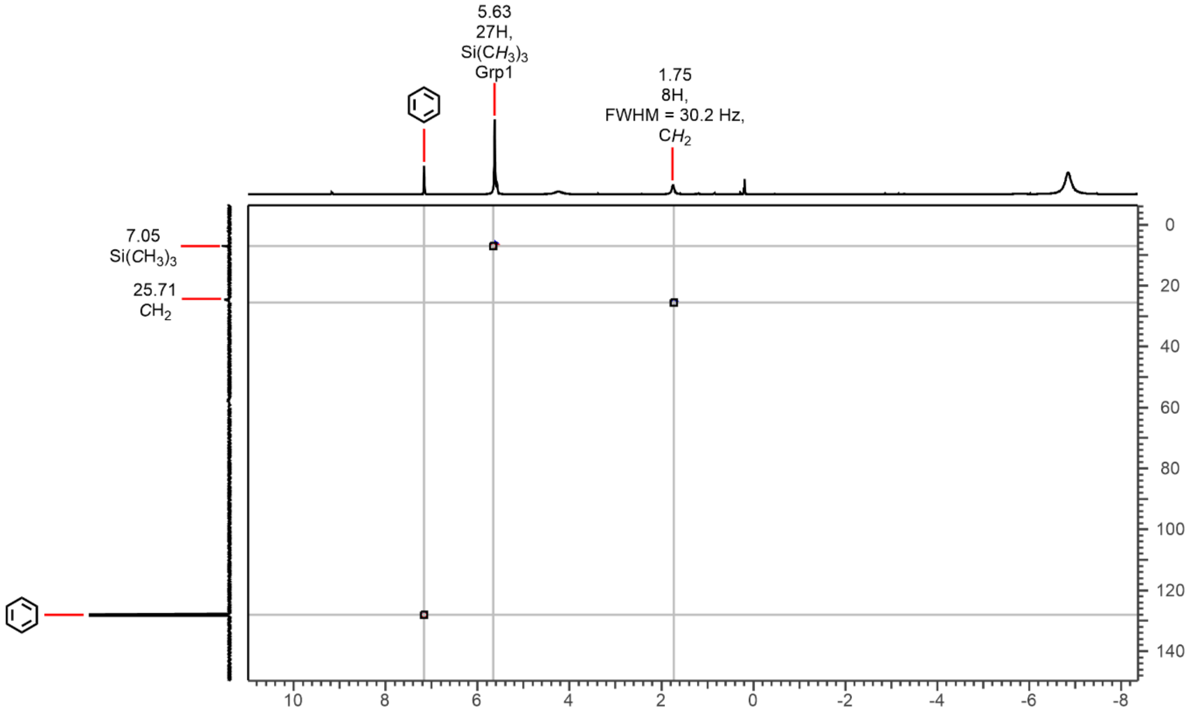

Figure S28. ^1H–^{13}C HSQC NMR spectrum of **1-U** in C_6D_6/C_4D_8O (9 : 1 by volume).

1.6. Solvent Stability

When preparing 9 : 1 C_6D_6/C_4D_8O solutions (by volume) of **1-M** for analysis by NMR spectroscopy the order in which C_6D_6 and C_4D_8O are added changes the rate of decomposition. This can be observed visually, as shown here for 50 mM solutions of **1-Pr** (Figure S29); when C_6D_6 is added first a dark orange solution forms, whereas when

C_4D_8O is added first an amber solution forms. The ^{1}H (Figure S30) and ^{29}Si DEPT90 (Figure S31) NMR spectra of these solutions collected within 2 hours of sample preparation also show different extents of decomposition; this is more prevalent in the latter spectra. Regardless of the order of addition all peaks corresponding to **1-M** can be identified by solution multinuclear NMR spectroscopy. The data reported herein are obtained from solutions where 10% C_4D_8O was added first followed by 90% C_6D_6, with NMR spectra collected within 2 hours of sample preparation, in order to minimize the extent to which decomposition products obscure NMR spectra.

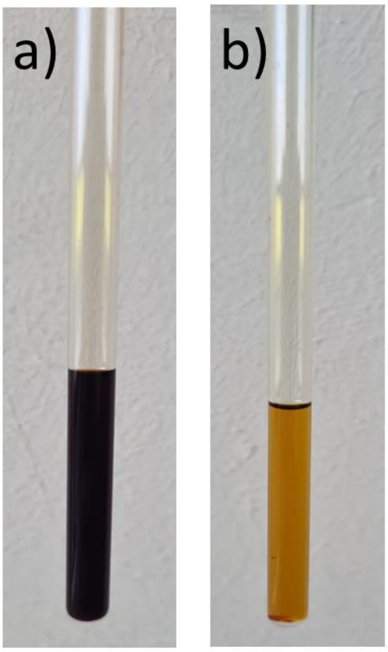

Figure S29. 50 mM samples of **1-Pr** in C_6D_6/C_4D_8O (9 : 1 by volume); a) Addition of 90% C_6D_6 to **1-Pr** followed by 10% C_4D_8O, b) Addition of 10% C_4D_8O to **1-Pr** followed by 90% C_6D_6.

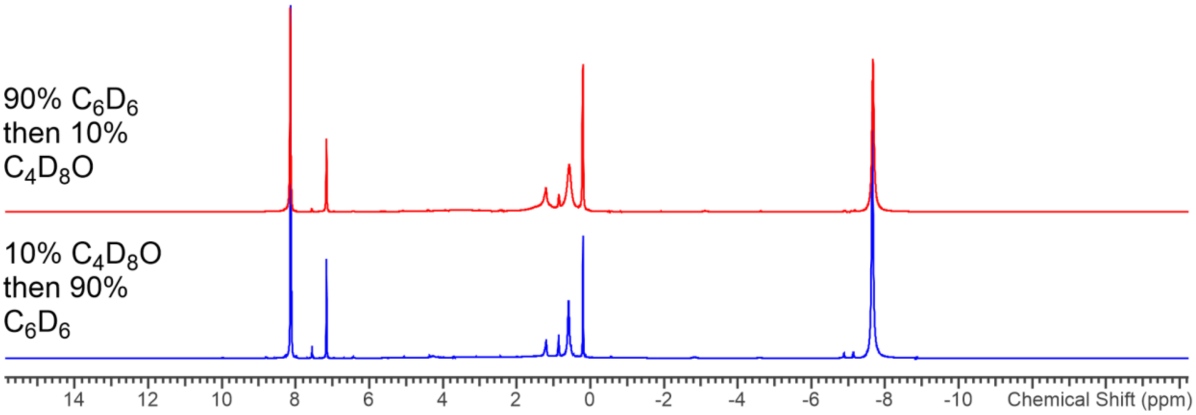

Figure S30. ^{1}H NMR spectrum (400.07 MHz) of **1-Pr** in C_6D_6/C_4D_8O (9 : 1 by volume); top – adding C_6D_6 followed by C_4D_8O, bottom – adding C_4D_8O followed by C_6D_6.

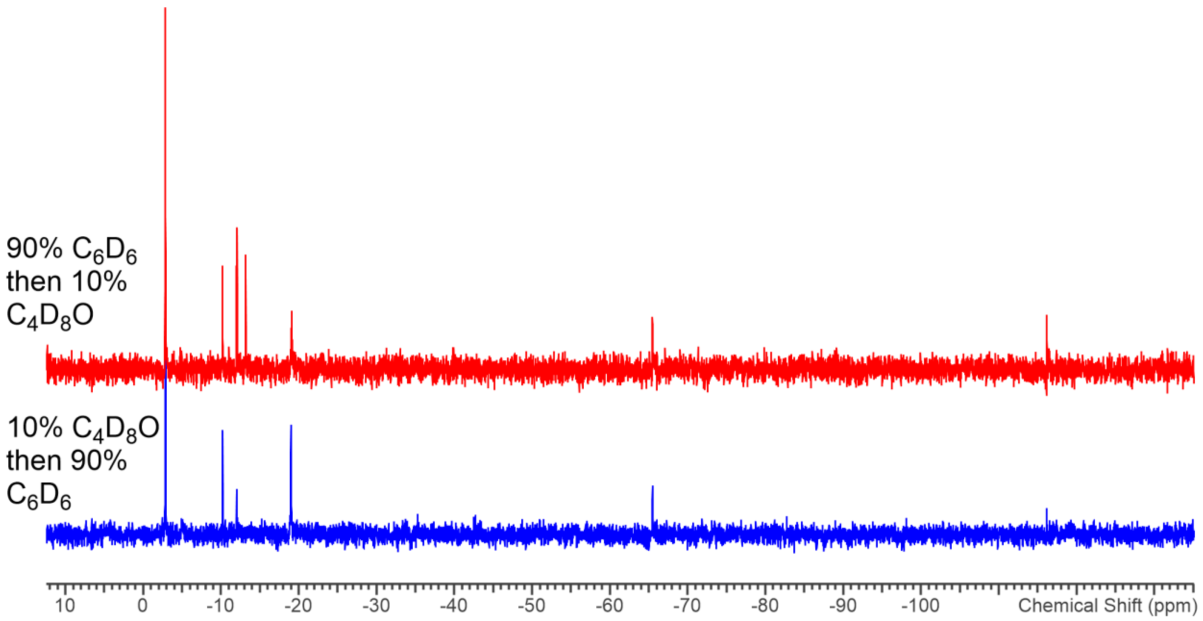

Figure S31. ^{29}Si DEPT90 NMR spectrum (79.48 MHz) of **1-Pr** in C_6D_6/C_4D_8O (9 : 1 by volume); top – adding C_6D_6 followed by C_4D_8O, bottom – adding C_4D_8O followed by C_6D_6.

1.7. Use of neat C_4D_8O *vs.* C_6D_6/C_4D_8O (9:1)

The previous experiment in section 1.6. indicates that the solution behavior of **1-M** is complex, and that decomposition pathways are accelerated by the loss of THF from **1-M** in solution. In an effort to reduce sample decomposition during collection of NMR spectra, and to investigate THF exchange dynamics further, a mixture of **1-La** (0.022 g, 0.021 mmol) and an internal standard (Mes*H, - 1,3,5-tri-*tert*-butylbenzene, 0.010 g, 0.042 mmol) was dissolved in neat C_4D_8O (0.540 g), and changes in the composition with time of the resultant pale yellow solution were monitored by ^{1}H, ^{2}H, ^{13}C{^{1}H} and ^{29}Si DEPT90 NMR spectroscopy, together with relevant 2D experiments. We assume that paramagnetic **1-M** should show similar sample decomposition and exchange dynamics to diamagnetic **1-La**.

The first NMR spectra of the **1-La**/Mes*H sample were completed within 2 hours of sample preparation. The ^{1}H NMR spectrum (Figure S32) contains two sets of THF signals, representing THF and C_4D_7HO; C_4D_8O is not observed by definition, and due to rapid exchange processes and an unknown amount of C_4D_7HO in C_4D_8O we cannot reliably integrate these signals nor quantify their relative ratio. Surprisingly, we observed three resonances in the region where $SiMe_3$ groups of **1-La** should occur; this contrasts with the two $SiMe_3$ signals seen for **1-La** in a 9 : 1 mixture by volume of C_6D_6/C_4D_8O, indicating that two or more species are present in the **1-La**/Mes*H sample. Both the ^{29}Si DEPT90 and ^{13}C{^{1}H} NMR spectra of the **1-La**/Mes*H sample after 2 hours also

showed multiple signals in the $SiMe_3$ region, and two resonances were seen in the metal-bound silicon region of the ^{29}Si DEPT90 NMR spectrum (δ_{Si} = –82.4 and –82.6 ppm), corroborating the presence of two La silanide environments (Figures S33 and S34). Analysis after 72 hours revealed that the signals in the $SiMe_3$ region of the ^{1}H, $^{13}C\{^{1}H\}$ and ^{29}Si DEPT NMR spectra shift, and only one broad metal-bound silicon resonance was observed in the ^{29}Si DEPT90 NMR spectrum (δ_{Si} = –82.6 ppm); we also note the growth of signals associated with $Si(SiMe_3)_4$. In addition, no differences were evident between ^{2}D NMR spectra collected within 2 hours or >72 hours after sample preparation, with only the expected two signals observed for C_4D_8O. Therefore, whilst decomposition of **1-La** is hindered when neat C_4D_8O is used as the NMR solvent, the resultant NMR spectra are not easier to interpret than the sample of **1-La** in a 9 : 1 mixture of C_6D_6/C_4D_8O by volume, and sample decomposition was not prevented, thus no firm conclusions were made.

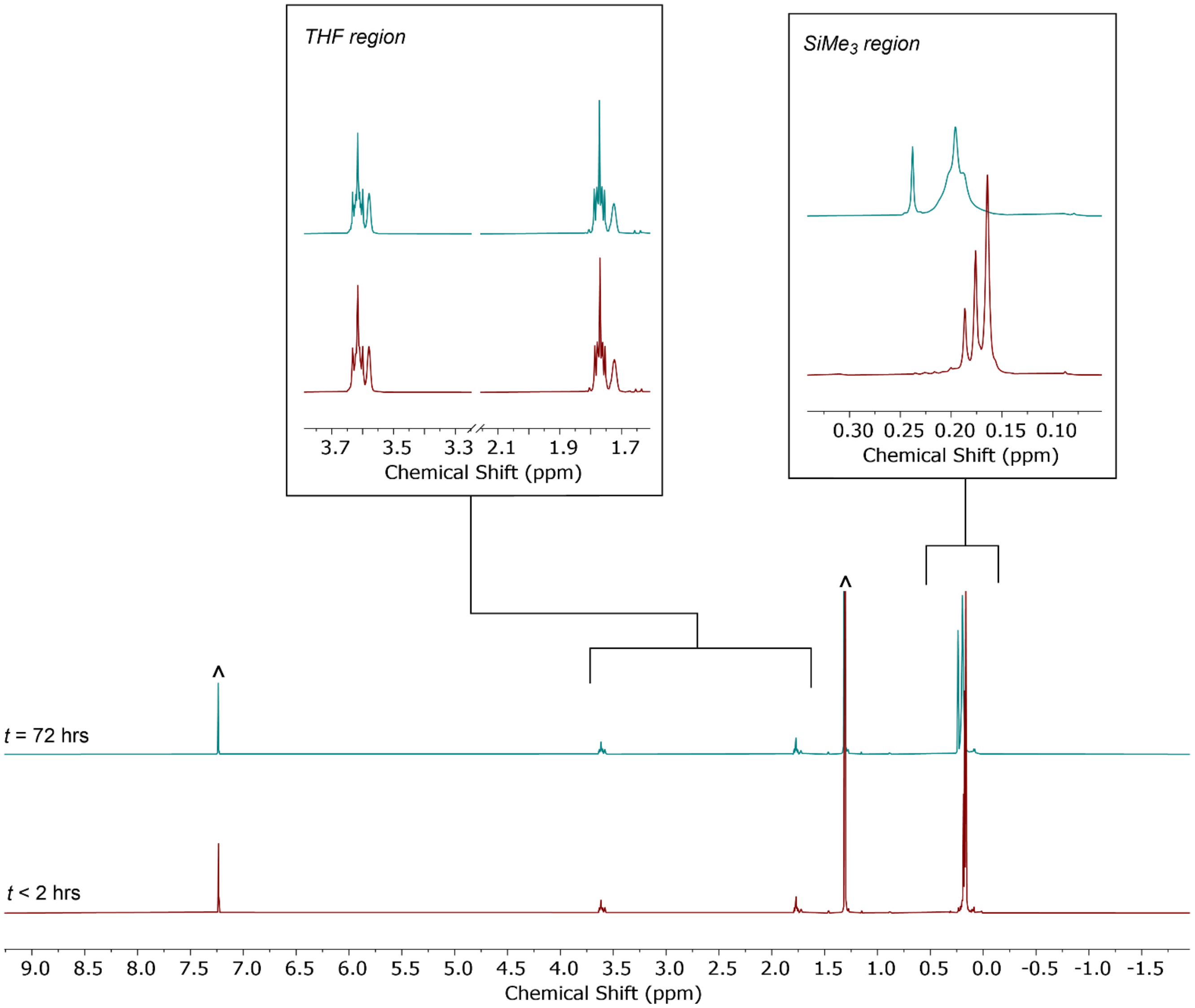

Figure S32. ^{1}H NMR spectrum (400.07 MHz) of **1-La** in C_4D_8O within two hours of sample preparation (red) and after 72 hours (turquoise). ^ denotes Mes*H internal standard.

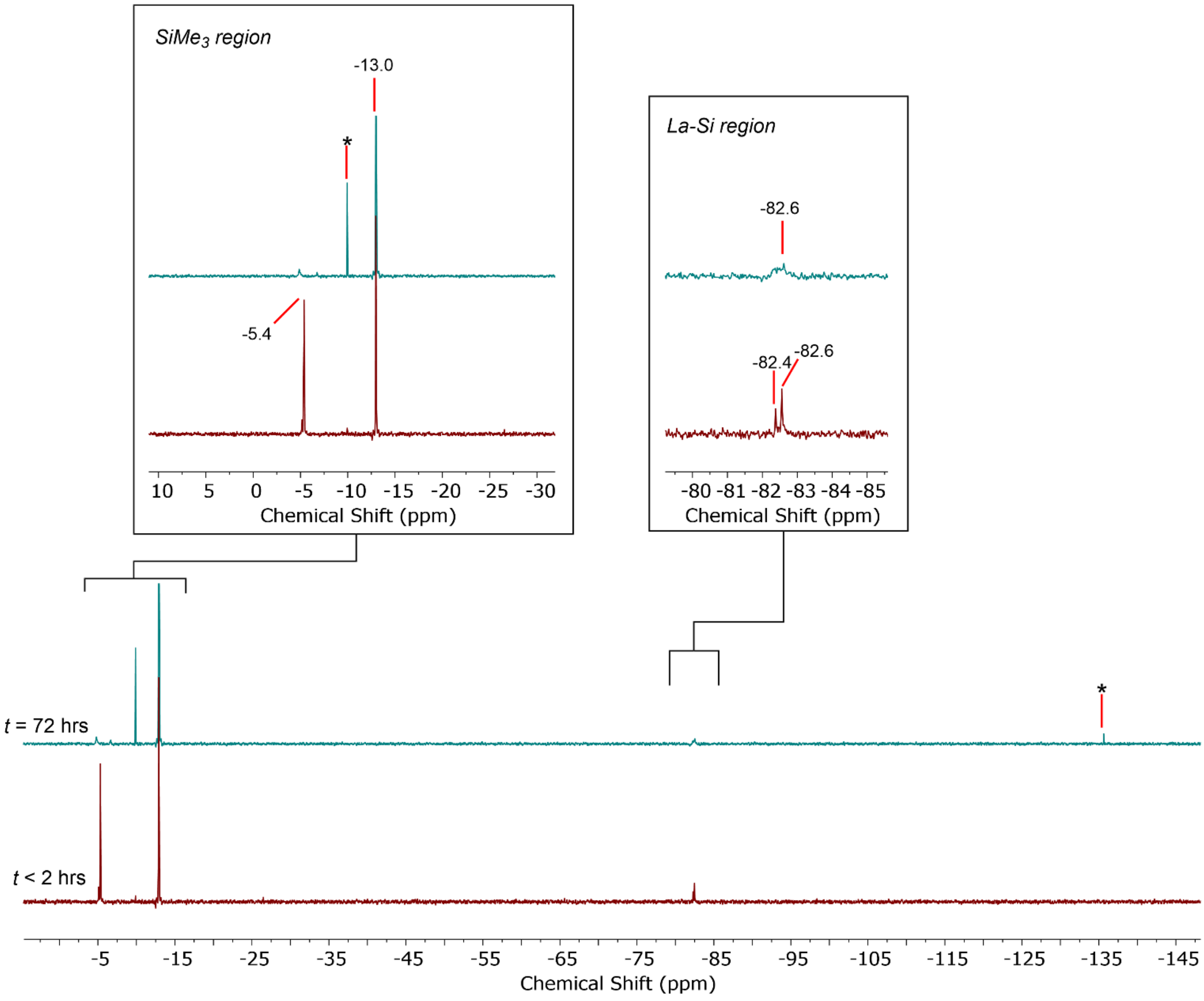

Figure S33. ^{29}Si DEPT90 NMR spectrum (79.48 MHz) of **1-La** in C_4D_8O within two hours of sample preparation (red) and after 72 hours (turquoise). * denotes $Si(SiMe_3)_4$ decomposition.

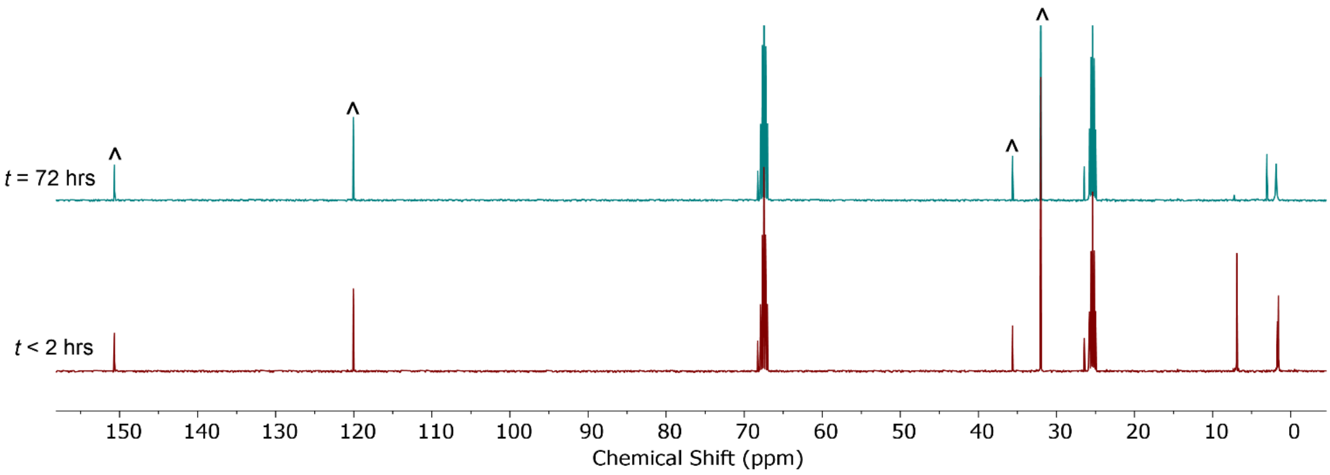

Figure S34. $^{13}C\{^1H\}$ NMR spectrum (125.77 MHz) of **1-La** in C_4D_8O within two hours of sample preparation (red) and after 72 hours (turquoise). ^ denotes Mes*H internal standard.

2. Solid-state NMR Spectroscopy

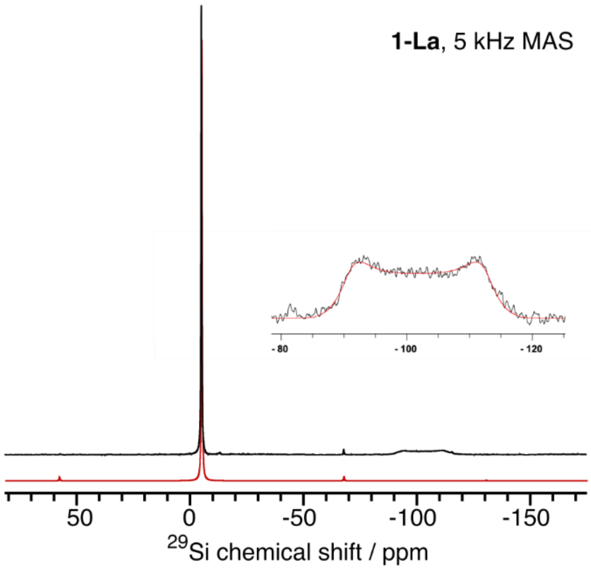

Figure S35. Solid-state {^{1}H-}^{29}Si CPMAS NMR spectrum of **1-La** recorded at ambient temperature with a MAS frequency = 5 kHz (black); simulated spectrum from *Si*Me_3 resonances (red) and the inset shows the simulated (WSolids1)[1] spectrum of the La*Si* resonances based on a La–Si *J* coupling of 290 Hz. 4096 transients were co-added with a repetition delay of 3 s.

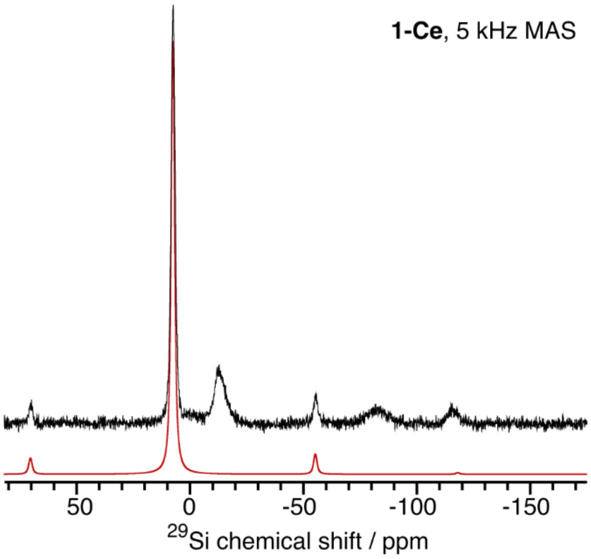

Figure S36. Solid-state {^{1}H-}^{29}Si CPMAS NMR spectrum of **1-Ce** recorded at ambient temperature with a MAS frequency = 5 kHz (black); simulated spectrum from *Si*Me_3 resonances (red). 4096 transients were co-added with a repetition delay of 0.03 s. The major diamagnetic impurity in the sample is assigned as $HSi(SiMe_3)_3$ (δ_{iso}\{^{29}Si\} (C_6D_6) = –115.6 ppm and –11.6 ppm); the signal at –82 ppm was not assigned.

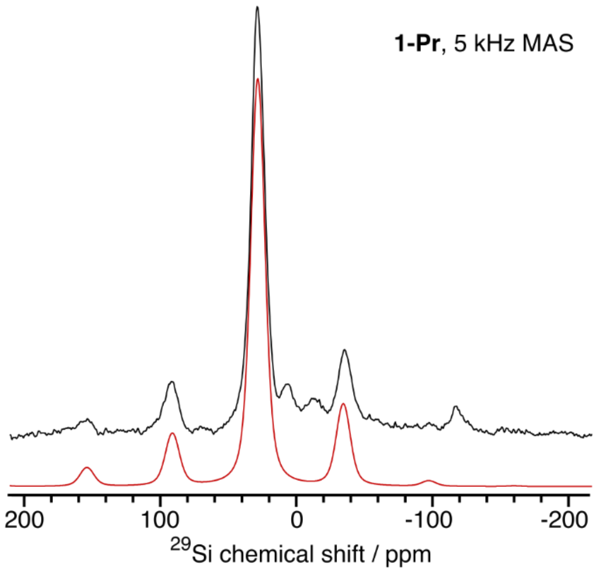

Figure S37. Solid-state {^{1}H-}^{29}Si CPMAS NMR spectrum of **1-Pr** recorded at ambient temperature with a MAS frequency = 5 kHz (black); simulated spectrum from *Si*Me_3 resonances (red). 189440 transients were co-added with a repetition delay of 0.03 s.

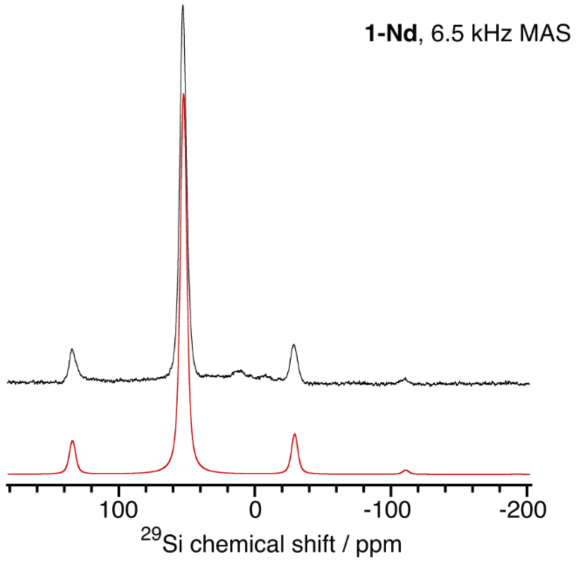

Figure S38. Solid-state ^{29}Si MAS NMR spectrum of **1-Nd** recorded at ambient temperature with a MAS frequency = 6.5 kHz (black); simulated spectrum from *Si*Me_3 resonances (red). 61440 transients were co-added with a repetition delay of 0.2 s.

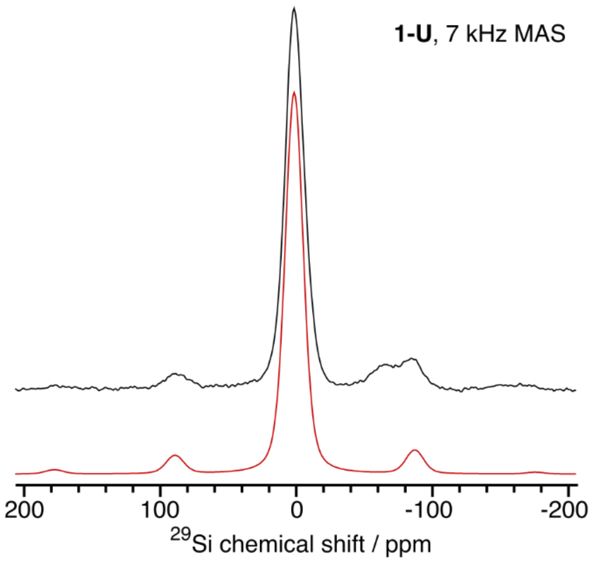

Figure S39. Solid-state ^{29}Si MAS NMR spectrum of **1-U** recorded at ambient temperature with a MAS frequency = 7 kHz (black); simulated spectrum from *Si*Me_3 resonances (red). 22796 transients were co-added with a repetition delay of 0.2 s.

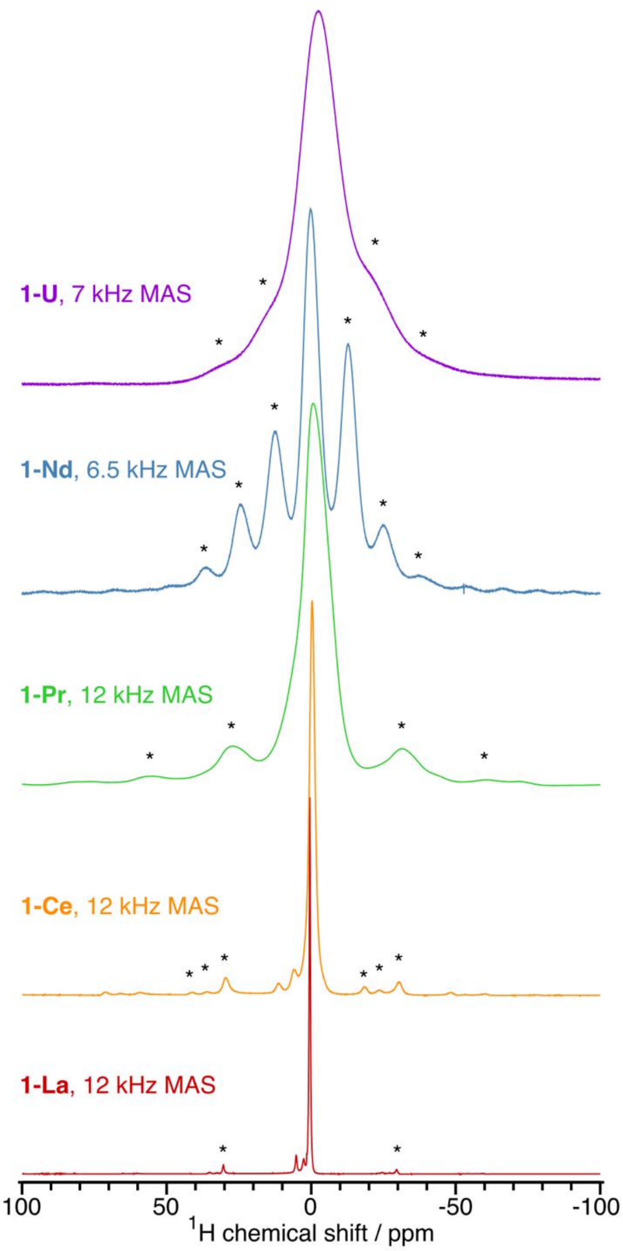

Figure S40. Solid-state ^{1}H MAS NMR spectra of **1-M** recorded at ambient temperature with the indicated MAS frequencies. Asterisks (*) denote spinning side bands. 16 to 128 transients were co-added for each, with the same repetition delays as used for the corresponding ^{29}Si MAS NMR spectra.

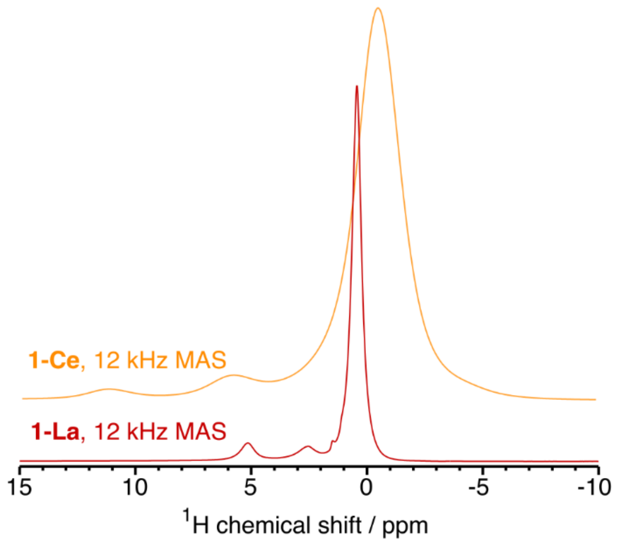

Figure S41. Zoom of the solid-state ^{1}H MAS NMR spectra of **1-La** and **1-Ce**, recorded at ambient temperature with the indicated MAS frequencies and 16 and 128 co-added transients, respectively, with the same repetition delays as used for the corresponding ^{29}Si MAS NMR spectra.

Table S2. Selected ^{1}H ssNMR parameters for THF in **1-La** and **1-Ce**. Note that these are not definitive due to large relative errors in the fitting.

	Species	^{1}H isotropic shift, δ_{iso} / ppm	^{1}H δ_{11} / ppm	^{1}H δ_{22} / ppm	^{1}H δ_{33} / ppm
**1-La**	THF α-CH_2	5.2	32.3	13.6	–30.3
	THF β-CH_2	2.6	22.9	16.1	–31.1
**1-Ce**	THF α-CH_2	11.3	94.1	–30.2	–30.2
	THF β-CH_2	5.9	55.0	–14.3	–23.1

3. ATR-IR Spectroscopy

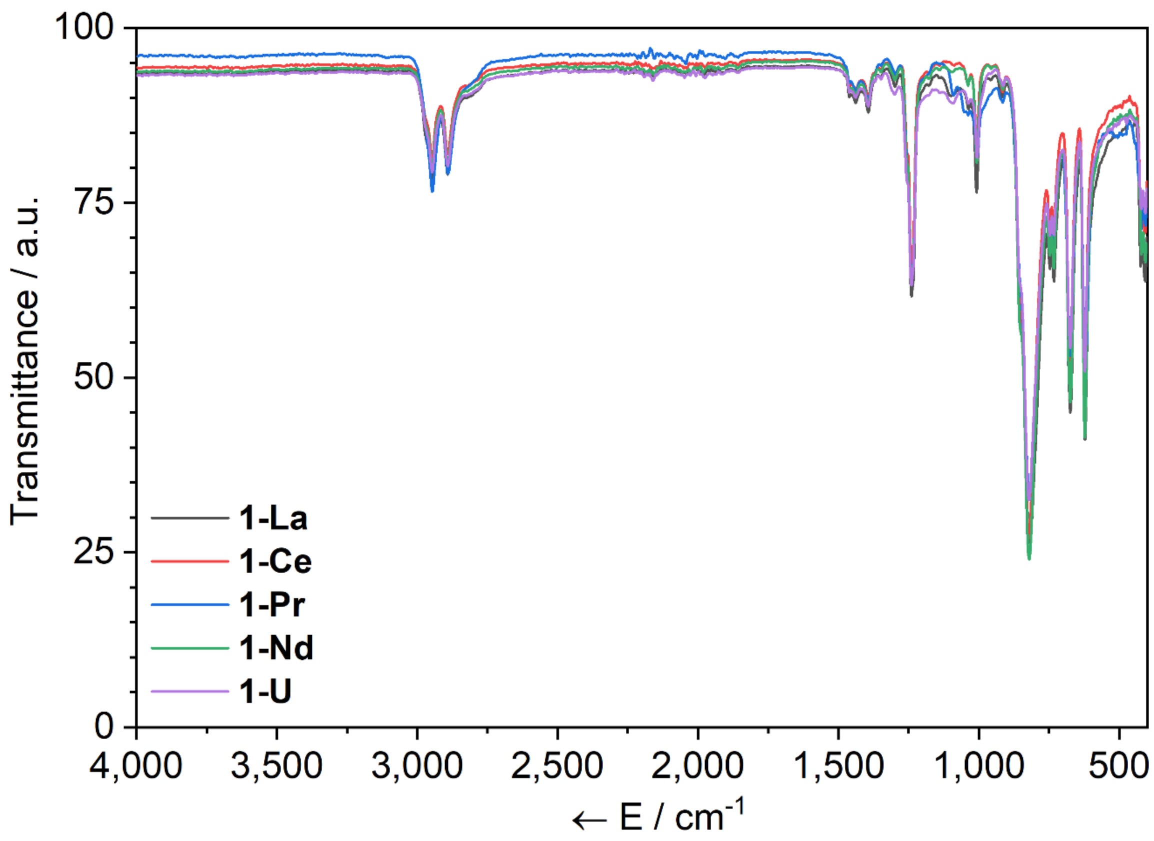

Figure S42. ATR-IR spectra of **1-M** between 398–4,000 cm^{-1}.

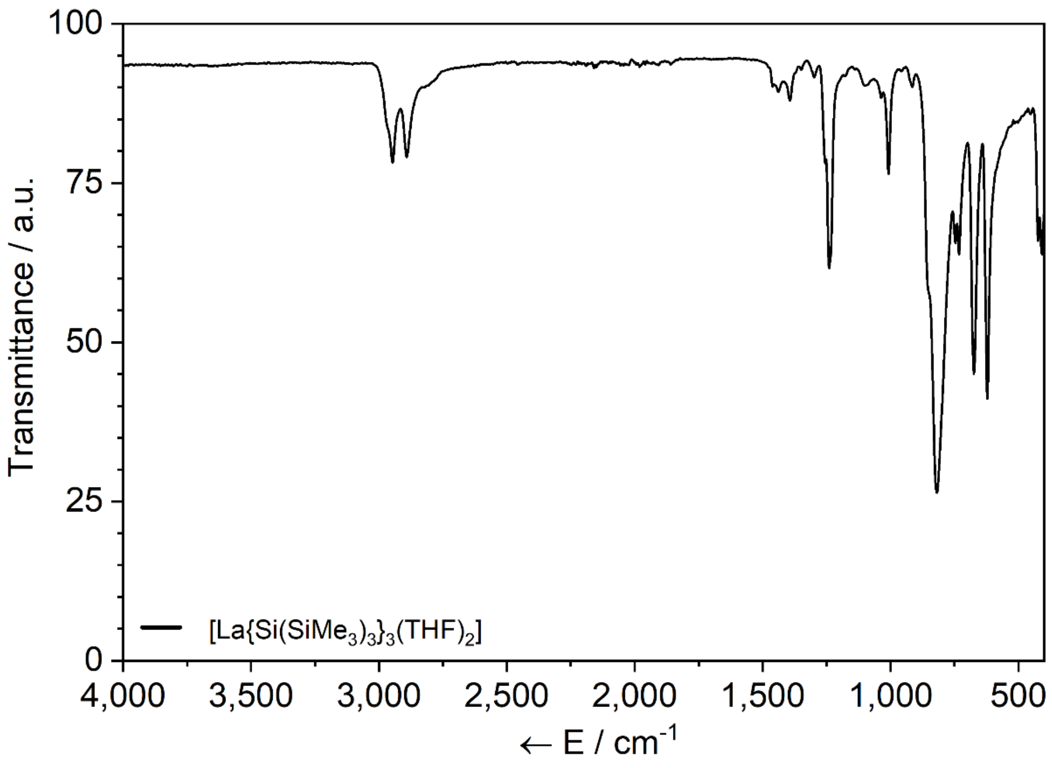

Figure S43. ATR-IR spectrum of **1-La** between 398–4,000 cm^{-1}.

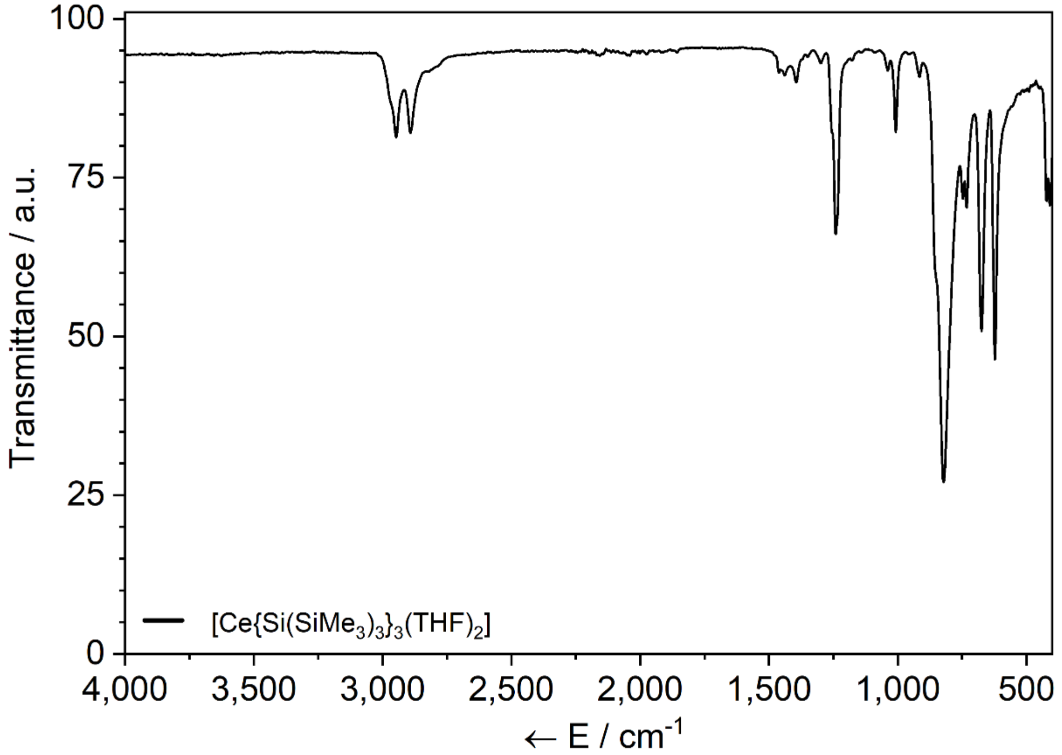

Figure S44. ATR-IR spectrum of **1-Ce** between 398–4,000 cm^{-1}.

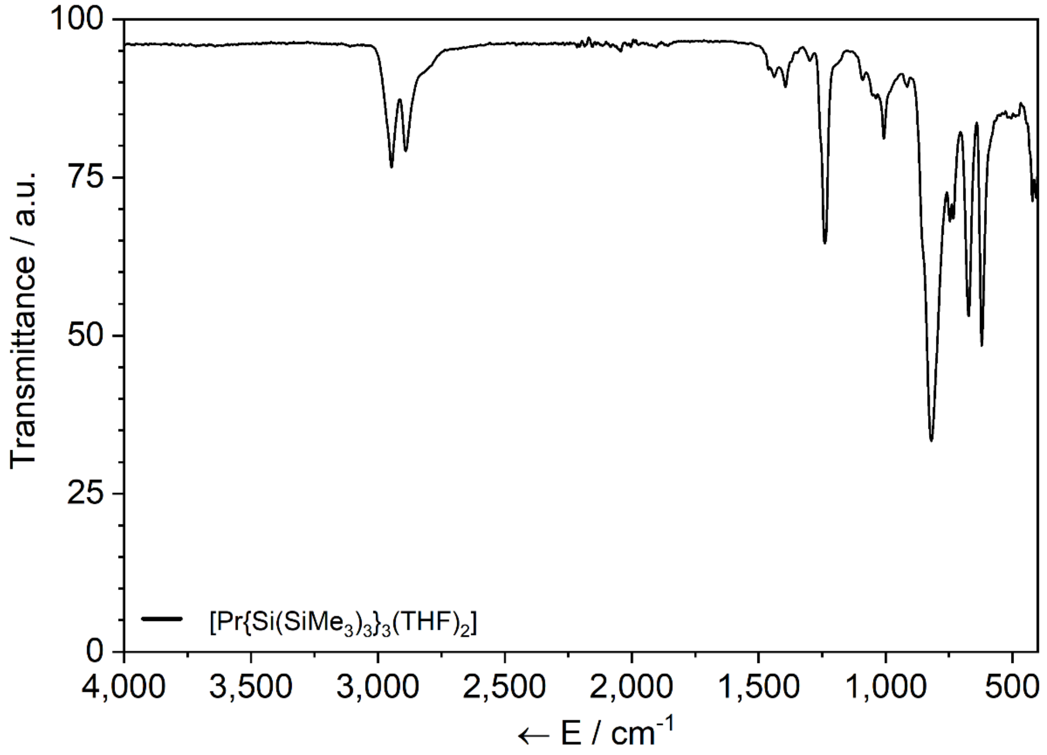

Figure S45. ATR-IR spectrum of **1-Pr** between 398–4,000 cm^{-1}.

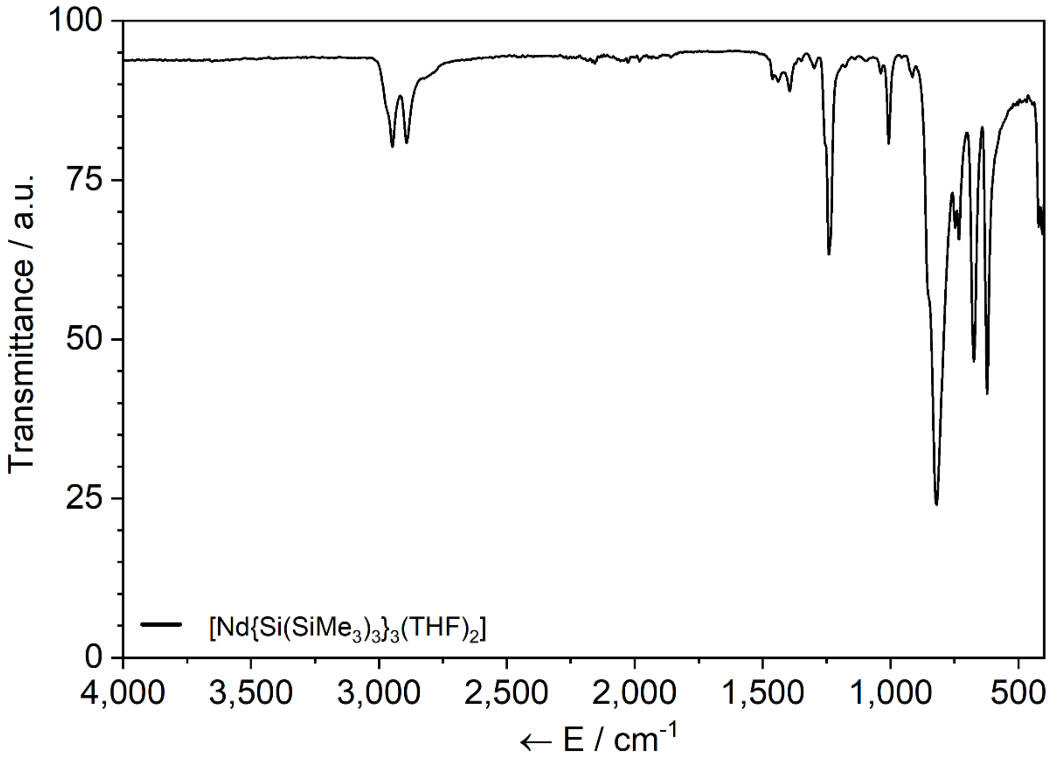

Figure S46. ATR-IR spectrum of **1-Nd** between 398–4,000 cm^{-1}.

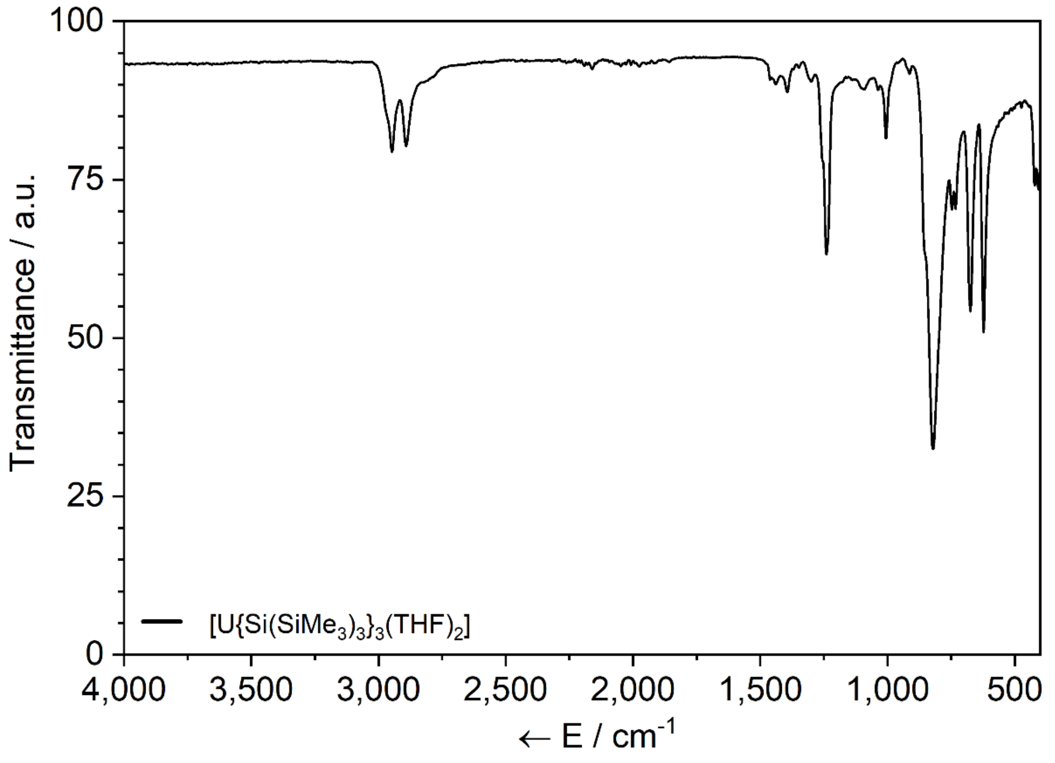

Figure S47. ATR-IR spectrum of **1-U** between 398–4,000 cm^{-1}.

4. UV-Vis-NIR Spectroscopy

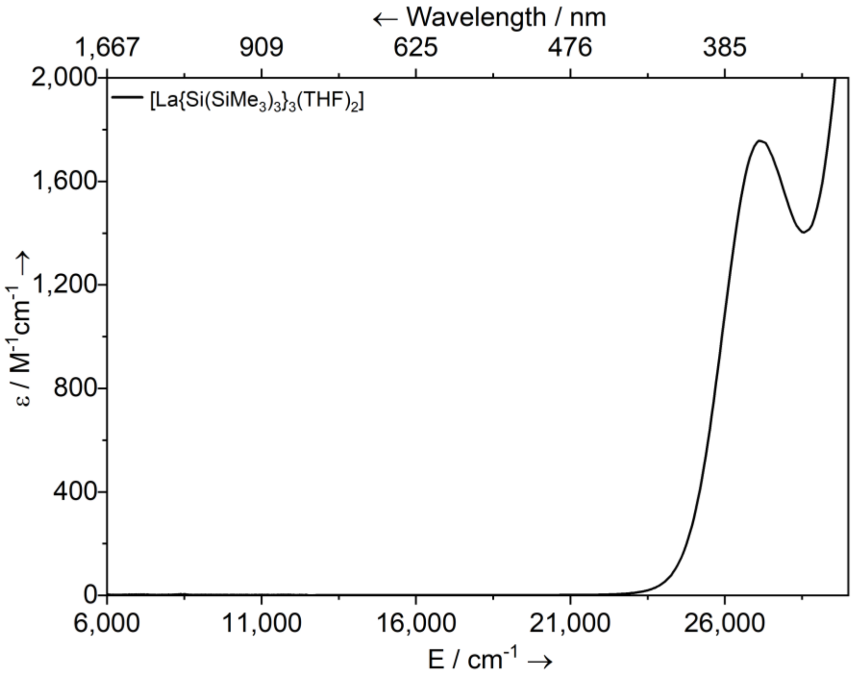

Figure S48. UV-Vis-NIR spectrum of **1-La** in THF (2 mM) between 6,000-30,000 cm^{-1} (1,667-333 nm).

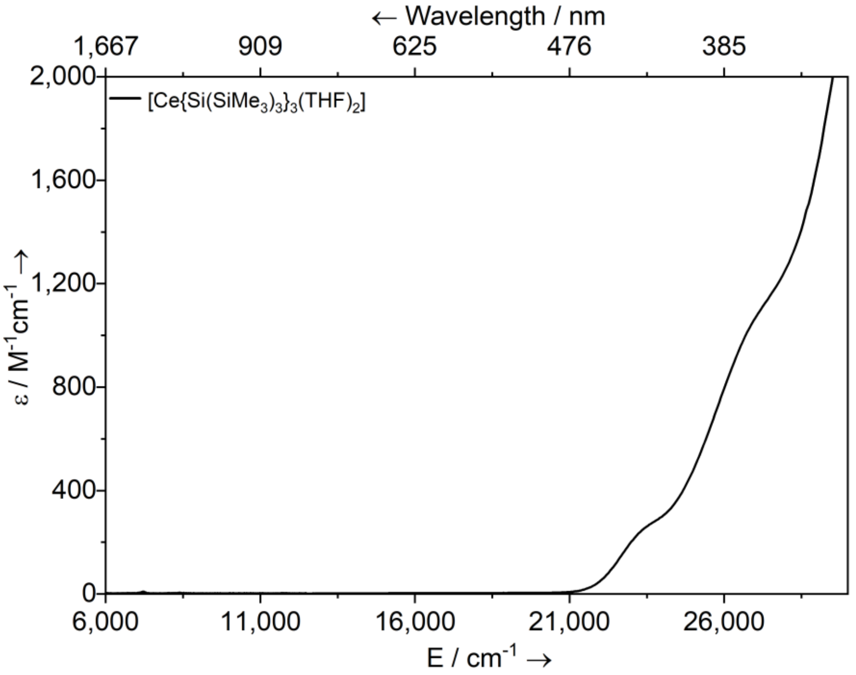

Figure S49. UV-Vis-NIR spectrum of **1-Ce** in THF (2 mM) between 6,000-30,000 cm^{-1} (1,667-333 nm).

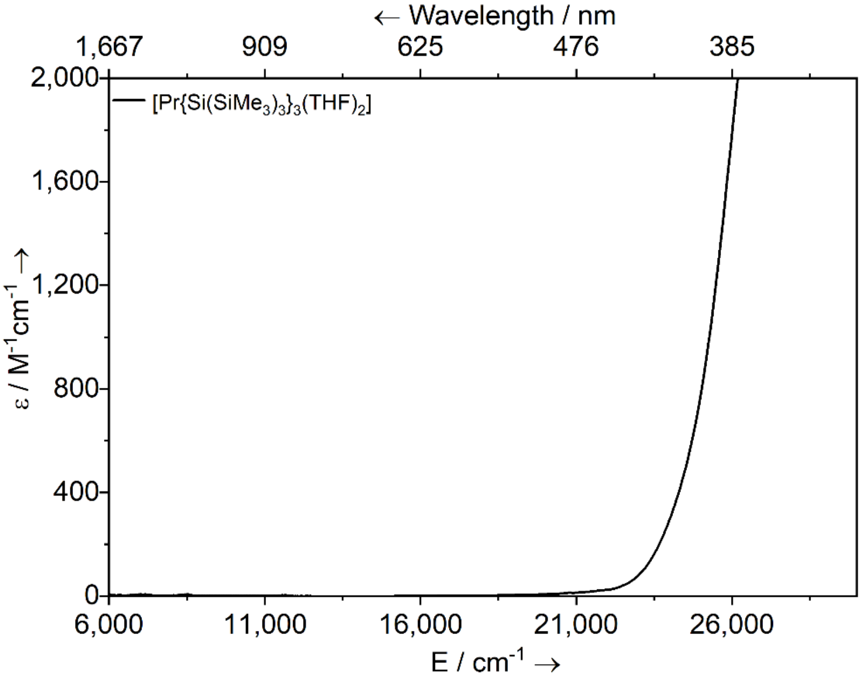

Figure S50. UV-Vis-NIR spectrum of **1-Pr** in THF (2 mM) between 6,000-30,000 cm^{-1} (1,667-333 nm).

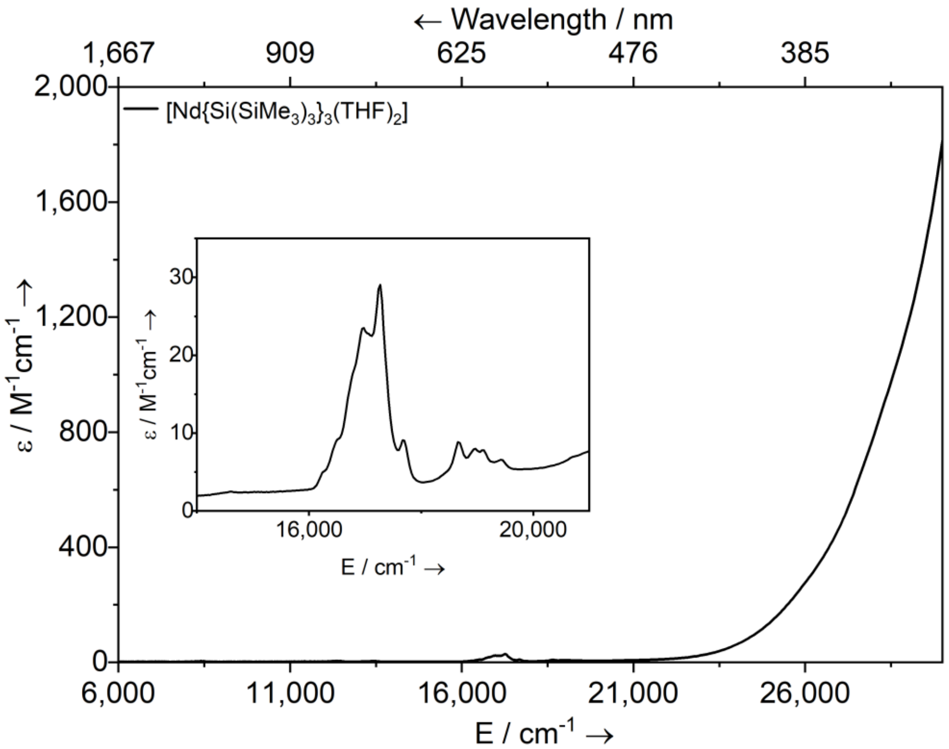

Figure S51. UV-Vis-NIR spectrum of **1-Nd** in THF (2 mM) between 6,000–30,000 cm^{-1} (1,667–333 nm), inset shows zoomed in region between 14,000–21,000 cm^{-1} (714–476 nm).

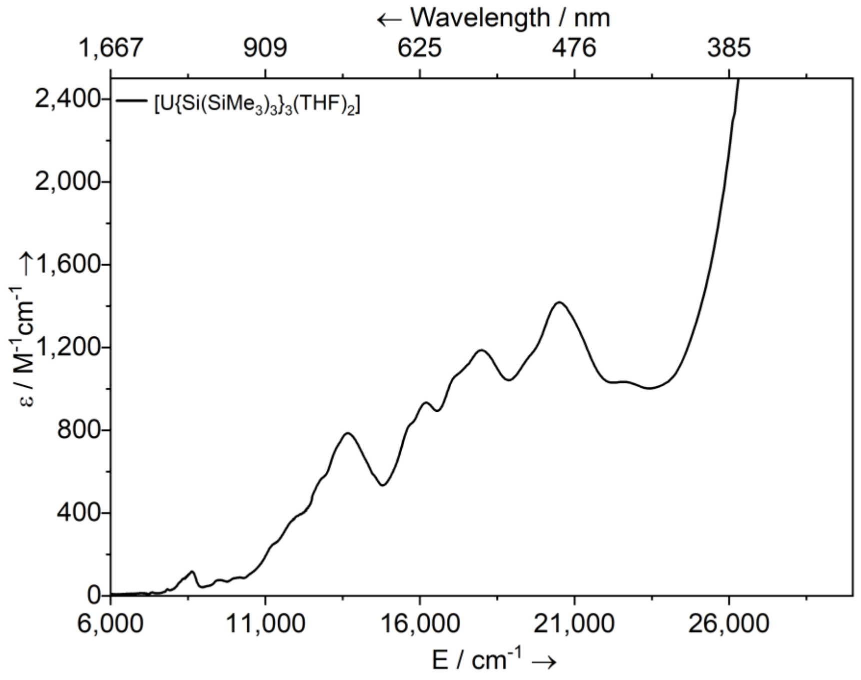

Figure S52. UV-Vis-NIR spectrum of **1-U** in THF (2 mM) between 6,000-30,000 cm^{-1} (1,667-333 nm).

5. Single Crystal X-ray Diffraction

Table S3. Crystallographic data for **1-M** (M = La, Ce, Pr).

	**1-La**	**1-Ce**	**1-Pr**	**1-Nd**	**1-U**
Formula	$C_{35}H_{97}LaO_2Si_{12}$	$C_{35}H_{97}CeO_2Si_{12}$	$C_{35}H_{97}PrO_2Si_{12}$	$C_{35}H_{97}NdO_2Si_{12}$	$C_{35}H_{97}UO_2Si_{12}$
Fw	1026.21	1027.30	1028.11	1031.42	1125.21
crystal size, mm	0.287×0.047×0.024	0.243×0.171×0.162	0.360×0.040×0.030	0.492×0.073×0.073	0.200×0.041×0.012
crystal color	Light yellow	Light orange	Orange	Light red	Dark green
crystal system	Hexagonal	Hexagonal	Hexagonal	Hexagonal	Hexagonal
space group	$P6_3/m$	$P6_3/m$	$P6_3$	$P6_3/m$	$P6_3/m$
collection temperature, K	100(2)	100(2)	150(2)	100(2)	150(2)
a, Å	14.1551(5)	14.13260(10)	14.1739(5)	14.10890(10)	14.1216(6)
b, Å	14.1551(5)	14.13260(10)	14.1739(5)	14.10890(10)	14.1216(6)
c, Å	16.9845(6)	16.94410(10)	16.9916(6)	16.9207(2)	16.9327(10)
α, °	-	-	-	-	-
β, °	-	-	-	-	-
γ, °	120	120	120	120	120
V, Å^3	2947.2(2)	2930.85(4)	29556.3(2)	2916.99(5)	2924.3(3)
Z	2	2	2	2	2
ρ_{calcd}, g cm^{-3}	1.156	1.164	1.155	1.174	1.278
μ, mm^{-1}	8.127	8.541	8.839	9.342	3.047
F(000)	1096	1098	1100	1102	1166
no. of reflections made	11677	16710	27017	23135	20327
no. of unique reflns, R_{int}	2099, 0.0988	2104, 0.0752	3589, 0.0933	1844, 0.0364	2537, 0.0523
no. of reflns $F^2 > 2\sigma(F^2)$	2024	2069	2835	1807	2083
transmn coeff range	0.204-0.829	0.231-0.338	0.143-0.777	0.091-0.549	0.581-0.964
R, R_w[a] ($F^2 > 2\sigma(F^2)$)	0.0957, 0.2159	0.0767, 0.2168	0.0565, 0.1437	0.0433, 0.1133	0.0392, 0.0904
R, R_w[a] (all data)	0.0978, 0.2170	0.0772, 0.2175	0.0773, 0.1573	0.0441, 0.1139	0.0548, 0.0961
S[a]	1.182	1.111	1.042	1.194	1.120
Parameters, Restraints	148, 321	126, 191	279, 675	163, 312	147, 242
max., min. diff map, e Å^{-3}	0.831, –1.819	1.006, –1.028	0.600, –1.110	0.523, –0.772	0.781, –0.922

[a] Conventional $R = \Sigma||F_o| - |F_c||/\Sigma|F_o|$; $R_w = [\Sigma w(F_o^2 - F_c^2)^2/\Sigma w(F_o^2)^2]^{1/2}$; $S = [\Sigma w(F_o^2 - F_c^2)^2/\text{no. data} - \text{no. params})]^{1/2}$ for all data.

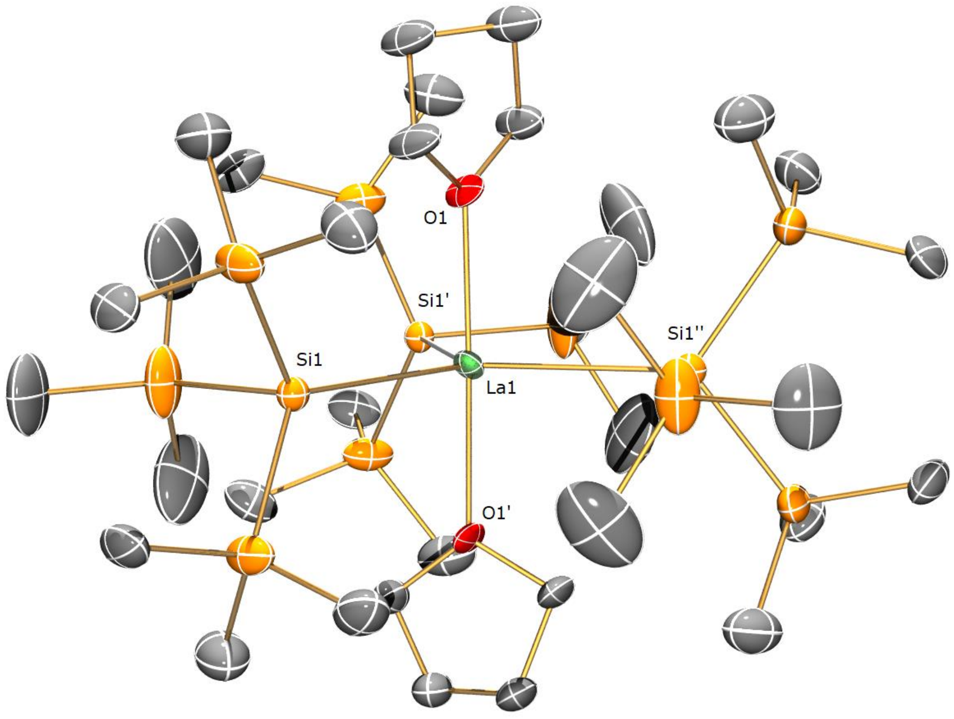

Figure S53. Molecular structure of **1-La** with selective atom labelling collected at 150 K. Displacement ellipsoids set at 30% probability level and hydrogen atoms removed for clarity.

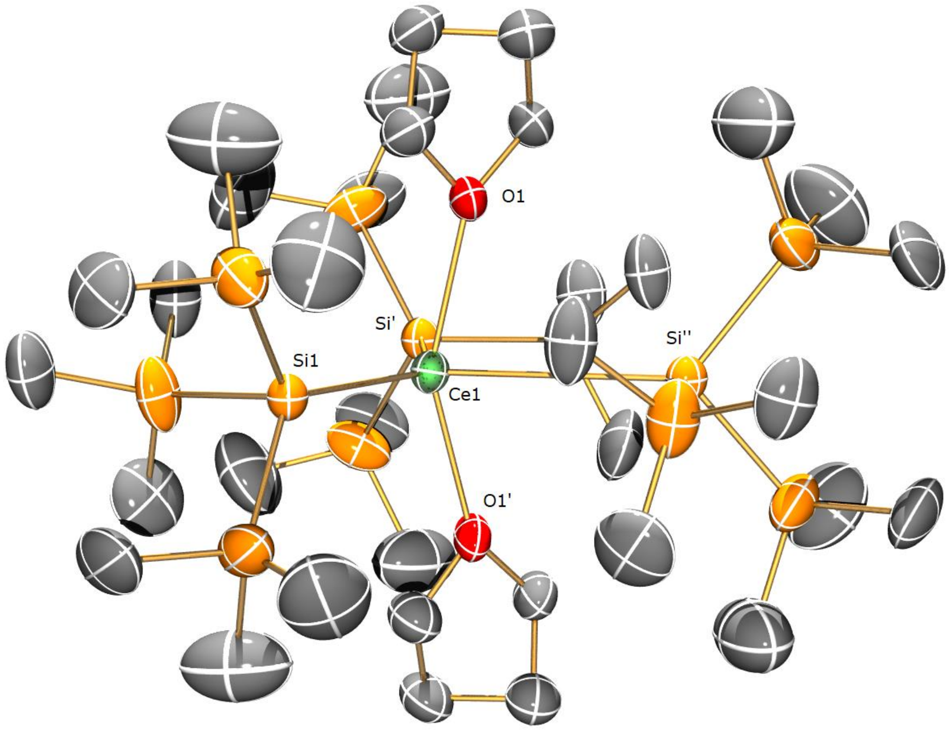

Figure S54. Molecular structure of **1-Ce** with selective atom labelling collected at 150 K. Displacement ellipsoids set at 30% probability level and hydrogen atoms removed for clarity.

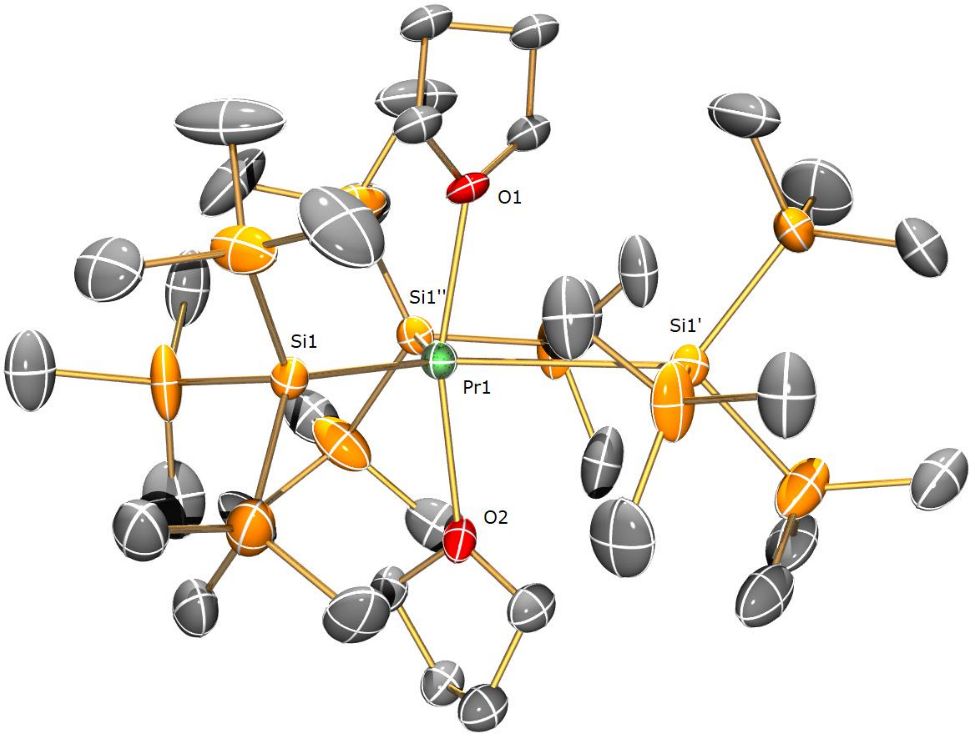

Figure S55. Molecular structure of **1-Pr** with selective atom labelling collected at 150 K. Displacement ellipsoids set at 30% probability level and hydrogen atoms removed for clarity.

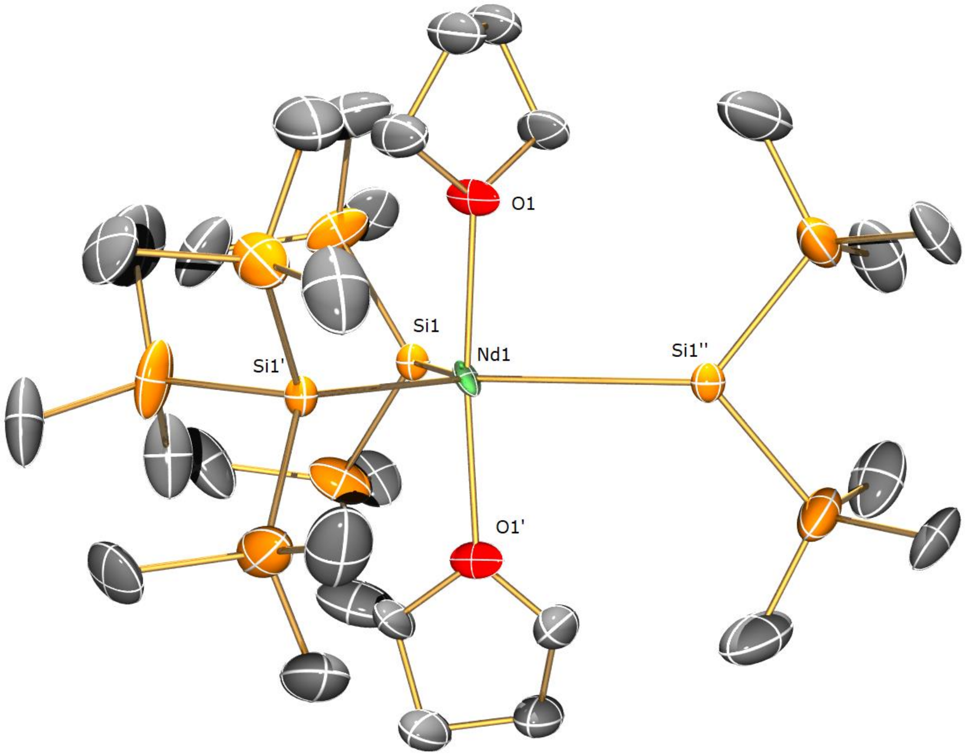

Figure S56. Molecular structure of **1-Nd** with selective atom labelling collected at 150 K. Displacement ellipsoids set at 30% probability level and hydrogen atoms removed for clarity.

6. Powder X-ray Diffraction

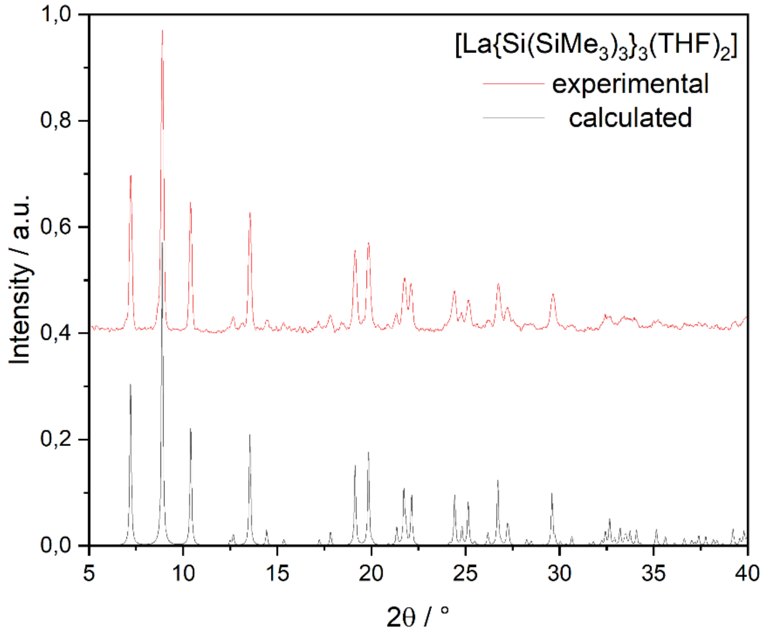

Figure S57. Powder X-ray diffraction pattern of **1-La** (red); theoretical powder X-ray diffraction pattern of **1-La** derived from crystallographic parameters (black).

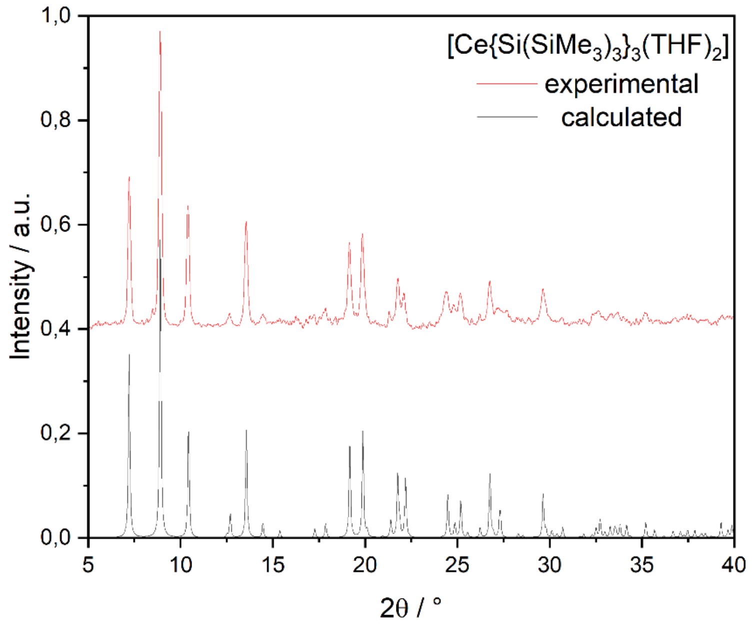

Figure S58. Powder X-ray diffraction pattern of **1-Ce** (red); theoretical powder X-ray diffraction pattern of **1-Ce** derived from crystallographic parameters (black).

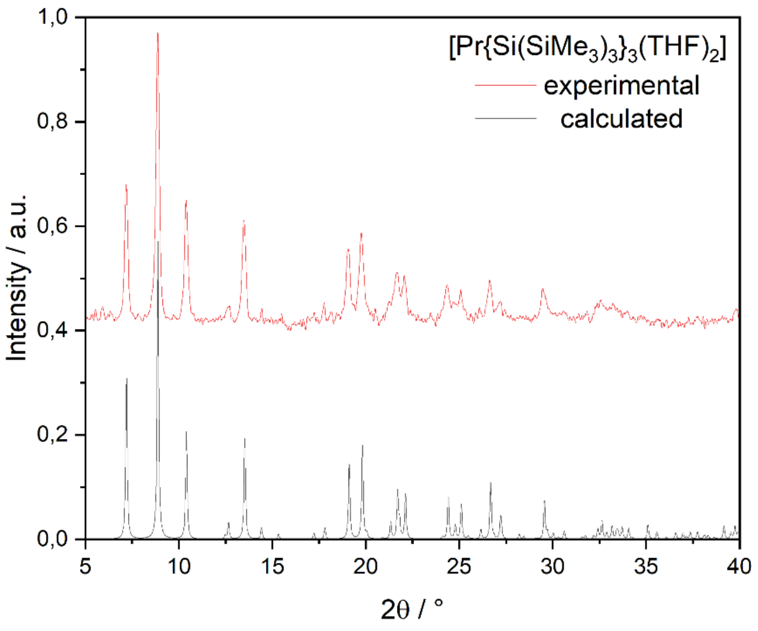

Figure S59. Powder X-ray diffraction pattern of **1-Pr** (red); theoretical powder X-ray diffraction pattern of **1-Pr** derived from crystallographic parameters (black).

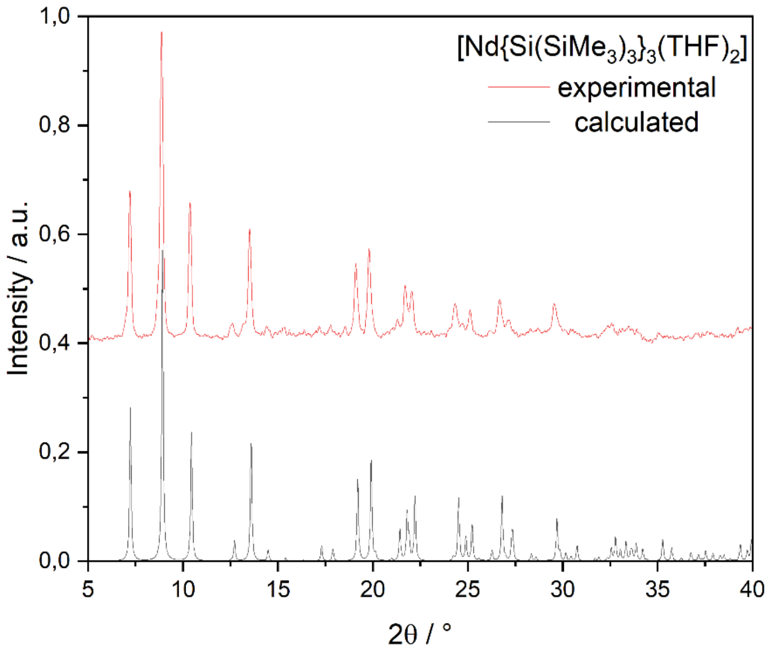

Figure S60. Powder X-ray diffraction pattern of **1-Nd** (red); theoretical powder X-ray diffraction pattern of **1-Nd** derived from crystallographic parameters (black).

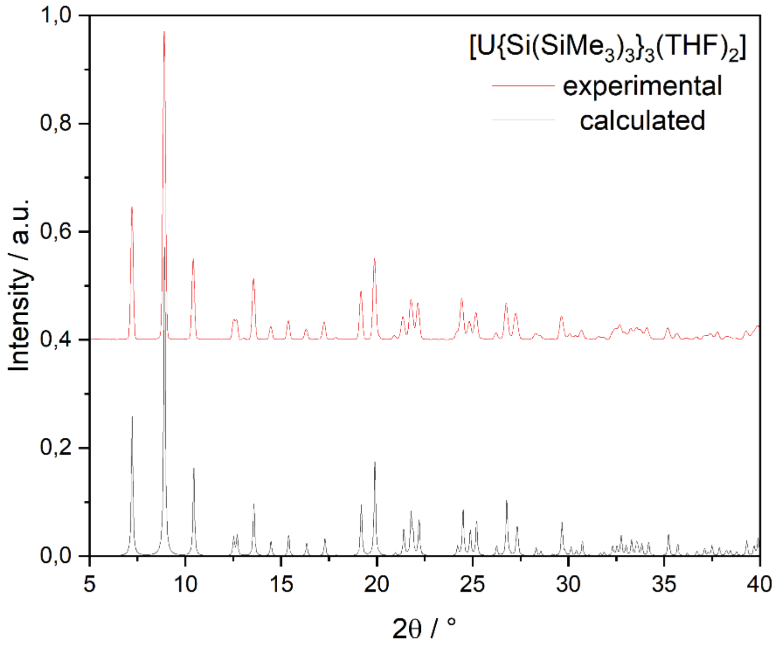

Figure S61. Powder X-ray diffraction pattern of **1-U** (red); theoretical powder X-ray diffraction pattern of **1-U** derived from crystallographic parameters (black).

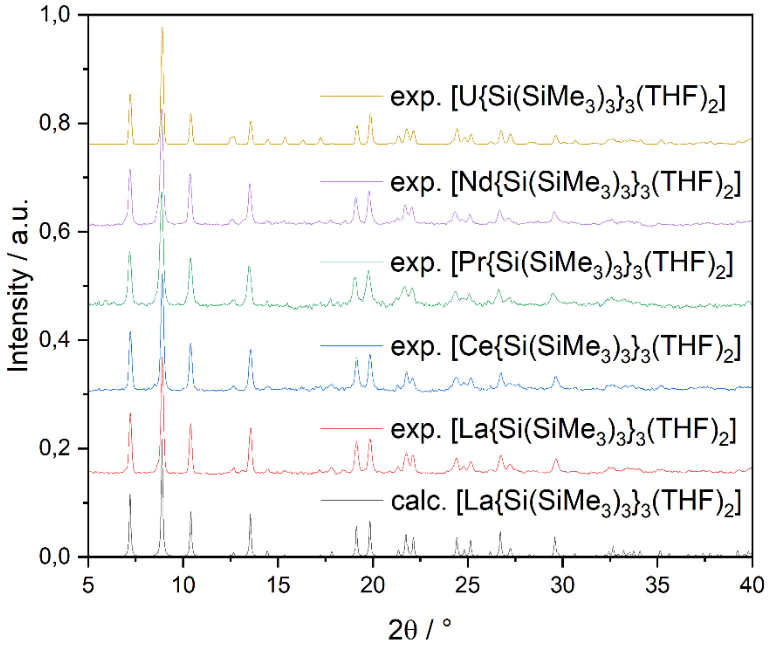

Figure S62. Overlaid powder X-ray diffraction patterns of **1-La** (red), **1-Ce** (blue), **1-Pr** (green), **1-Nd** (purple), and **1-U** (orange); theoretical powder X-ray diffraction pattern of **1-La** derived from crystallographic parameters (black).

a)
b)

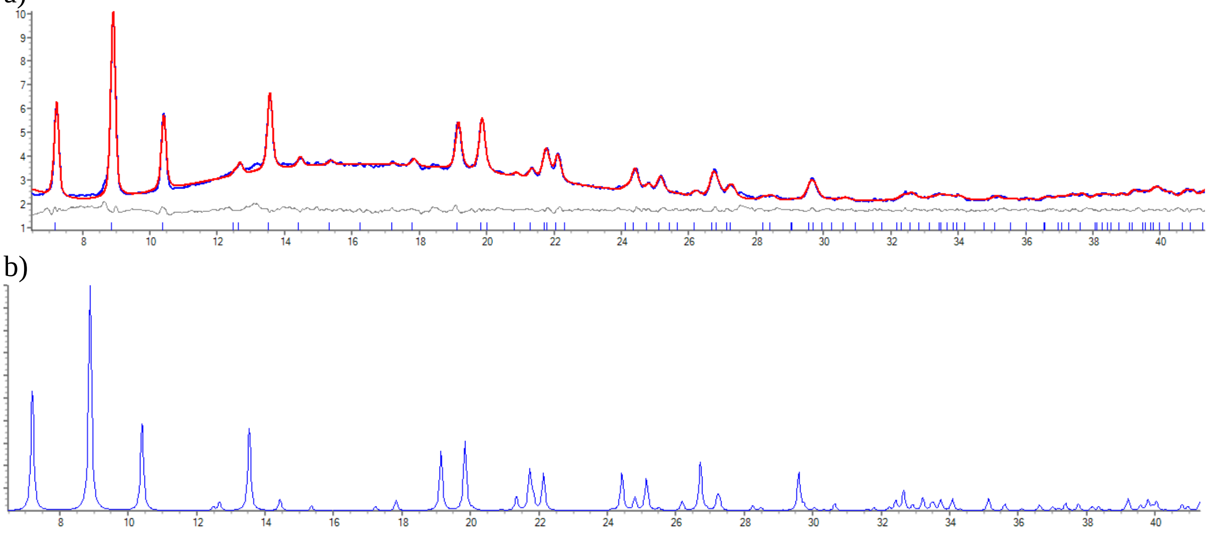

Figure S63. a) Pawley refinement analysis of **1-La.** Blue experimental data, Red Model, and grey difference; b) theoretical powder X-ray diffraction pattern of **1-La.** Pawley refinement Rwp 1.754; Rwp′ = 10.190.

a)
b)

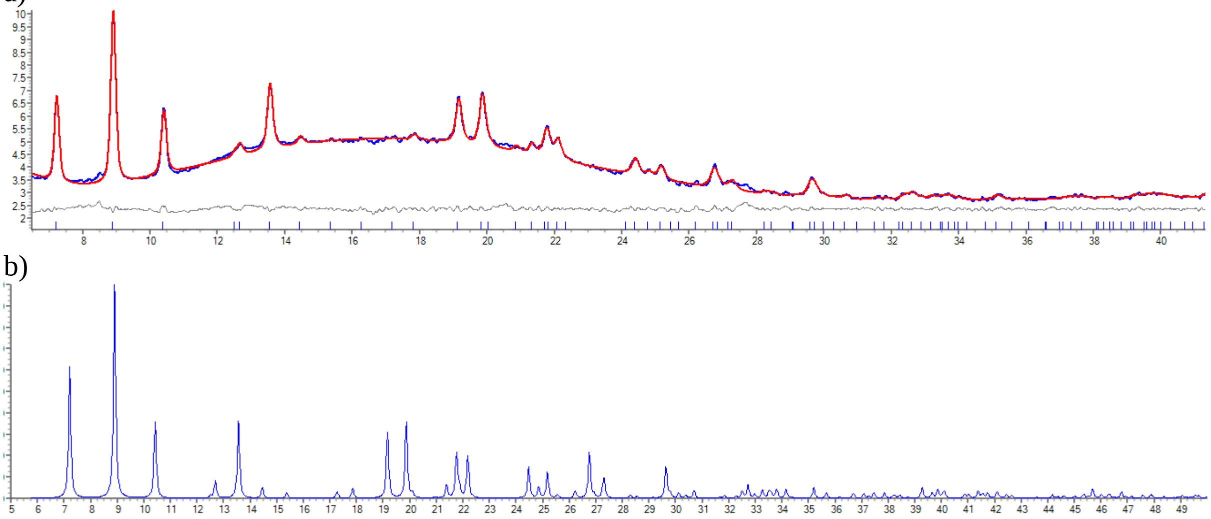

Figure S64. a) Pawley refinement analysis of **1-Ce.** Blue experimental data, Red Model, and grey difference; b) theoretical powder X-ray diffraction pattern of **1-Ce.** Pawley refinement Rwp 1.754; Rwp′ = 11.558.

a)

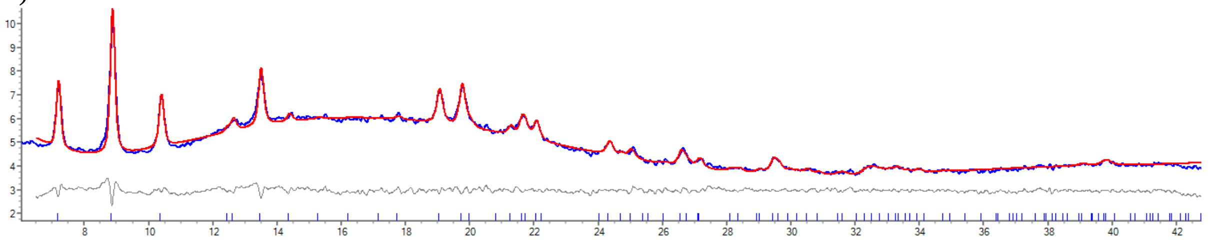

b)

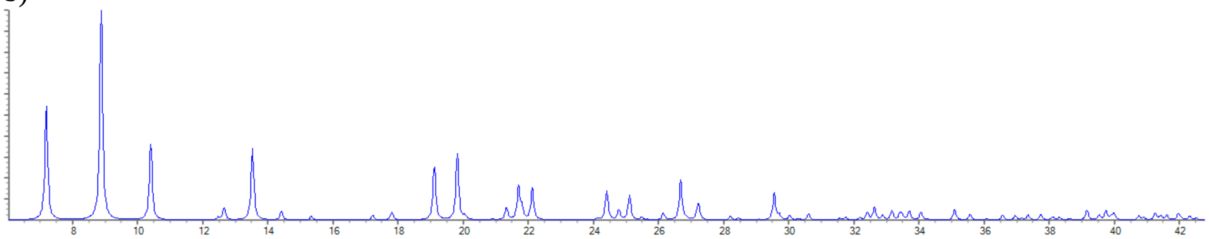

Figure S65. a) Pawley refinement analysis of **1-Pr.** Blue experimental data, Red Model, and grey difference; b) theoretical powder X-ray diffraction pattern of **1-Pr.** Pawley refinement Rwp 2.156; Rwp′ = 18.414.

a)

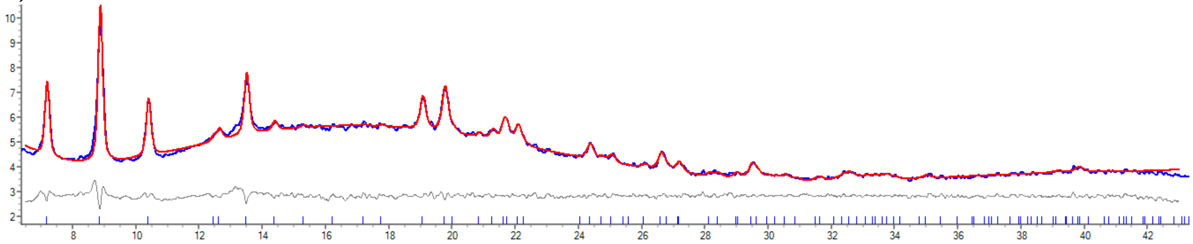

b)

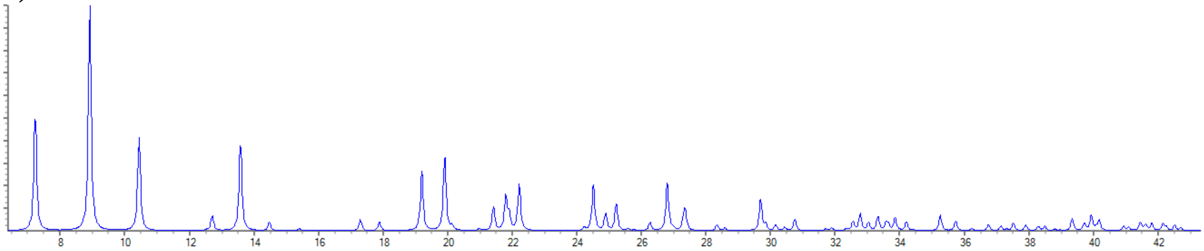

Figure S66. a) Pawley refinement analysis of **1-Nd.** Blue experimental data, Red Model, and grey difference; b) theoretical powder X-ray diffraction pattern of **1-Nd.** Pawley refinement Rwp 2.104; Rwp′ = 16.825.

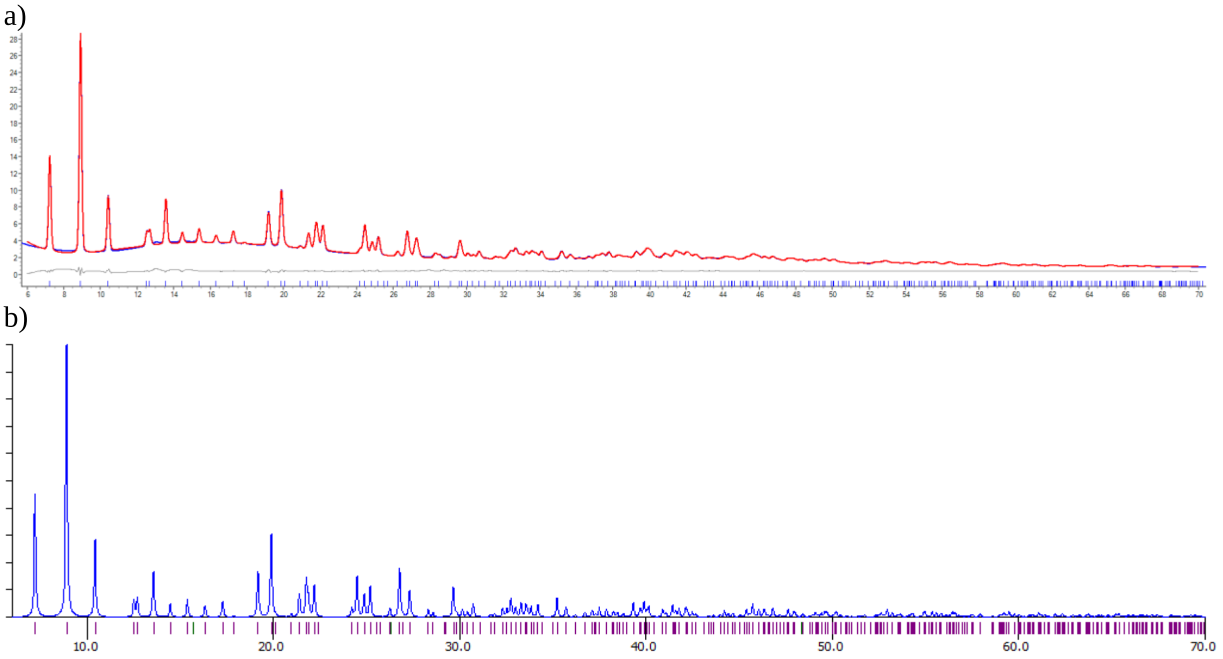

Figure S67. a) Pawley refinement analysis of **1-U.** Blue experimental data, Red Model, and grey difference; b) theoretical powder X-ray diffraction pattern of **1-U.** Pawley refinement Rwp 2.352; Rwp′ = 8.026.

Table S4. Unit cell values obtained from Pawley refinement results.

**Complex**	**a**	**b**	**c**	**α**	**β**	**γ**
**1-La**	14.176(1)	14.176(1)	17.049(1)	90	90	120
**1-Ce**	14.161(3)	14.161(3)	17.035(4)	90	90	120
**1-Pr**	14.238(5)	14.238(5)	17.052(8)	90	90	120
**1-Nd**	14.219(5)	14.219(5)	17.032(8)	90	90	120
**1-U**	14.1432(5)	14.1432(5)	16.9941(6)	90	90	120

7. Magnetism

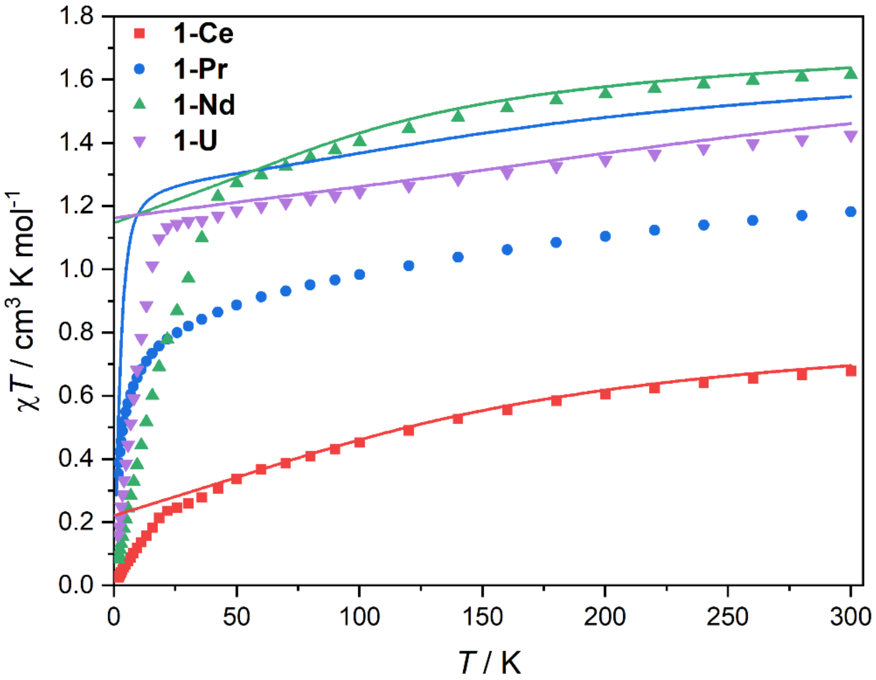

Figure S68. Magnetic susceptibility data of **1-Ce**, **1-Pr**, **1-Nd** and **1-U** in 1 kOe dc field along with CASSCF-SO calculated curves (solid lines).

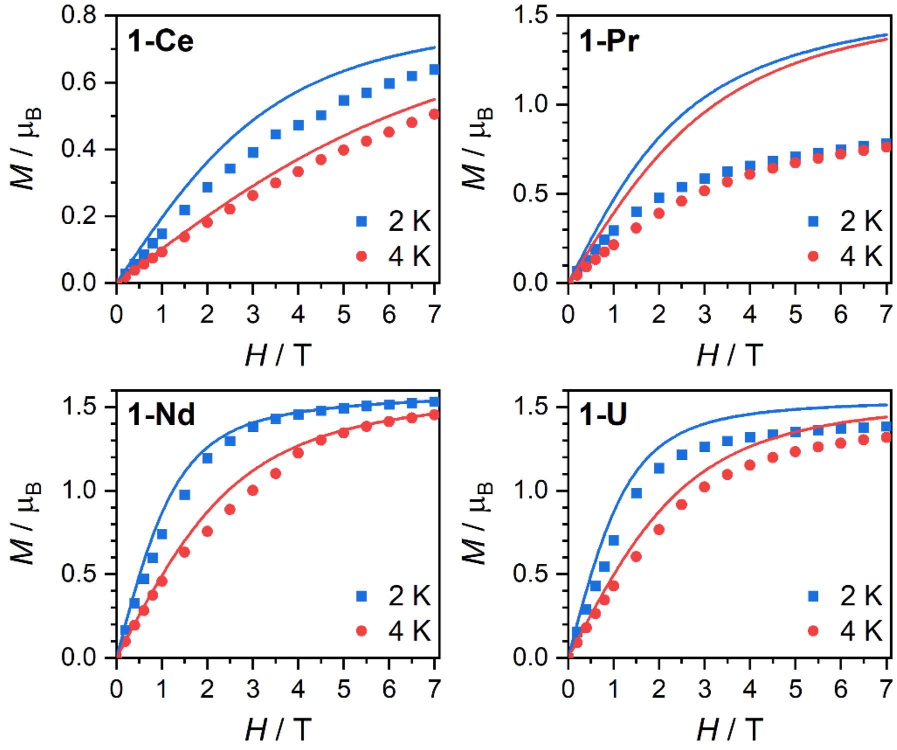

Figure S69. Magnetization *vs*. field for **1-Ce**, **1-Pr**, **1-Nd** and **1-U** at 2 and 4 K along with CASSCF-SO calculated curves (solid lines).

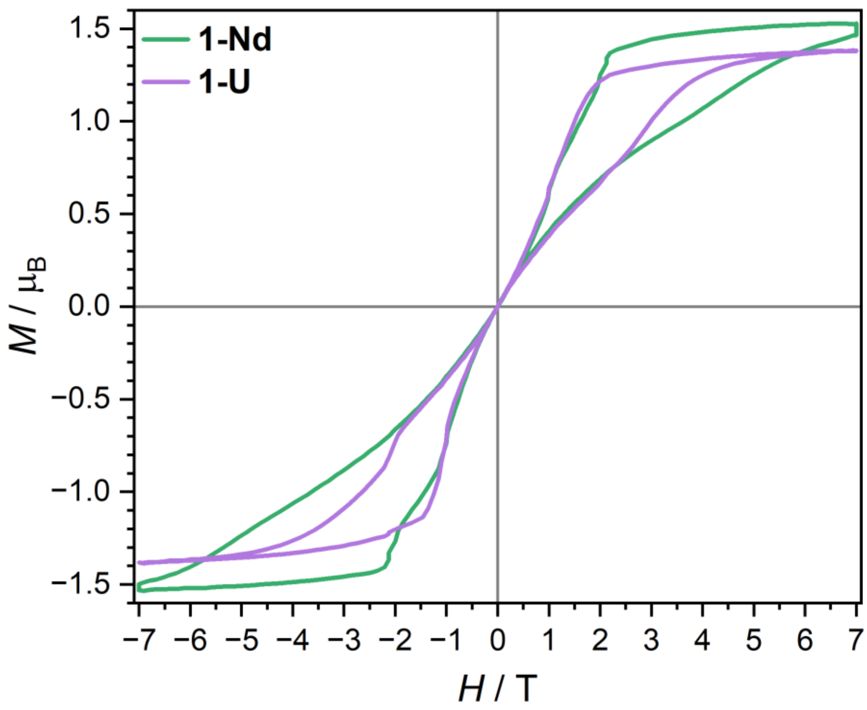

Figure S70. Magnetic hysteresis at 2 K for **1-Nd** and **1-U**.

Table S5. Experimental details for SQUID analysis of paramagnetic **1-M**.

Complex	Molecular Weight (g mol^{-1})	Sample Mass (mg)	Eicosane mass (mg)	Shape correction factor, DC [a]	Shape correction factor, VSM [a]
**1-Ce**	1027.30	31.1	17.1	0.998	0.993
**1-Pr**	1028.11	28.0	14.0	1.029	1.056
**1-Nd**	1031.42	30.5	16.0	0.998	-
**1-U**	1125.21	35.7	18.4	0.999	-

a = Calculated using height and diameter of cylindrical sample using MPMS3 Geometry Correction Simulator.

8. EPR Spectroscopy

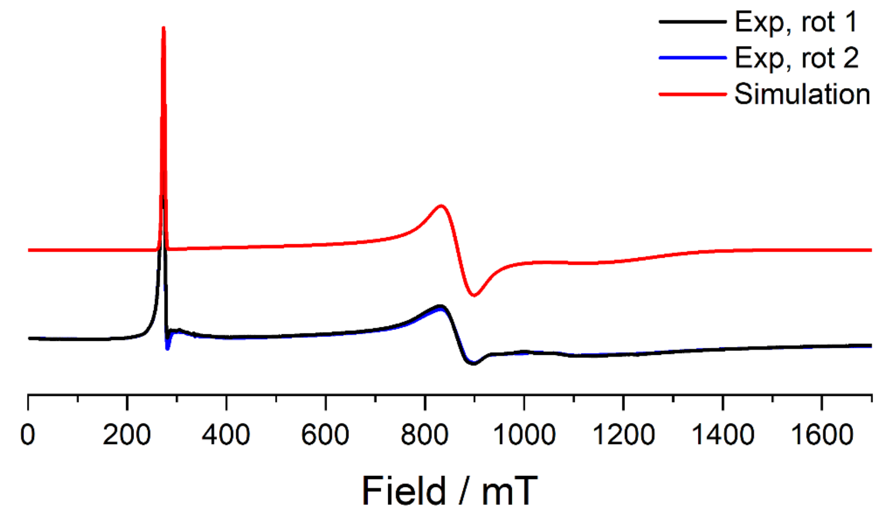

Figure S71. X-band powder EPR spectrum of **1-Ce** at 7 K with two rotations (black, blue) and simulation (red) with *g* = 2.445, 0.786, 0.57. *g*Strain = 0.05, 0.05, 0.12.

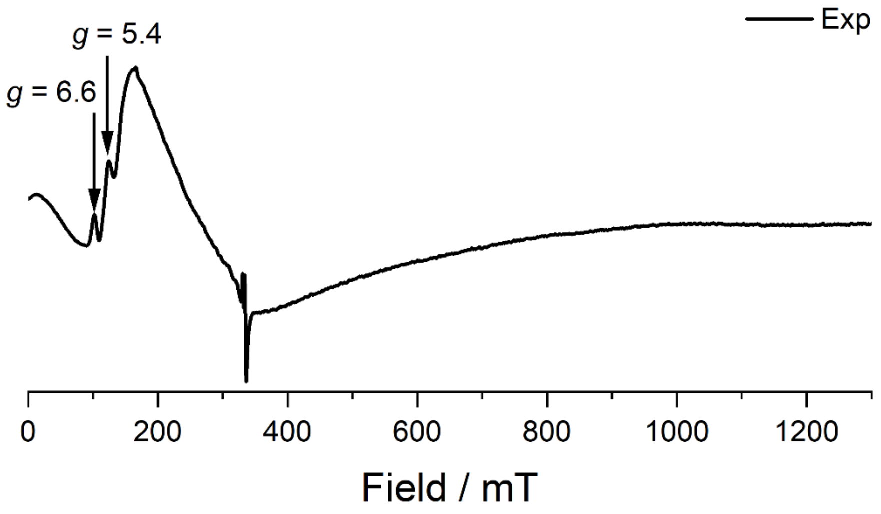

Figure S72. X-band powder EPR spectra of **1-U** at 4 K. CASSCF *g*-values 6.097, 0.055, 0.045.

9. Density Functional Theory (DFT) Calculations

Table S6. Coordinates of the H-atom geometry optimized structure of [La{Si(SiMe$_3$)$_3$}$_3$(THF)$_2$] (La(THF)$_2$).

1.C	0.542040	0.862160	-4.718760
2.C	1.443860	-0.375020	-4.441310
3.C	-2.991870	-1.436550	-4.344300
4.C	-0.344790	0.679990	-3.461840
5.C	-1.805080	-4.101710	-3.160860
6.C	1.251420	4.297290	-3.126220
7.C	-4.872780	-3.465740	-2.836510
8.C	4.020430	-2.381740	-2.827630
9.C	1.798880	-0.170860	-2.947080
10.C	-1.877080	4.334140	-2.775140
11.C	-3.476620	1.399320	-2.327850
12.C	1.155300	-3.964900	-1.551760
13.C	-6.026290	-0.197900	-1.351520
14.C	-0.103410	6.479240	-1.300710
15.C	5.297700	0.104480	-1.378700
16.C	3.337700	2.774370	-0.744770
17.C	5.971250	-2.809340	-0.390210
18.C	-5.544480	-3.609380	0.179570
19.C	3.225010	-5.245680	0.468410
20.C	2.788540	5.432290	0.879740
21.C	-4.162700	1.252600	0.752820
22.C	-0.775120	6.335600	1.715370
23.C	0.469220	-4.111620	1.528910
24.C	-2.890600	-4.333840	1.713360
25.C	-2.962600	4.102010	2.099080
26.C	2.651610	2.627660	2.335900
27.C	5.299460	-2.953030	2.625670
28.C	-4.520350	-1.763410	2.518890
29.C	-1.519040	-0.964450	2.955340
30.C	4.212180	-0.127650	3.495520
31.C	0.242560	0.515430	3.496010
32.C	-0.277060	3.970430	3.736970
33.C	2.491950	-2.708600	4.035560
34.C	-1.722800	-0.313400	4.346410
35.C	-0.252590	-0.121320	4.818510
36.H	0.030160	0.779660	-5.562050
37.H	-3.128430	-1.945070	-5.170980
38.H	2.253950	-0.367760	-5.010480
39.H	1.047860	1.713070	-4.708600
40.H	0.951360	-1.221800	-4.584000
41.H	-2.111710	-1.006180	-4.367140
42.H	-3.688310	-0.751800	-4.263330
43.H	-1.994170	-4.496280	-4.037710
44.H	1.102120	4.874690	-3.904020
45.H	-4.993100	-3.956670	-3.676160
46.H	-1.891960	4.911350	-3.566910
47.H	-1.097110	0.067940	-3.660970
48.H	4.796430	-2.345740	-3.425140
49.H	1.249830	3.359450	-3.410590
50.H	3.293890	-1.841750	-3.203070
51.H	-3.539700	0.951530	-3.197290

52.H	-0.893940	-3.741020	-3.154500
53.H	-0.717270	1.552220	-3.177850
54.H	-1.959940	3.397750	-3.051930
55.H	-5.552990	-2.763400	-2.768500
56.H	3.722460	-3.310700	-2.734760
57.H	2.116350	4.513890	-2.719810
58.H	2.426070	0.584460	-2.819330
59.H	-1.888810	-4.789710	-2.467920
60.H	2.183380	-0.990500	-2.546340
61.H	-4.009080	2.221940	-2.344720
62.H	-0.241080	7.037690	-2.094250
63.H	-6.066820	-0.669170	-2.186920
64.H	-4.964320	-4.083060	-2.080800
65.H	-2.624570	4.568050	-2.186120
66.H	1.864810	-3.901130	-2.224770
67.H	6.019710	0.032690	-2.037270
68.H	-2.540280	1.620160	-2.141380
69.H	4.605190	0.709220	-1.717870
70.H	0.707730	-4.833240	-1.630250
71.H	-6.520090	0.622250	-1.422430
72.H	0.503390	-3.247320	-1.695340
73.H	3.221060	3.271680	-1.581120
74.H	6.730210	-2.769600	-1.008760
75.H	0.773280	6.676950	-0.910010
76.H	-0.804460	6.668320	-0.642340
77.H	-6.407240	-0.741970	-0.658340
78.H	3.073730	1.840390	-0.880610
79.H	-5.294940	-4.153760	-0.596280
80.H	5.656370	0.457460	-0.537660
81.H	5.656350	-3.734650	-0.318360
82.H	4.278020	2.811600	-0.471040
83.H	3.972050	-5.117540	-0.152940
84.H	-6.145910	-2.890190	-0.106160
85.H	2.632580	5.913850	0.064010
86.H	2.784880	-6.100340	0.278300
87.H	6.251960	-2.490490	0.492990
88.H	-5.999110	-4.171800	0.841010
89.H	-4.668690	2.080880	0.617040
90.H	-1.135070	6.597620	0.842180
91.H	3.725120	5.452520	1.089290
92.H	-2.659140	-4.954440	0.991000
93.H	-3.240160	1.470490	1.001230
94.H	-3.394900	4.403320	1.272800
95.H	3.562420	-5.249500	1.388590
96.H	0.048130	-4.974300	1.331510
97.H	2.292160	5.841050	1.592590
98.H	-4.578970	0.729290	1.469260
99.H	-0.196490	-3.396980	1.447260
100.H	0.180360	6.550160	1.752330
101.H	5.921430	-2.561280	1.977700
102.H	-2.069970	-3.992500	2.126120
103.H	-5.130630	-1.060240	2.213000
104.H	5.063430	-3.861440	2.343980
105.H	-1.247080	6.822560	2.422920
106.H	-3.135970	3.146260	2.228700
107.H	-3.425210	-4.802300	2.387940
108.H	2.373850	1.690720	2.262000

109.H	0.825540	-4.123380	2.441770
110.H	3.618410	2.670550	2.490720
111.H	-2.349670	-0.946580	2.417060
112.H	-3.323000	4.605330	2.858740
113.H	4.885920	0.292660	2.921230
114.H	-3.741540	-1.354720	2.951150
115.H	-4.983830	-2.341840	3.160170
116.H	2.181780	3.049440	3.085420
117.H	-1.194650	-1.896760	3.031090
118.H	5.724360	-2.984660	3.508270
119.H	1.211060	0.347730	3.378310
120.H	3.429150	0.457730	3.562750
121.H	0.093510	1.494030	3.511860
122.H	2.280220	-3.619220	3.741750
123.H	0.674030	4.205450	3.756510
124.H	-0.380000	3.010920	3.907700
125.H	1.664050	-2.190280	4.115220
126.H	4.588800	-0.273270	4.388410
127.H	-2.196290	0.552990	4.274900
128.H	-0.753280	4.477920	4.427130
129.H	2.941150	-2.742450	4.906040
130.H	-2.223860	-0.910950	4.956280
131.H	0.198280	-0.979040	5.021090
132.H	-0.177010	0.494330	5.590020
133.La	-0.004170	-0.003770	-0.001100
134.O	0.486440	0.124610	-2.409000
135.O	-0.505140	-0.086590	2.406650
136.Si	-3.086650	-2.652200	-2.804120
137.Si	-0.180000	4.585550	-1.812100
138.Si	4.518160	-1.674270	-1.063910
139.Si	-4.163590	0.199160	-0.917770
140.Si	-2.693410	-1.595490	-0.675820
141.Si	-0.030860	3.189720	0.145040
142.Si	1.940940	-3.789770	0.251780
143.Si	2.711530	-1.605520	0.527420
144.Si	2.209910	3.579320	0.662630
145.Si	-3.929480	-2.832440	0.980380
146.Si	-1.022830	4.405310	1.972410
147.Si	3.675320	-1.854510	2.720600

Table S7. Coordinates of the H-atom geometry optimized structure of [La{Si(SiMe$_3$)$_3$}$_3$(THF)] (La(THF)$_1$).

1.C	0.484282	0.810606	-4.893919
2.C	1.433248	-0.363743	-4.593976
3.C	-3.046926	-1.683659	-4.313201
4.C	-0.328067	0.955735	-3.607599
5.C	-2.011949	-4.248169	-3.120213
6.C	1.129842	4.384698	-3.025749
7.C	-4.925560	-3.572208	-2.928065
8.C	4.387713	-2.619308	-2.630586
9.C	1.777929	-0.129840	-3.133271
10.C	-1.902827	4.598309	-2.687602
11.C	-3.686151	1.483944	-2.202019
12.C	1.153433	-4.001388	-1.491674
13.C	-6.094749	-0.128605	-1.282588
14.C	-0.095044	6.582490	-1.394446
15.C	5.495975	-0.075600	-1.371018
16.C	3.368278	2.746760	-0.621583
17.C	6.212191	-2.785764	-0.305650
18.C	-5.618573	-3.612565	0.488771
19.C	3.130389	-5.305389	0.455341
20.C	2.877306	5.353104	0.907288
21.C	-4.250438	1.283524	0.718203
22.C	-0.838776	6.325234	2.020210
23.C	0.465613	-4.159609	1.420687
24.C	-3.008097	-4.436175	1.682040
25.C	-3.003238	4.260701	2.131538
26.C	2.713626	2.634513	2.294667
27.C	5.204425	-2.997398	2.969412
28.C	-4.319139	-1.857468	2.577306
29.C	4.365126	-0.186471	3.541002
30.C	-0.454830	3.848856	3.722015
31.C	2.430571	-2.469664	4.032244
32.H	-0.166300	0.622362	-5.756205
33.H	-3.313400	-2.286638	-5.196283
34.H	2.328451	-0.359658	-5.227179
35.H	1.059439	1.725927	-5.089574
36.H	0.923026	-1.328648	-4.718089
37.H	-2.024262	-1.313307	-4.473804
38.H	-3.727303	-0.821527	-4.287080
39.H	-2.365487	-4.768667	-4.025232
40.H	0.871388	4.886846	-3.971848
41.H	-5.048403	-3.898377	-3.974160
42.H	-1.903230	5.364862	-3.479733
43.H	-1.260908	0.383221	-3.621629
44.H	5.318718	-2.574834	-3.218849
45.H	1.300122	3.320999	-3.241255
46.H	3.598350	-2.166790	-3.244365
47.H	-3.615849	1.047477	-3.207264
48.H	-0.980232	-3.921599	-3.304924
49.H	-0.529466	1.992906	-3.322972
50.H	-2.087143	3.627500	-3.166105
51.H	-5.745790	-2.883052	-2.688745
52.H	4.136424	-3.680826	-2.499141
53.H	2.085973	4.804424	-2.682691
54.H	2.540550	0.650213	-3.000570
55.H	-1.986492	-4.981396	-2.301960

56.H	2.068712	-1.022769	-2.568916
57.H	-4.379875	2.337374	-2.257160
58.H	-0.010672	7.097641	-2.366163
59.H	-6.204228	-0.642577	-2.247996
60.H	-5.036514	-4.460113	-2.291798
61.H	-2.759392	4.801259	-2.028685
62.H	1.875293	-3.834053	-2.301728
63.H	6.385810	-0.232904	-2.002264
64.H	-2.701735	1.892528	-1.931090
65.H	4.813303	0.592079	-1.913995
66.H	0.715061	-5.001342	-1.634283
67.H	-6.655424	0.818612	-1.343223
68.H	0.331119	-3.277890	-1.606875
69.H	3.214436	3.154987	-1.628711
70.H	6.923490	-2.893679	-1.141267
71.H	0.784309	6.847527	-0.792777
72.H	-0.983442	6.981376	-0.886690
73.H	-6.574343	-0.750417	-0.514235
74.H	3.184440	1.661253	-0.654916
75.H	-5.499563	-4.463066	-0.196108
76.H	5.819968	0.456738	-0.464943
77.H	5.934063	-3.793134	0.032136
78.H	4.430588	2.878687	-0.362965
79.H	3.978705	-5.252234	-0.241342
80.H	-6.276882	-2.880941	0.003019
81.H	2.749083	5.949002	-0.007198
82.H	2.600357	-6.253384	0.266853
83.H	6.741959	-2.287006	0.516125
84.H	-6.130309	-3.985077	1.391465
85.H	-4.888524	2.173977	0.606832
86.H	-1.434026	6.813633	1.236566
87.H	3.951611	5.345699	1.153946
88.H	-2.657643	-5.099000	0.878544
89.H	-3.229764	1.643387	0.917931
90.H	-3.506075	4.503848	1.185157
91.H	3.537711	-5.350810	1.475393
92.H	0.029416	-5.154125	1.239804
93.H	2.352939	5.872436	1.721983
94.H	-4.591245	0.732554	1.602957
95.H	-0.333561	-3.423629	1.243709
96.H	0.208362	6.624320	1.881570
97.H	6.118921	-2.571572	2.534958
98.H	-2.143898	-4.178895	2.307662
99.H	-5.119962	-1.130455	2.383852
100.H	5.045676	-3.984895	2.516579
101.H	-1.181504	6.723624	2.989597
102.H	-3.320516	3.253689	2.431042
103.H	-3.713311	-5.009592	2.305689
104.H	2.519845	1.557857	2.173919
105.H	0.745253	-4.102882	2.480280
106.H	3.787099	2.743338	2.513718
107.H	-3.369076	4.965839	2.895755
108.H	5.061765	0.346235	2.878991
109.H	-3.449428	-1.308985	2.966585
110.H	-4.675334	-2.530533	3.374118
111.H	2.153406	2.980690	3.172012
112.H	5.388703	-3.142405	4.047235

113.H	3.558515	0.507623	3.808396
114.H	2.234019	-3.539854	3.880043
115.H	0.574970	4.188897	3.898237
116.H	-0.477084	2.756328	3.843290
117.H	1.468393	-1.939365	3.995580
118.H	4.908365	-0.436663	4.466768
119.H	-1.083631	4.286688	4.514134
120.H	2.837742	-2.354703	5.049780
121.La	0.039440	0.024563	-0.203540
122.O	0.518284	0.361273	-2.540569
123.Si	-3.191412	-2.796428	-2.763040
124.Si	-0.250440	4.713707	-1.740016
125.Si	4.732147	-1.768788	-0.946623
126.Si	-4.273559	0.252635	-0.874539
127.Si	-2.757680	-1.601831	-0.675831
128.Si	-0.055066	3.239348	0.187785
129.Si	1.920457	-3.849593	0.243106
130.Si	2.789627	-1.639221	0.537268
131.Si	2.266447	3.560570	0.700886
132.Si	-3.931256	-2.904566	1.029484
133.Si	-1.111329	4.437883	2.030046
134.Si	3.721224	-1.812179	2.788999

Table S8. Coordinates of the H-atom geometry optimized structure of [La{Si(SiMe$_3$)$_3$}$_3$(THF)$_0$] (La(THF)$_0$).

1.C	-2.877494	-1.490622	-4.276766
2.C	-1.900905	-4.153939	-3.222092
3.C	1.136661	4.205650	-3.124427
4.C	-4.800879	-3.459909	-3.022200
5.C	4.006115	-2.233112	-2.782767
6.C	-1.866634	4.451191	-2.810054
7.C	-3.525591	1.460428	-2.236548
8.C	1.100325	-4.015172	-1.486976
9.C	-5.994932	-0.099937	-1.335374
10.C	-0.073989	6.456310	-1.506759
11.C	5.417551	0.053611	-1.387670
12.C	3.388722	2.762419	-0.647537
13.C	6.011423	-2.762898	-0.581010
14.C	-5.558535	-3.627276	0.383090
15.C	3.120851	-5.260307	0.441861
16.C	2.852991	5.349472	0.894036
17.C	-4.178944	1.318219	0.677904
18.C	-0.841216	6.296459	1.903874
19.C	0.444510	-4.156096	1.425167
20.C	-3.001954	-4.404433	1.722369
21.C	-2.976337	4.219013	2.137824
22.C	2.736636	2.620389	2.266934
23.C	5.238736	-2.931379	2.822813
24.C	-4.383526	-1.827307	2.513112
25.C	4.305099	-0.186360	3.556833
26.C	-0.386763	3.882425	3.667707
27.C	2.479310	-2.562677	4.001529
28.H	-2.885777	-2.072776	-5.212384
29.H	-1.943708	-0.911068	-4.253805
30.H	-3.715741	-0.782158	-4.327056
31.H	-2.279788	-4.639722	-4.136045
32.H	0.899343	4.742650	-4.057252
33.H	-4.916936	-3.748248	-4.079867
34.H	-1.869816	5.241594	-3.578529
35.H	4.826264	-2.063666	-3.499012
36.H	1.227480	3.137631	-3.369385
37.H	3.116266	-1.714216	-3.167223
38.H	-3.475548	1.012507	-3.236759
39.H	-0.870046	-3.833971	-3.420046
40.H	-1.984328	3.488394	-3.323457
41.H	-5.619912	-2.776596	-2.763641
42.H	3.794306	-3.311023	-2.778517
43.H	2.119219	4.561039	-2.784980
44.H	-1.871267	-4.912645	-2.427836
45.H	-4.165978	2.353968	-2.298588
46.H	0.012724	6.968748	-2.479419
47.H	-6.097031	-0.619002	-2.298344
48.H	-4.918452	-4.370551	-2.419169
49.H	-2.747928	4.602401	-2.171398
50.H	1.808250	-3.867351	-2.311729
51.H	6.312643	-0.140398	-2.001027
52.H	-2.516394	1.811531	-1.971168
53.H	4.782862	0.757627	-1.940719
54.H	0.658225	-5.017610	-1.597225
55.H	-6.547407	0.851117	-1.409614

56.H	0.279506	-3.292023	-1.610099
57.H	3.259449	3.199122	-1.645440
58.H	6.681122	-2.841330	-1.453717
59.H	0.799337	6.728780	-0.900335
60.H	-0.968197	6.851397	-1.005967
61.H	-6.488921	-0.711783	-0.568219
62.H	3.185565	1.682861	-0.720101
63.H	-5.393875	-4.471516	-0.299597
64.H	5.746102	0.550168	-0.464379
65.H	5.702167	-3.777406	-0.297976
66.H	4.448346	2.867745	-0.367367
67.H	3.953484	-5.202286	-0.272544
68.H	-6.204943	-2.902486	-0.128186
69.H	2.717870	5.944990	-0.019435
70.H	2.597008	-6.214222	0.265859
71.H	6.599344	-2.345772	0.247621
72.H	-6.105317	-4.013525	1.258980
73.H	-4.805337	2.213821	0.543554
74.H	-1.447520	6.756201	1.111854
75.H	3.927873	5.354503	1.138708
76.H	-2.628383	-5.085285	0.945075
77.H	-3.160165	1.673456	0.896389
78.H	-3.511594	4.446654	1.205631
79.H	3.547100	-5.298052	1.453916
80.H	0.019612	-5.155689	1.244358
81.H	2.324432	5.860239	1.710762
82.H	-4.546377	0.779707	1.560062
83.H	-0.365592	-3.431256	1.251044
84.H	0.201088	6.604994	1.752198
85.H	6.115708	-2.453980	2.364994
86.H	-2.155440	-4.129630	2.364328
87.H	-5.178074	-1.108796	2.269050
88.H	5.096671	-3.907507	2.341224
89.H	-1.183788	6.713926	2.865432
90.H	-3.280461	3.216491	2.464929
91.H	-3.721928	-4.964600	2.341200
92.H	2.544555	1.542960	2.147222
93.H	0.728877	-4.098021	2.483057
94.H	3.812325	2.729505	2.475537
95.H	-3.316032	4.936074	2.903288
96.H	4.984831	0.385003	2.909772
97.H	-3.538165	-1.267116	2.937683
98.H	-4.773963	-2.490865	3.301515
99.H	2.186185	2.965962	3.150643
100.H	5.475862	-3.106725	3.885529
101.H	3.482310	0.477577	3.851074
102.H	2.311563	-3.630749	3.806448
103.H	0.640350	4.244350	3.811552
104.H	-0.389289	2.794889	3.829439
105.H	1.500055	-2.063140	4.020337
106.H	4.859365	-0.455807	4.470756
107.H	-1.007105	4.337994	4.456570
108.H	2.915682	-2.479282	5.010133
109.La	-0.002313	-0.005280	-0.006536
110.Si	-3.064012	-2.705012	-2.815937
111.Si	-0.230868	4.588699	-1.849707
112.Si	4.568725	-1.617463	-1.065931

113.Si	-4.178592	0.275605	-0.906315
114.Si	-2.687952	-1.595201	-0.667545
115.Si	-0.033514	3.177900	0.140499
116.Si	1.882337	-3.830045	0.231345
117.Si	2.709830	-1.593158	0.526630
118.Si	2.279767	3.547228	0.676133
119.Si	-3.912478	-2.896774	1.004867
120.Si	-1.091164	4.409080	1.973725
121.Si	3.706023	-1.802157	2.752445

Table S9. Computed δ_{iso}, σ_{iso}, σ_d, σ_p, and σ_{so} values for La(THF)$_2$ using the functionals BP86, SAOP, and B3LYP-HFXX (XX = 10, 15, 20, 25, 30, 35, 40, 45 or 50%).

Functional	Entry	δ_{iso}	σ_{iso}	σ_d	σ_p	σ_{so}	Entry	δ_{iso}	σ_{iso}	σ_d	σ_p	σ_{so}	Entry	δ_{iso}	σ_{iso}	σ_d	σ_p	σ_{so}
BP86	La-Si	-90.15	435.06	876.03	-423.80	-17.17	La-Si	-89.25	434.16	876.08	-425.21	-16.71	La-Si	-90.70	435.61	876.31	-423.74	-16.96
SAOP		-93.40	495.07	866.79	-358.23	-13.50		-93.38	495.05	866.97	-358.87	-13.06		-94.23	495.9	866.18	-356.85	-13.43
B3LYPHF10		-94.49	432.16	881.22	-430.61	-18.46		-94.39	432.06	881.07	-431.10	-17.91		-95.10	432.77	881.20	-430.21	-18.22
B3LYPHF15		-96.51	439.11	881.37	-423.31	-18.95		-96.44	439.04	881.19	-423.74	-18.41		-96.99	439.59	881.35	-423.03	-18.73
B3LYPHF20		-98.18	445.70	881.51	-416.33	-19.48		-98.14	445.66	881.31	-416.71	-18.94		-98.55	446.07	881.50	-416.16	-19.28
B3LYPHF25		-99.64	452.07	881.66	-409.55	-20.04		-99.55	451.98	881.45	-409.94	-19.52		-99.85	452.28	883.54	-409.53	-19.85
B3LYPHF30		-100.91	458.23	881.79	-402.94	-20.61		-100.68	458.00	881.62	-403.50	-20.12		-101.03	458.35	881.81	-403.04	-20.42
B3LYPHF35		-101.94	464.14	881.94	-396.60	-21.20		-101.67	463.87	881.72	-397.13	-20.73		-101.96	464.16	881.93	-396.76	-21.02
B3LYPHF40		-102.80	469.86	882.07	-390.43	-21.78		-102.54	469.60	881.83	-390.89	-21.34		-102.73	469.79	882.07	-390.67	-21.61
B3LYPHF45		-101.90	475.55	882.15	-384.28	-22.32		-101.63	475.28	881.98	-384.75	-21.95		-101.66	475.31	882.28	-384.71	-22.26
B3LYPHF50		-102.50	481.04	882.19	-378.20	-22.95		-102.27	480.81	882.06	-378.65	-22.61		-102.25	480.79	882.32	-378.65	-22.88
BP86	$SiMe_3$	10.95	333.96	869.02	-541.78	6.72	$SiMe_3$	10.05	334.86	869.03	-540.86	6.69	$SiMe_3$	9.98	334.93	869.45	-541.12	6.59
SAOP		6.77	394.90	866.34	-478.81	7.37		5.50	396.17	865.91	-477.13	7.38		6.41	395.26	866.56	-478.58	7.28
B3LYPHF10		11.23	326.44	875.61	-556.55	7.38		10.68	326.99	875.60	-556.02	7.41		10.57	327.10	875.67	-555.85	7.28
B3LYPHF15		10.43	332.17	875.58	-550.92	7.51		10.01	332.59	875.58	-550.52	7.53		9.95	332.65	875.67	-550.42	7.40
B3LYPHF20		9.69	337.83	875.54	-545.34	7.63		9.41	338.11	875.55	-545.07	7.63		9.40	338.12	875.66	-545.05	7.51
B3LYPHF25		9.05	343.38	875.51	-539.85	7.73		8.82	343.61	875.50	-539.60	7.72		8.91	343.52	875.65	-539.75	7.61
B3LYPHF30		8.40	348.92	875.45	-534.37	7.83		8.08	349.24	875.30	-533.89	7.83		8.45	348.87	875.63	-534.47	7.71
B3LYPHF35		7.83	354.37	875.37	-528.92	7.92		7.54	354.66	875.22	-528.47	7.92		7.99	354.21	875.52	-529.11	7.80
B3LYPHF40		7.26	359.8	875.32	-523.54	8.02		7.03	360.03	875.15	-523.13	8.00		7.57	359.49	875.49	-523.89	7.89
B3LYPHF45		8.42	365.23	875.17	-518.05	8.12		8.36	365.29	875.09	-517.86	8.06		8.84	364.81	875.43	-518.59	7.97
B3LYPHF50		7.89	370.65	875.08	-512.63	8.20		7.85	370.69	875.02	-512.52	8.19		8.40	370.14	875.31	-513.22	8.05
BP86	$SiMe_3$	-3.58	348.49	868.15	-528.94	6.69	$SiMe_3$	-3.05	347.96	868.08	-529.36	9.233	$SiMe_3$	-3.91	348.82	868.02	-528.43	9.22
SAOP		-4.83	406.50	865.09	-468.56	7.38		-4.26	405.93	864.70	-468.69	9.920		-4.82	406.49	864.64	-468.07	9.92
B3LYPHF10		-2.79	340.46	876.33	-546.20	7.41		-2.08	339.75	876.14	-546.67	10.28		-3.13	340.80	876.23	-545.74	10.31
B3LYPHF15		-2.93	345.53	876.43	-541.42	7.53		-2.26	344.86	876.24	-541.88	10.50		-3.24	345.84	876.31	-540.98	10.52
B3LYPHF20		-3.00	350.52	876.52	-536.70	7.63		-2.35	349.87	876.34	-537.14	10.68		-3.28	350.80	876.38	-536.28	10.70
B3LYPHF25		-3.01	355.44	876.63	-532.05	7.72		-2.34	354.77	876.38	-532.45	10.85		-3.24	355.67	876.44	-531.63	10.86
B3LYPHF30		-2.93	360.25	876.68	-527.46	7.83		-2.33	359.65	876.45	-527.81	11.01		-3.20	360.52	876.46	-526.97	11.03
B3LYPHF35		-2.89	365.09	876.67	-522.74	7.92		-2.31	364.51	876.51	-523.18	11.18		-3.07	365.27	876.46	-522.39	11.19
B3LYPHF40		-2.78	369.84	876.74	-518.22	8.00		-2.22	369.28	876.60	-518.67	11.35		-2.92	369.98	876.53	-517.90	11.35
B3LYPHF45		-0.93	374.58	876.69	-513.60	8.06		-0.34	373.99	876.69	-514.21	11.51		-0.98	374.63	876.57	-513.45	11.50
B3LYPHF50		-0.78	379.32	876.62	-508.94	8.19		-0.22	378.76	876.48	-509.36	11.65		-0.88	379.42	876.54	-508.82	11.70
BP86	$SiMe_3$	10.45	334.46	869.38	-541.49	6.57	$SiMe_3$	10.04	334.87	869.14	-541.04	6.77	$SiMe_3$	10.27	334.64	868.99	-541.02	6.67
SAOP		6.81	394.86	866.47	-478.87	7.26		6.30	395.37	866.63	-478.72	7.46		5.44	396.23	865.73	-476.82	7.32
B3LYPHF10		11.00	326.67	875.66	-556.24	7.26		10.44	327.23	875.59	-555.78	7.43		10.97	326.70	875.6	-556.28	7.38
B3LYPHF15		10.32	332.28	875.64	-550.74	7.38		9.73	332.87	875.57	-550.24	7.54		10.26	332.34	875.58	-550.75	7.51
B3LYPHF20		-1.15	348.67	875.50	-534.50	7.67		9.69	337.83	875.62	-545.29	7.50		9.08	338.44	875.55	-544.76	7.64
B3LYPHF25		9.11	343.32	875.59	-539.86	7.59		8.58	343.85	875.50	-539.37	7.72		8.87	343.56	875.50	-539.66	7.72
B3LYPHF30		3.27	354.05	875.46	-529.16	7.75		7.91	349.41	875.45	-533.87	7.83		8.29	349.03	875.45	-534.23	7.82
B3LYPHF35		8.15	354.05	875.46	-529.16	7.75		7.37	354.83	875.39	-528.46	7.91		7.71	354.49	875.37	-528.82	7.93
B3LYPHF40		7.69	359.37	875.41	-523.88	7.85		6.89	360.17	875.33	-523.14	7.99		7.17	359.89	875.32	-523.45	8.02
B3LYPHF45		9.00	364.65	875.31	-518.63	7.97		8.22	365.43	875.26	-517.89	8.05		8.47	365.18	875.22	-518.10	8.07
B3LYPHF50		8.52	370.02	875.22	-513.32	8.12		7.70	370.84	875.15	-512.45	8.14		7.93	370.61	875.08	-512.64	8.18

Table S10. Computed δ_{iso}, σ_{iso}, σ_d, σ_p, and σ_{so} values for La(THF)$_1$ using the functionals BP86, SAOP, and B3LYP-HFXX (XX = 10, 15, 20, 25, 30, 35, 40, 45 or 50%).

Functional	Entry	δ_{iso}	σ_{iso}	σ_d	σ_p	σ_{so}	Entry	δ_{iso}	σ_{iso}	σ_d	σ_p	σ_{so}	Entry	δ_{iso}	σ_{iso}	σ_d	σ_p	σ_{so}
BP86	La-Si	-49.94	394.85	874.81	-459.07	-20.89	La-Si	-50.09	395	875.37	-460.43	-19.93	La-Si	-54.6	399.51	875.14	-455.19	-20.44
SAOP		-65.89	467.56	864.7	-380.4	-16.74		-67.15	468.82	865.12	-380.54	-15.75		-69.87	471.54	865.33	-377.6	-16.19
B3LYPHF10		-54.73	392.40	879.2	-463.2	-23.5		-54.83	392.5	879.1	-464.2	-22.5		-59.43	397.1	879.2	-459	-23
B3LYPHF15		-57.80	400.40	879.3	-454.5	-24.4		-57.9	400.5	879.3	-455.4	-23.4		-62.5	405.1	879.3	-450.3	-23.9
B3LYPHF20		-60.48	408.00	879.3	-445.9	-25.4		-60.48	408	879.3	-447	-24.3		-64.98	412.5	879.4	-442	-24.9
B3LYPHF25		-62.67	415.10	879.4	-437.9	-26.4		-62.57	415	879.5	-439.1	-25.3		-67.07	419.5	879.5	-434.1	-25.9
B3LYPHF30		-64.58	421.90	879.5	-430.2	-27.4		-64.48	421.8	879.6	-431.4	-26.4		-68.88	426.2	879.7	-426.5	-27
B3LYPHF35		-66.30	428.50	879.4	-422.5	-28.4		-66.2	428.4	879.6	-423.8	-27.4		-70.8	433	879.7	-418.8	-28
B3LYPHF40		-67.74	434.80	879.5	-415.2	-29.4		-67.64	434.7	879.7	-416.6	-28.4		-72.14	439.2	879.9	-411.6	-29.1
B3LYPHF45		-67.55	441.20	879.6	-407.9	-30.5		-67.35	441	879.8	-409.4	-29.4		-71.95	445.6	879.9	-404.2	-30.1
B3LYPHF50		-68.66	447.20	879.6	-400.9	-31.5		-68.36	446.9	879.9	-402.5	-30.5		-73.06	451.6	880	-397.3	-31.1
BP86	$SiMe_3$	4.66	340.25	869.58	-534.92	5.59	$SiMe_3$	1.94	342.97	869.5	-532.19	5.66	$SiMe_3$	3.66	341.25	869.46	-533.57	5.35
SAOP		0.81	400.86	864.98	-470.57	6.44		-1.74	403.41	864.38	-467.49	6.51		-0.26	401.93	864.74	-468.98	6.17
B3LYPHF10		5.47	332.20	874.8	-548.6	6.02		3.07	334.6	874.7	-546.2	6.08		4.77	332.9	874.7	-547.6	5.73
B3LYPHF15		5.20	337.40	874.8	-543.5	6.1		3	339.6	874.6	-541.2	6.2		4.6	338	874.7	-542.5	5.8
B3LYPHF20		5.02	342.50	874.6	-538.4	6.2		2.92	344.6	874.6	-536.2	6.2		4.42	343.1	874.5	-537.3	5.9
B3LYPHF25		4.93	347.50	874.6	-533.4	6.3		2.93	349.5	874.5	-531.3	6.3		4.33	348.1	874.5	-532.3	6.00
B3LYPHF30		4.82	352.50	874.5	-528.4	6.4		3.02	354.3	874.4	-526.5	6.4		4.32	353	874.4	-527.4	6.00
B3LYPHF35		4.80	357.40	874.1	-523.2	6.5		3.1	359.1	874.3	-521.7	6.5		4.3	357.9	874.3	-522.5	6.1
B3LYPHF40		4.86	362.20	874.1	-518.4	6.5		3.26	363.8	874.2	-516.9	6.5		4.26	362.8	874.2	-517.6	6.2
B3LYPHF45		6.75	366.90	873.9	-513.6	6.6		5.15	368.5	874.1	-512.2	6.6		6.15	367.5	874	-512.8	6.2
B3LYPHF50		6.94	371.60	873.8	-508.8	6.7		5.34	373.2	874	-507.5	6.6		6.34	372.2	873.9	-508	6.3
BP86	$SiMe_3$	-1.44	346.35	869.49	-531.13	7.99	$SiMe_3$	-1.63	346.54	869.55	-531.17	8.16	$SiMe_3$	-1.73	346.64	869.47	-531.04	8.21
SAOP		-4.58	406.25	865.8	-468.63	9.09		-4.78	406.45	866.11	-468.91	9.24		-4.61	406.28	865.75	-468.8	9.33
B3LYPHF10		-1.93	339.60	875.1	-544.6	9.1		-1.93	339.6	875.3	-545.1	9.4		-2.23	339.9	875.1	-544.6	9.4
B3LYPHF15		-2.10	344.70	875.1	-539.8	9.4		-2.1	344.7	875.4	-540.2	9.6		-2.4	345	875.2	-539.9	9.7
B3LYPHF20		-2.38	349.90	875.1	-534.9	9.6		-2.28	349.8	875.3	-535.4	9.9		-2.38	349.9	875.1	-535.2	9.9
B3LYPHF25		-2.37	354.80	875.1	-530.1	9.8		-2.37	354.8	875.4	-530.7	10.1		-2.37	354.8	875.2	-530.6	10.1
B3LYPHF30		-2.28	359.60	875.1	-525.5	10		-2.38	359.7	875.4	-526	10.3		-2.28	359.6	875.2	-526	10.3
B3LYPHF35		-2.20	364.40	874.9	-520.7	10.1		-2.4	364.6	875.4	-521.3	10.5		-2.1	364.3	875.2	-521.4	10.5
B3LYPHF40		-2.04	369.10	874.9	-516.1	10.3		-2.34	369.4	875.4	-516.6	10.7		-1.94	369	875.2	-516.9	10.7
B3LYPHF45		-0.15	373.80	874.9	-511.7	10.5		-0.25	373.9	875.2	-512.2	10.9		-0.05	373.7	875.1	-512.3	10.9
B3LYPHF50		0.14	378.40	874.9	-507.2	10.7		0.04	378.5	875.2	-507.7	11.1		0.14	378.4	875.1	-507.9	11.2
BP86	$SiMe_3$	5.55	339.36	869.62	-536.43	6.17	$SiMe_3$	4.23	340.68	869.63	-535.22	6.27	$SiMe_3$	5.91	339	869.51	-536.82	6.31
SAOP		1.83	399.84	865.23	-472.44	7.05		0.35	401.32	865.47	-471.31	7.16		2.06	399.61	865.09	-472.69	7.2
B3LYPHF10		5.37	332.30	874.4	-549	6.9		4.07	333.6	874.3	-547.7	6.9		5.47	332.2	874.2	-549	7.00
B3LYPHF15		5.00	337.60	874.3	-543.7	7.00		3.9	338.7	874.2	-542.5	.00		5.1	337.5	874.1	-543.8	7.1
B3LYPHF20		4.72	342.80	874.0	-538.3	7.1		3.62	343.9	874	-537.2	7.1		4.82	342.7	873.9	-538.5	7.3
B3LYPHF25		4.63	347.80	873.9	-533.2	7.2		3.63	348.8	873.8	-532.2	7.2		4.73	347.7	873.8	-533.4	7.4
B3LYPHF30		4.52	352.80	873.7	-528.2	7.3		3.52	353.8	873.7	-527.2	7.3		4.62	352.7	873.6	-528.3	7.5
B3LYPHF35		4.70	357.50	873.5	-523.3	7.3		3.6	358.6	873.4	-522.2	7.3		4.5	357.7	873.4	-523.2	7.6
B3LYPHF40		4.76	362.30	873.3	-518.4	7.4		3.66	363.4	873.3	-517.2	7.4		4.46	362.6	873.3	-518.3	7.6
B3LYPHF45		6.55	367.10	873.1	-513.5	7.5		5.45	368.2	873.1	-512.3	7.5		6.15	367.5	873	-513.3	7.7
B3LYPHF50		6.74	371.80	872.9	-508.7	7.6		5.54	373	872.9	-507.4	7.5		6.24	372.3	872.9	-508.4	7.8

Table S11. Computed δ_{iso}, σ_{iso}, σ_d, σ_p, and σ_{so} values for La(THF)$_0$ using the functionals BP86, SAOP, and B3LYP-HFXX (XX = 10, 15, 20, 25, 30, 35, 40, 45 or 50%).

Functional	Entry	δ_{iso}	σ_{iso}	σ_d	σ_p	σ_{so}	Entry	δ_{iso}	σ_{iso}	σ_d	σ_p	σ_{so}	Entry	δ_{iso}	σ_{iso}	σ_d	σ_p	σ_{so}
BP86	La-Si	-15.98	360.89	875.36	-492.22	-22.25	La-Si	-15.8	360.71	875.38	-492.54	-22.13	La-Si	-15.91	360.82	875.33	-492.34	-22.17
SAOP		-47.56	449.23	865.29	-398.93	-17.13		-47.17	448.84	865.27	-399.32	-17.10		-47.35	449.02	865.27	-399.16	-17.09
B3LYPHF10		-24.33	362.00	879.69	-492.68	-25.00		-24.00	361.67	879.70	-493.07	-24.96		-24.21	361.88	879.7	-492.89	-24.94
B3LYPHF15		-29.10	371.70	879.85	-482.15	-26.00		-29.10	371.70	879.85	-482.61	-25.96		-28.93	371.53	879.86	-482.41	-25.93
B3LYPHF20		-32.90	380.42	880.00	-472.44	-27.13		-32.42	379.94	880.00	-472.95	-27.12		-32.68	380.2	880.01	-472.74	-27.07
B3LYPHF25		-36.51	388.94	879.75	-462.44	-28.37		-35.99	388.42	879.74	-462.96	-28.35		-36.23	388.66	879.74	-462.76	-28.32
B3LYPHF30		-39.34	396.66	879.88	-453.57	-29.65		-38.78	396.10	879.87	-454.12	-29.65		-39.02	396.34	879.87	-453.92	-29.61
B3LYPHF35		-41.84	404.04	879.99	-445.00	-30.95		-41.26	403.46	879.99	-445.57	-30.96		-41.48	403.68	879.99	-445.38	-30.93
B3LYPHF40		-44.12	411.18	879.83	-436.36	-32.3		-43.56	410.62	879.81	-436.89	-32.30		-43.76	410.82	879.82	-436.72	-32.28
B3LYPHF45		-44.61	418.26	879.63	-427.75	-33.62		-44.02	417.67	879.63	-428.31	-33.65		-44.24	417.89	879.63	-428.13	-33.61
B3LYPHF50		-46.42	424.96	879.70	-419.77	-34.97		-45.83	424.37	879.70	-420.32	-35.01		-46.04	424.58	879.7	-420.14	-34.98
BP86	$SiMe_3$	7.75	337.16	869.55	-537.54	5.14	$SiMe_3$	7.84	337.07	869.62	-537.7	5.16	$SiMe_3$	7.91	337.00	869.59	-537.73	5.14
SAOP		2.91	398.76	864.98	-472.56	6.34		2.89	398.78	865.05	-472.61	6.34		2.92	398.75	865.03	-472.61	6.33
B3LYPHF10		7.43	330.24	874.6	-550.02	5.67		7.55	330.12	874.6	-550.13	5.66		7.45	330.22	874.59	-550.04	5.67
B3LYPHF15		6.88	335.72	874.48	-544.55	5.79		7.01	335.59	874.47	-544.65	5.77		6.89	335.71	874.47	-544.55	5.79
B3LYPHF20		6.47	341.05	874.35	-539.18	5.88		6.61	340.91	874.35	-539.29	5.85		6.48	341.04	874.34	-539.18	5.88
B3LYPHF25		6.07	346.36	873.93	-533.53	5.96		6.21	346.22	873.89	-533.59	5.92		6.09	346.34	873.9	-533.49	5.93
B3LYPHF30		5.85	351.47	873.79	-528.35	6.02		6.00	351.32	873.74	-528.40	5.98		5.87	351.45	873.75	-528.29	5.99
B3LYPHF35		5.69	356.51	873.65	-523.24	6.10		5.85	356.35	873.57	-523.27	6.05		5.71	356.49	873.59	-523.16	6.06
B3LYPHF40		5.61	361.45	873.38	-518.09	6.16		5.8	361.26	873.35	-518.20	6.11		5.64	361.42	873.35	-518.04	6.11
B3LYPHF45		7.52	366.13	872.91	-513.01	6.23		7.73	365.92	872.91	-513.15	6.16		7.57	366.08	872.9	-512.98	6.16
B3LYPHF50		7.59	370.95	872.71	-508.07	6.31		7.83	370.71	872.7	-508.22	6.22		7.64	370.9	872.69	-508.03	6.24
BP86	$SiMe_3$	-0.06	344.97	869.53	-531.91	7.35	$SiMe_3$	-0.33	345.24	869.48	-531.6	7.36	$SiMe_3$	-0.27	345.18	869.52	-531.7	7.37
SAOP		-4.82	406.49	865.63	-468.28	9.14		-5.05	406.72	865.59	-468.04	9.16		-5.01	406.68	865.61	-468.08	9.15
B3LYPHF10		-0.96	338.63	875.3	-545.64	8.96		-1.13	338.8	875.31	-545.52	9		-1.13	338.8	875.31	-545.5	9
B3LYPHF15		-1.19	343.79	875.33	-540.84	9.3		-1.35	343.95	875.35	-540.75	9.35		-1.36	343.96	875.34	-540.72	9.35
B3LYPHF20		-1.29	348.81	875.36	-536.11	9.57		-1.44	348.96	875.37	-536.04	9.63		-1.46	348.98	875.36	-536.01	9.63
B3LYPHF25		-1.42	353.85	875.28	-531.25	9.82		-1.54	353.97	875.29	-531.18	9.87		-1.55	353.98	875.28	-531.14	9.84
B3LYPHF30		-1.42	358.74	875.3	-526.59	10.03		-1.53	358.85	875.31	-526.55	10.09		-1.54	358.86	875.3	-526.5	10.06
B3LYPHF35		-1.35	363.55	875.31	-521.99	10.23		-1.46	363.66	875.32	-521.96	10.3		-1.48	363.68	875.31	-521.91	10.27
B3LYPHF40		-1.13	368.19	875.17	-517.43	10.45		-1.19	368.25	875.18	-517.43	10.49		-1.24	368.3	875.17	-517.36	10.49
B3LYPHF45		0.55	373.1	875.06	-512.6	10.64		0.5	373.15	875.07	-512.61	10.69		0.46	373.19	875.06	-512.57	10.7
B3LYPHF50		0.81	377.73	875.04	-508.14	10.84		0.76	377.78	875.05	-508.18	10.91		0.71	377.83	875.04	-508.13	10.92
BP86	$SiMe_3$	7.88	337.03	869.64	-537.75	5.14	$SiMe_3$	7.83	337.08	869.58	-537.65	5.15	$SiMe_3$	7.91	337	869.57	-537.74	5.17
SAOP		2.98	398.69	865.12	-472.75	6.33		2.85	398.82	864.99	-472.5	6.33		3.01	398.66	865.02	-472.71	6.35
B3LYPHF10		7.50	330.17	874.59	-550.09	5.66		7.54	330.13	874.59	-550.12	5.66		7.57	330.1	874.59	-550.19	5.7
B3LYPHF15		6.95	335.65	874.47	-544.6	5.78		7.00	335.6	874.46	-544.64	5.78		7.00	335.6	874.47	-544.7	5.83
B3LYPHF20		6.53	340.99	874.34	-539.22	5.88		6.60	340.92	874.33	-539.27	5.86		6.58	340.94	874.34	-539.32	5.93
B3LYPHF25		6.14	346.29	873.9	-533.54	5.93		6.20	346.23	873.9	-533.6	5.93		6.16	346.27	873.88	-533.6	5.99
B3LYPHF30		5.91	351.41	873.75	-528.33	5.99		5.99	351.33	873.76	-528.42	5.99		5.93	351.39	873.73	-528.4	6.06
B3LYPHF35		5.73	356.47	873.59	-523.18	6.06		5.83	356.37	873.61	-523.3	6.06		5.77	356.43	873.58	-523.26	6.12
B3LYPHF40		5.65	361.41	873.36	-518.07	6.12		5.76	361.3	873.33	-518.14	6.12		5.67	361.39	873.31	-518.1	6.18
B3LYPHF45		7.55	366.1	872.87	-512.94	6.18		7.72	365.93	872.86	-513.09	6.16		7.59	366.06	872.85	-513.03	6.24
B3LYPHF50		7.60	370.94	872.66	-507.97	6.25		7.81	370.73	872.65	-508.15	6.23		7.66	370.88	872.64	-508.06	6.3

Table S12. Computed σ_{11}, σ_{22}, σ_{33}, δ_{11}, δ_{22}, δ_{33}, κ (skew), and Ω (span) values for $La(THF)_2$ at the B3LYP-HF40 level.

Entry	σ_{11}	σ_{22}	σ_{33}	δ_{11}	δ_{22}	δ_{33}	κ	Ω
La-Si	412.04	447.21	550.33	-44.98	-80.15	-183.27	0.49	138.29
La-Si	410.68	448.14	549.97	-43.62	-81.08	-182.91	0.46	139.29
La-Si	411.83	447.03	550.50	-44.77	-79.97	-183.44	0.49	138.67
$SiMe_3$	348.53	359.03	371.85	18.53	8.03	-4.79	0.10	23.32
$SiMe_3$	349.46	359.08	371.54	17.60	7.98	-4.48	0.13	22.08
$SiMe_3$	347.47	359.38	371.63	19.59	7.68	-4.57	0.02	24.16
$SiMe_3$	355.42	365.01	389.09	11.64	2.05	-22.03	0.43	33.67
$SiMe_3$	354.00	365.1.	388.75	13.06	1.96	-21.69	0.36	34.75
$SiMe_3$	355.63	365.11	389.20	11.43	1.95	-22.14	0.44	33.57
$SiMe_3$	347.18	359.4.	371.54	19.88	7.66	-4.48	-0.01	24.36
$SiMe_3$	349.54	358.78	372.21	17.52	8.28	-5.15	0.18	22.67
$SiMe_3$	348.87	359.44	371.37	18.19	7.62	-4.31	0.06	22.5

Table S13. Computed σ_{11}, σ_{22}, σ_{33}, δ_{11}, δ_{22}, δ_{33}, κ (skew), and Ω (span) values for $La(THF)_1$ at the B3LYP-HF40 level.

Entry	σ_{11}	σ_{22}	σ_{33}	δ_{11}	δ_{22}	δ_{33}	κ	Ω
La-Si	379.6	409.7	515.2	-12.54	-42.64	-148.14	0.56	135.6
La-Si	375.6	411	517.5	-8.54	-43.94	-150.44	0.50	141.9
La-Si	386.9	413.2	517.6	-19.84	-46.14	-150.54	0.60	130.7
$SiMe_3$	342.2	363.5	381	24.86	3.56	-13.94	-0.10	38.8
$SiMe_3$	342	364.9	384.6	25.06	2.16	-17.54	-0.08	42.6
$SiMe_3$	340.7	363.6	384	26.36	3.46	-16.94	-0.06	43.3
$SiMe_3$	351.1	368.8	387.6	15.96	-1.74	-20.54	0.03	36.5
$SiMe_3$	352.7	368.3	387.3	14.36	-1.24	-20.24	0.10	34.6
$SiMe_3$	352.2	368.4	386.4	14.86	-1.34	-19.34	0.05	34.2
$SiMe_3$	343.9	363.4	379.7	23.16	3.66	-12.64	-0.09	35.8
$SiMe_3$	346.5	365.9	377.9	20.56	1.16	-10.84	-0.24	31.4
$SiMe_3$	343.2	364.1	380.5	23.86	2.96	-13.44	-0.12	37.3

Table S14. Computed σ_{11}, σ_{22}, σ_{33}, δ_{11}, δ_{22}, δ_{33}, κ (skew), and Ω (span) values for $La(THF)_0$ at the B3LYP-HF40 level.

Entry	σ_{11}	σ_{22}	σ_{33}	δ_{11}	δ_{22}	δ_{33}	κ	Ω
La-Si	358.81	360.28	514.44	8.25	6.78	-147.38	0.98	155.63
La-Si	357.83	359.39	514.63	9.23	7.67	-147.57	0.98	156.8
La-Si	358.98	359.41	514.07	8.08	7.65	-147.01	0.99	155.09
$SiMe_3$	344.45	363.73	376.18	22.61	3.33	-9.12	-0.22	31.73
$SiMe_3$	344.8	363.23	375.74	22.26	3.83	-8.68	-0.19	30.94
$SiMe_3$	344.36	364.13	375.76	22.7	2.93	-8.7	-0.26	31.4
$SiMe_3$	351.94	366.71	385.91	15.12	0.35	-18.85	0.13	33.97
$SiMe_3$	351.91	366.79	386.04	15.15	0.27	-18.98	0.13	34.13
$SiMe_3$	351.93	366.81	386.16	15.13	0.25	-19.1	0.13	34.23
$SiMe_3$	344.54	363.94	375.76	22.52	3.12	-8.7	-0.24	31.22
$SiMe_3$	344.29	363.91	375.71	22.77	3.15	-8.65	-0.25	31.42
$SiMe_3$	344.84	363.53	375.82	22.22	3.53	-8.76	-0.21	30.98

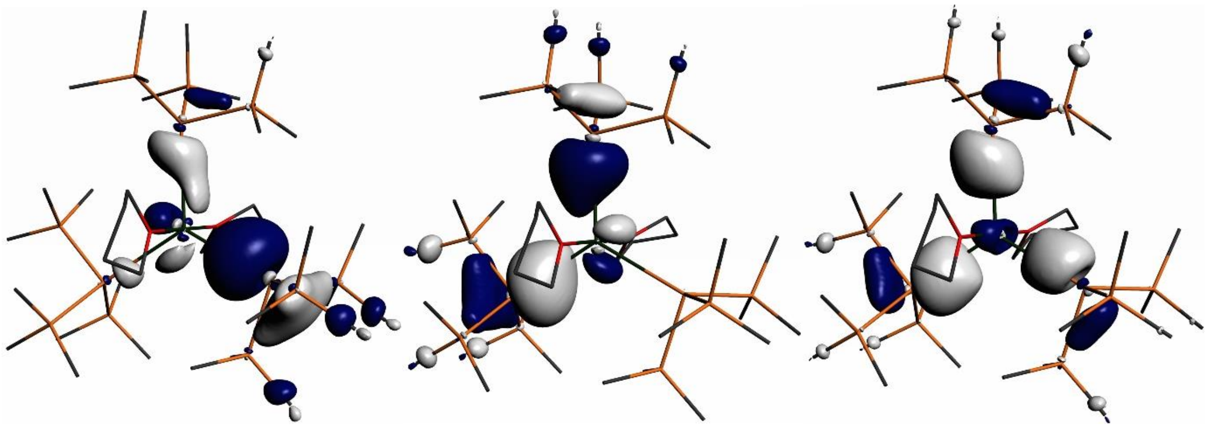

Figure S73. Selected Kohn-Sham Molecular Orbitals of **1-La**. Left to right: HOMO (274, −5.904 eV), HOMO−1 (273, −5.946 eV), HOMO−2 (272, −6.431 eV). Hydrogen atoms are omitted for clarity.

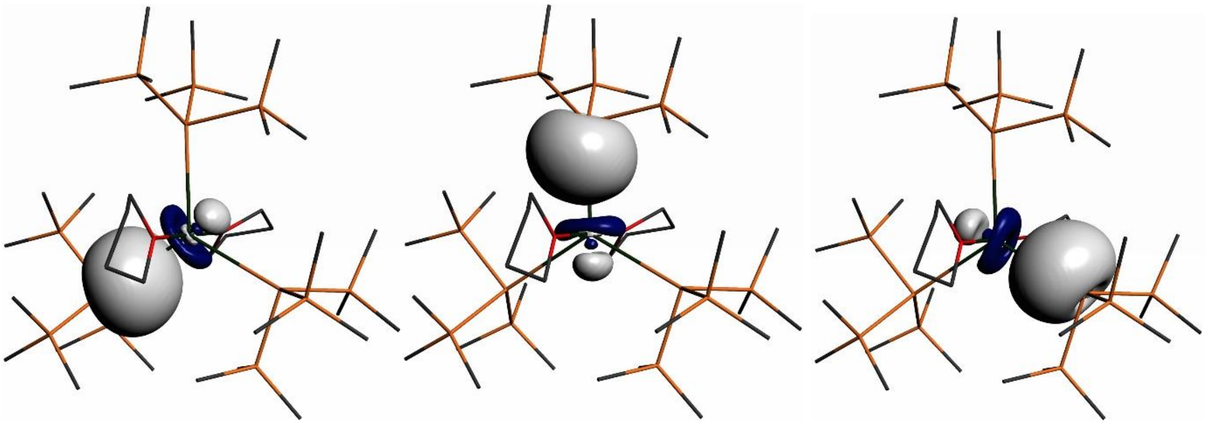

Figure S74. Natural Bond Orbital representations of the three La–Si bonds in **1-La**. Hydrogen atoms are omitted for clarity.

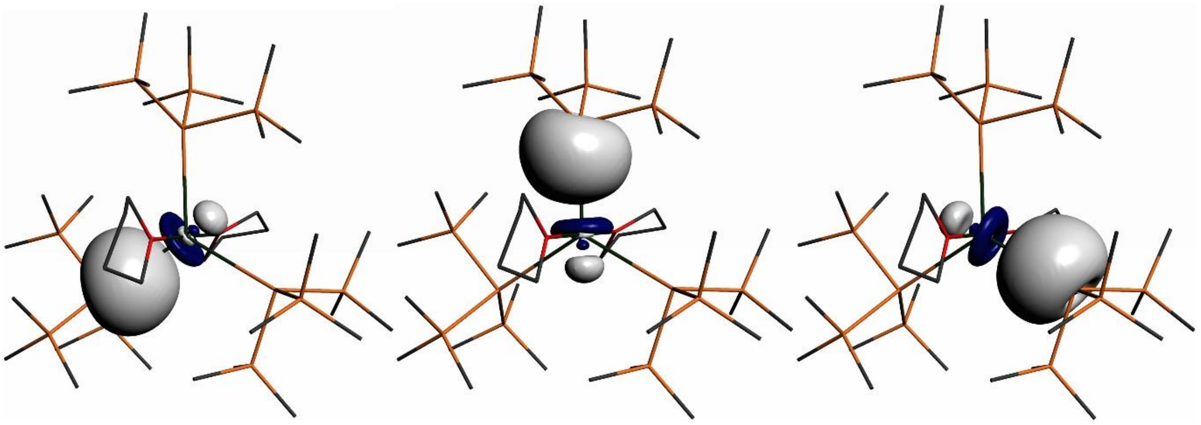

Figure S75. Natural Localized Molecular Orbital representations of the three La–Si bonds in **1-La**. Hydrogen atoms are omitted for clarity.

10. Complete Active Space Self-Consistent Field (CASSCF) Calculations

Table S15. Electronic structure of **1-Ce** as calculated with CASSCF-SO. CF wavefunction decomposed along the main magnetic axis of the ground doublet. The given angle is the deviation of g_3 between given doublet and ground doublet.

Energy (cm^{-1})	g_1	g_2	g_3	Angle (°)	Wavefunction [a]	$<J_z>$
0	2.3775	0.9462	0.7148	-	$97\%\|\pm 3/2\rangle + 2\%\|\mp 1/2\rangle$	± 1.450
196.49	4.3203	0.1038	0.0230	0.86	$99.7\%\|\pm 5/2\rangle$	± 2.494
528.01	3.4455	1.5225	0.5112	89.59	$97\%\|\pm 1/2\rangle + 2\%\|\mp 3/2\rangle$	± 0.455

a = Contributions > 1%.

Table S16. Electronic structure of **1-Pr** as calculated with CASSCF-SO. CF wavefunction decomposed along the main magnetic axis of the ground pseudo-doublet.

Energy (cm^{-1})	Wavefunction [a]
0	$49.4\%\|+4\rangle + 49.4\%\|-4\rangle$
6.05	$49.9\%\|+4\rangle + 49.9\%\|-4\rangle$
128.27	$95\%\|0\rangle + 2\%\|+2\rangle + 2\%-2\rangle$
231.85	$42\%\|+1\rangle + 42\%\|-1\rangle + 6\%\|+3\rangle + 6\%\|-3\rangle + 1\%\|0\rangle$
248.97	$40\%\|+1\rangle + 40\%\|-1\rangle + 8\%\|+3\rangle + 8\%\|-3\rangle + 2\%\|0\rangle$
373.63	$40\%\|+3\rangle + 40\%\|-3\rangle + 9\%\|+1\rangle + 9\%\|-1\rangle$
416.08	$42\%\|+3\rangle + 42\%\|-3\rangle + 5\%\|+1\rangle + 5\%\|-1\rangle + 4\%\|+2\rangle + 4\%\|-2\rangle$
486.95	$45\%\|+2\rangle + 45\%\|-2\rangle + 3\%\|+3\rangle + 3\%\|-3\rangle + 1\%\|+1\rangle + 1\%\|-1\rangle$
487.38	$48\%\|+2\rangle + 48\%\|-2\rangle$

a = Contributions > 1%.

Table S17. Electronic structure of **1-Nd** as calculated with CASSCF-SO. CF wavefunction decomposed along the main magnetic axis of the ground doublet. The given angle is the deviation of g_3 between given doublet and ground doublet.

**Energy (cm^{-1})**	g_1	g_2	g_3	**Angle (°)**	**Wavefunction** [a]	**<J$_z$>**
0	6.0319	0.4034	0.3246	-	$90\%\|\pm 9/2\rangle$ + $7\%\|\pm 5/2\rangle$ + $3\%\|\mp 3/2\rangle$	$\pm$4.193
135.86	3.5779	1.5081	1.1453	89.73	$82\%\|\pm 3/2\rangle$ + $13\%\|\mp 1/2\rangle$ + $3\%\|\mp 5/2\rangle$ + $1\%\|\mp 9/2\rangle$	$\pm$1.034
171.97	3.2025	1.7739	0.7926	1.67	$83\%\|\pm 5/2\rangle$ + $8\%\|\pm 9/2\rangle$ + $6\%\|\mp 7/2\rangle$ + $2\%\|\pm 1/2\rangle$ + $1\%\|\mp 3/2\rangle$	$\pm$2.214
207.65	5.7411	0.7606	0.3121	89.99	$85\%\|\pm 1/2\rangle$ + $14\%\|\mp 3/2\rangle$	$\pm$0.243
318.93	4.6160	1.5650	1.2747	0.16	$94\%\pm 7/2\rangle$ + $6\%\|\mp 5/2\rangle$	$\pm$3.118

a = Contributions > 1%.

Table S18. Electronic structure of **1-U** as calculated with CASSCF-SO. CF wavefunction decomposed along the main magnetic axis of the ground doublet. The given angle is the deviation of g_3 between given doublet and ground doublet.

**Energy (cm^{-1})**	g_1	g_2	g_3	**Angle (°)**	**Wavefunction** [a]	**<J$_z$>**
0	6.0970	0.5480	0.0448	-	$89\%\|\pm 9/2\rangle$ + $11\%\|\pm 5/2\rangle$	$\pm$4.274
359.01	2.2695	1.7912	1.7517	89.78	$91\%\|\pm 3/2\rangle$ + $8\%\|\mp 1/2\rangle$	$\pm$1.320
435.77	3.9837	0.6393	0.3542	0.07	$88\%\|\pm 5/2\rangle$ + $11\%\|\pm 9/2\rangle$	$\pm$2.683
576.86	5.3883	1.2991	0.3975	88.99	$92\%\|\pm 1/2\rangle$ + $8\%\|\mp 3/2\rangle$	$\pm$0.353
773.76	5.3654	0.1846	0.1570	0.01	$99.7\%\|\pm 7/2\rangle$	$\pm$3.490

a = Contributions > 1%.

11. pNMR Calculations

Table S19. Selected bond distances (Å) and angles (°) for **1-Ce**.

	**M–Si/ Å**	**M–O/ Å**	**Si–M–Si / °**	**O–M–Si / °**	**O–M–O / °**	**M(Si)$_3$**
**1-Ce XRD**	3.2	2.4	120.0	92.5	173.8	Trigonal planar
**Ce(THF)$_2$ opt**	3.2	2.5	120.0	89.9	175.0	Trigonal planar
**Ce(THF)$_1$ opt**	3.1	2.4	119.7	90.3		Pyramidal
**Ce(THF)$_0$ opt**	3.1		120.0			Pyramidal

Table S20. Selected bond distances (Å) and angles (°) for **1-Pr**.

	**M–Si/ Å**	**M–O/ Å**	**Si–M–Si / °**	**O–M–Si / °**	**O–M–O / °**	**M(Si)$_3$**
**1-Pr XRD**	3.2	2.4, 2.5	120	87.6, 91.3	180.0	Trigonal planar
**Pr(THF)$_2$ opt**	3.2	2.5	119.8	89.9	175.7	Trigonal planar

**Pr(THF)$_1$ opt**	3.1	2.4	120.0	90.9		Pyramidal
**Pr(THF)$_0$ opt**	3		113.9			Pyramidal

Table S21. Selected bond distances (Å) and angles (°) for **1-Nd**.

	**M–Si/ Å**	**M–O/ Å**	**Si–M–Si / °**	**O–M–Si / °**	**O–M–O / °**	**M(Si)$_3$**
**1-Nd XRD**	3.1	2.4	120	90.0	180.0	Trigonal planar
**Nd(THF)$_2$ opt**	3.2	2.5	119.9	89.9	171.0	Trigonal planar
**Nd(THF)$_1$ opt**	3.1	2.5	119.7	93.6		Pyramidal

**Nd(THF)$_0$ opt**	3.0		114.9			Pyramidal

Table S22. Principal *g*-values for ground Kramers doublets in **1-Ce**.

	g_1	g_2	g_3
**1-Ce XRD**	0.7142	0.9468	2.3775
**Ce(THF)$_2$ opt**	0.0327	0.5262	2.8004
**Ce(THF)$_1$ opt**	0.3322	0.5645	3.3719
**Ce(THF)$_0$ opt**	0.8512	2.4659	2.4777

Table S23. Principal *g*-values for ground Kramers doublets in **1-Nd**.

	g_1	g_2	g_3
**1-Nd XRD**	0.3185	0.3935	5.9891
**$Nd(THF)_2$ opt**	0.4068	0.5384	5.4884
**$Nd(THF)_1$ opt**	0.5436	1.4394	5.3569
**$Nd(THF)_0$ opt**	0.2711	3.2905	3.2996

Table S24. Calculated average ^{1}H and ^{29}Si $\delta_{\mathrm{PCS}}^{\mathrm{para}}$, $\delta_{\mathrm{vdH}}^{\mathrm{para}}$ and experimental $\delta_{\mathrm{exp}}^{\mathrm{para}}$ for **1-Ce**. Using "in-plane"/"out-of-plane" for group 1/group 2 averaging.

**Model**	**^{1}H Group 1**			**^{1}H Group 2**			**^{29}Si Group 1**			**^{29}Si Group 2**			**$^{29}Si_M$**		
	$\delta_{\mathrm{exp}}^{\mathrm{para}}$	$\delta_{\mathrm{vdH-S}}^{\mathrm{para}}$	$\delta_{\mathrm{PCS}}^{\mathrm{para}}$	$\delta_{\mathrm{exp}}^{\mathrm{para}}$	$\delta_{\mathrm{vdH-S}}^{\mathrm{para}}$	$\delta_{\mathrm{PCS}}^{\mathrm{para}}$	$\delta_{\mathrm{exp}}^{\mathrm{para}}$	$\delta_{\mathrm{vdH-S}}^{\mathrm{para}}$	$\delta_{\mathrm{PCS}}^{\mathrm{para}}$	$\delta_{\mathrm{exp}}^{\mathrm{para}}$	$\delta_{\mathrm{vdH-S}}^{\mathrm{para}}$	$\delta_{\mathrm{PCS}}^{\mathrm{para}}$	$\delta_{\mathrm{exp}}^{\mathrm{para}}$	$\delta_{\mathrm{vdH-S}}^{\mathrm{para}}$	$\delta_{\mathrm{PCS}}^{\mathrm{para}}$
**1-Ce XRD**	1.3	-2.6	-2.6	-1.8	-0.4	-0.4	1.7	-7.6	-5.7	-1.1	-3.4	-2.3	2.9	-21.5	-13.9
**$Ce(THF)_2$ opt**		-2.2	-2.2		-0.6	-0.6		-7.2	-5.0		-3.6	-2.3		-20.9	-11.8
**$Ce(THF)_1$ opt**		4.5	4.5		-0.8	-0.9		10.1	11.2		2.9	4.8		20.1	33.7
**$Ce(THF)_0$ opt**		4.6	4.7		-6.5	-6.3		9.5	11.1		1.9	8.4		31.5	46.6

Table S25. Calculated average ^{1}H and [29]Si $\delta^{\mathrm{para}}_{\mathrm{PCS}}$, $\delta^{\mathrm{para}}_{\mathrm{vdH}}$ and experimental $\delta^{\mathrm{para}}_{\mathrm{exp}}$ for **1-Pr**. Using "in-plane"/"out-of-plane" for group 1/group 2 averaging.

Model	^{1}H Group 1			^{1}H Group 2			^{29}Si Group 1			^{29}Si Group 2			^{29}Si$_{M}$		
	$\delta^{\mathrm{para}}_{\mathrm{exp}}$	$\delta^{\mathrm{para}}_{\mathrm{vdH-S}}$	$\delta^{\mathrm{para}}_{\mathrm{PCS}}$	$\delta^{\mathrm{para}}_{\mathrm{exp}}$	$\delta^{\mathrm{para}}_{\mathrm{vdH-S}}$	$\delta^{\mathrm{para}}_{\mathrm{PCS}}$	$\delta^{\mathrm{para}}_{\mathrm{exp}}$	$\delta^{\mathrm{para}}_{\mathrm{vdH-S}}$	$\delta^{\mathrm{para}}_{\mathrm{PCS}}$	$\delta^{\mathrm{para}}_{\mathrm{exp}}$	$\delta^{\mathrm{para}}_{\mathrm{vdH-S}}$	$\delta^{\mathrm{para}}_{\mathrm{PCS}}$	$\delta^{\mathrm{para}}_{\mathrm{exp}}$	$\delta^{\mathrm{para}}_{\mathrm{vdH-S}}$	$\delta^{\mathrm{para}}_{\mathrm{PCS}}$
**1-Pr XRD**	7.9	-14.6	-14.5	-8.1	-1.9	-1.8	10.2	-40.9	-33.1	-	-14.9	-11.9	17.3	-113.7	-73.8
**$Pr(THF)_2$ opt**		-4.4	-4.3		-0.5	-0.4		-15.0	-8.8		-5.7	-2.7		-55.9	-14.5
**$Pr(THF)_1$ opt**		10.9	11.1		-8.0	-8.1		24.0	29.4		2.5	8.7		46.7	89.0
**$Pr(THF)_0$ opt**		7.3	7.9		-6.2	-6.3		16.0	23.2		7.0	17.1		36.0	77.3

Table S26. Calculated average ^{1}H and [29]Si $\delta^{\mathrm{para}}_{\mathrm{PCS}}$, $\delta^{\mathrm{para}}_{\mathrm{vdH}}$ and experimental $\delta^{\mathrm{para}}_{\mathrm{exp}}$ for **1-Nd**. Using "in-plane"/"out-of-plane" for group 1/group 2 averaging.

Model	^{1}H Group 1			^{1}H Group 2			^{29}Si Group 1			^{29}Si Group 2			^{29}Si$_{M}$		
	$\delta^{\mathrm{para}}_{\mathrm{exp}}$	$\delta^{\mathrm{para}}_{\mathrm{vdH-S}}$	$\delta^{\mathrm{para}}_{\mathrm{PCS}}$	$\delta^{\mathrm{para}}_{\mathrm{exp}}$	$\delta^{\mathrm{para}}_{\mathrm{vdH-S}}$	$\delta^{\mathrm{para}}_{\mathrm{PCS}}$	$\delta^{\mathrm{para}}_{\mathrm{exp}}$	$\delta^{\mathrm{para}}_{\mathrm{vdH-S}}$	$\delta^{\mathrm{para}}_{\mathrm{PCS}}$	$\delta^{\mathrm{para}}_{\mathrm{exp}}$	$\delta^{\mathrm{para}}_{\mathrm{vdH-S}}$	$\delta^{\mathrm{para}}_{\mathrm{PCS}}$	$\delta^{\mathrm{para}}_{\mathrm{exp}}$	$\delta^{\mathrm{para}}_{\mathrm{vdH-S}}$	$\delta^{\mathrm{para}}_{\mathrm{PCS}}$
**1-Nd XRD**	4.9	-6.4	-6.4	-5.0	-0.6	-0.5	6.3	-22.3	-15.3	-	-9.5	-6.1	10.7	-94.1	-38.6
**$Nd(THF)_2$ opt**		-2.3	-2.3		-0.4	-0.4		-11.7	-5.4		-5.7	-2.3		-72.1	-13.2
**$Nd(THF)_1$ opt**		5.7	5.9		-4.2	-4.2		8.7	16.8		-1.8	4.4		-9.6	49.8
**$Nd(THF)_0$ opt**		5.8	5.9		-5.9	-5.3		8.0	12.9		-9.9	10.7		-15.3	55.3

Table S27. Calculated average ^{1}H and [29]Si $\delta_{\mathrm{PCS}}^{\mathrm{para}}$, $\delta_{\mathrm{vdH}}^{\mathrm{para}}$ and experimental $\delta_{\mathrm{exp}}^{\mathrm{para}}$ for **1-Ce**. Using second averaging method: group 1 is farthest from the $M(Si_M)_3$ plane.

Model	^{1}H Group 1			^{1}H Group 2			^{29}Si Group 1			^{29}Si Group 2		
	$\delta_{\mathrm{exp}}^{\mathrm{para}}$	$\delta_{\mathrm{vdH-S}}^{\mathrm{para}}$	$\delta_{\mathrm{PCS}}^{\mathrm{para}}$	$\delta_{\mathrm{exp}}^{\mathrm{para}}$	$\delta_{\mathrm{vdH-S}}^{\mathrm{para}}$	$\delta_{\mathrm{PCS}}^{\mathrm{para}}$	$\delta_{\mathrm{exp}}^{\mathrm{para}}$	$\delta_{\mathrm{vdH-S}}^{\mathrm{para}}$	$\delta_{\mathrm{PCS}}^{\mathrm{para}}$	$\delta_{\mathrm{exp}}^{\mathrm{para}}$	$\delta_{\mathrm{vdH-S}}^{\mathrm{para}}$	$\delta_{\mathrm{PCS}}^{\mathrm{para}}$
**$Ce(THF)_1$ opt**	1.3	-2.5	-2.9	-1.8	2.7	2.8	1.7	2.6	4.4	-1.1	6.7	8.2
**$Ce(THF)_0$ opt**		-6.1	-5.9		-1.1	-1.1		8.1	17.9		2.6	5.0

Table S28. Calculated average ^{1}H and [29]Si $\delta_{\mathrm{PCS}}^{\mathrm{para}}$, $\delta_{\mathrm{vdH}}^{\mathrm{para}}$ and experimental $\delta_{\mathrm{exp}}^{\mathrm{para}}$ for **1-Pr**. Using second averaging method: group 1 is farthest from the $M(Si_M)_3$ plane.

Model	^{1}H Group 1			^{1}H Group 2			^{29}Si Group 1			^{29}Si Group 2		
	$\delta_{\mathrm{exp}}^{\mathrm{para}}$	$\delta_{\mathrm{vdH-S}}^{\mathrm{para}}$	$\delta_{\mathrm{PCS}}^{\mathrm{para}}$	$\delta_{\mathrm{exp}}^{\mathrm{para}}$	$\delta_{\mathrm{vdH-S}}^{\mathrm{para}}$	$\delta_{\mathrm{PCS}}^{\mathrm{para}}$	$\delta_{\mathrm{exp}}^{\mathrm{para}}$	$\delta_{\mathrm{vdH-S}}^{\mathrm{para}}$	$\delta_{\mathrm{PCS}}^{\mathrm{para}}$	$\delta_{\mathrm{exp}}^{\mathrm{para}}$	$\delta_{\mathrm{vdH-S}}^{\mathrm{para}}$	$\delta_{\mathrm{PCS}}^{\mathrm{para}}$
**$Pr(THF)_1$ opt**	7.9	-19.8	-20.2	-8.1	7.3	7.5	10.2	-6.1	2.4	-	17.6	22.2
**$Pr(THF)_0$ opt**		3.2	3.6		-4.2	-4.2		6.8	10.9		11.6	23.2

Table S29. Calculated average ^{1}H and [29]Si $\delta_{\mathrm{PCS}}^{\mathrm{para}}$, $\delta_{\mathrm{vdH}}^{\mathrm{para}}$ and experimental $\delta_{\mathrm{exp}}^{\mathrm{para}}$ for **1-Nd**. Using second averaging method: group 1 is farthest from the $M(Si_M)_3$ plane.

Model	^{1}H Group 1			^{1}H Group 2			^{29}Si Group 1			^{29}Si Group 2		
	$\delta_{\mathrm{exp}}^{\mathrm{para}}$	$\delta_{\mathrm{vdH-S}}^{\mathrm{para}}$	$\delta_{\mathrm{PCS}}^{\mathrm{para}}$	$\delta_{\mathrm{exp}}^{\mathrm{para}}$	$\delta_{\mathrm{vdH-S}}^{\mathrm{para}}$	$\delta_{\mathrm{PCS}}^{\mathrm{para}}$	$\delta_{\mathrm{exp}}^{\mathrm{para}}$	$\delta_{\mathrm{vdH-S}}^{\mathrm{para}}$	$\delta_{\mathrm{PCS}}^{\mathrm{para}}$	$\delta_{\mathrm{exp}}^{\mathrm{para}}$	$\delta_{\mathrm{vdH-S}}^{\mathrm{para}}$	$\delta_{\mathrm{PCS}}^{\mathrm{para}}$
**$Nd(THF)_1$ opt**	4.9	-11.2	-11.5	-5.0	4.3	4.5	6.3	-8.2	-0.4	-	6.7	13.0

**Nd(THF)₀ opt**		-5.9	-5.2		0.0	0.2		-9.9	-1.4		-0.9	17.9

Table S30. Calculated average ^{1}H and [29]Si $\delta_{\mathrm{PCS}}^{\mathrm{para}}$, $\delta_{\mathrm{vdH}}^{\mathrm{para}}$ and experimental $\delta_{\mathrm{exp}}^{\mathrm{para}}$ for **1-Ce**. Using third averaging method: group 2 is farthest and closest to $M(Si_M)_3$ plane.

**Model**	**^{1}H Group 1**			**^{1}H Group 2**			**^{29}Si Group 1**			**^{29}Si Group 2**		
	$\boldsymbol{\delta_{\mathrm{exp}}^{\mathrm{para}}}$	$\boldsymbol{\delta_{\mathrm{vdH-S}}^{\mathrm{para}}}$	$\boldsymbol{\delta_{\mathrm{PCS}}^{\mathrm{para}}}$	$\boldsymbol{\delta_{\mathrm{exp}}^{\mathrm{para}}}$	$\boldsymbol{\delta_{\mathrm{vdH-S}}^{\mathrm{para}}}$	$\boldsymbol{\delta_{\mathrm{PCS}}^{\mathrm{para}}}$	$\boldsymbol{\delta_{\mathrm{exp}}^{\mathrm{para}}}$	$\boldsymbol{\delta_{\mathrm{vdH-S}}^{\mathrm{para}}}$	$\boldsymbol{\delta_{\mathrm{PCS}}^{\mathrm{para}}}$	$\boldsymbol{\delta_{\mathrm{exp}}^{\mathrm{para}}}$	$\boldsymbol{\delta_{\mathrm{vdH-S}}^{\mathrm{para}}}$	$\boldsymbol{\delta_{\mathrm{PCS}}^{\mathrm{para}}}$
**Ce(THF)₁ opt**	1.3	0.9	1.1	-1.8	1.0	0.8	1.7	3.3	5.2	-1.1	6.4	7.8
**Ce(THF)₀ opt**		-6.9	-6.8		-0.7	-0.6		-4.2	-1.0		8.8	14.5

Table S31. Calculated average ^{1}H and [29]Si $\delta_{\mathrm{PCS}}^{\mathrm{para}}$, $\delta_{\mathrm{vdH}}^{\mathrm{para}}$ and experimental $\delta_{\mathrm{exp}}^{\mathrm{para}}$ for **1-Pr**. Using third averaging method: group 2 is farthest and closest to $M(Si_M)_3$ plane.

**Model**	**^{1}H Group 1**			**^{1}H Group 2**			**^{29}Si Group 1**			**^{29}Si Group 2**		
	$\boldsymbol{\delta_{\mathrm{exp}}^{\mathrm{para}}}$	$\boldsymbol{\delta_{\mathrm{vdH-S}}^{\mathrm{para}}}$	$\boldsymbol{\delta_{\mathrm{PCS}}^{\mathrm{para}}}$	$\boldsymbol{\delta_{\mathrm{exp}}^{\mathrm{para}}}$	$\boldsymbol{\delta_{\mathrm{vdH-S}}^{\mathrm{para}}}$	$\boldsymbol{\delta_{\mathrm{PCS}}^{\mathrm{para}}}$	$\boldsymbol{\delta_{\mathrm{exp}}^{\mathrm{para}}}$	$\boldsymbol{\delta_{\mathrm{vdH-S}}^{\mathrm{para}}}$	$\boldsymbol{\delta_{\mathrm{PCS}}^{\mathrm{para}}}$	$\boldsymbol{\delta_{\mathrm{exp}}^{\mathrm{para}}}$	$\boldsymbol{\delta_{\mathrm{vdH-S}}^{\mathrm{para}}}$	$\boldsymbol{\delta_{\mathrm{PCS}}^{\mathrm{para}}}$
**Pr(THF)₁ opt**	7.9	3.8	4.0	-8.1	-4.5	-4.6	10.2	11.1	15.0	-	9.0	15.9
**Pr(THF)₀ opt**		-15.6	-16.2		5.3	5.7		7.2	23.3		11.4	17.0

Table S32. Calculated average ^{1}H and [29]Si $\delta_{\mathrm{PCS}}^{\mathrm{para}}$, $\delta_{\mathrm{vdH}}^{\mathrm{para}}$ and experimental $\delta_{\mathrm{exp}}^{\mathrm{para}}$ for **1-Nd**. Using third averaging method: group 2 is farthest and closest to $M(Si_M)_3$ plane.

**Model**	**^{1}H Group 1**			**^{1}H Group 2**			**^{29}Si Group 1**			**^{29}Si Group 2**		
	$\boldsymbol{\delta_{\mathrm{exp}}^{\mathrm{para}}}$	$\boldsymbol{\delta_{\mathrm{vdH-S}}^{\mathrm{para}}}$	$\boldsymbol{\delta_{\mathrm{PCS}}^{\mathrm{para}}}$	$\boldsymbol{\delta_{\mathrm{exp}}^{\mathrm{para}}}$	$\boldsymbol{\delta_{\mathrm{vdH-S}}^{\mathrm{para}}}$	$\boldsymbol{\delta_{\mathrm{PCS}}^{\mathrm{para}}}$	$\boldsymbol{\delta_{\mathrm{exp}}^{\mathrm{para}}}$	$\boldsymbol{\delta_{\mathrm{vdH-S}}^{\mathrm{para}}}$	$\boldsymbol{\delta_{\mathrm{PCS}}^{\mathrm{para}}}$	$\boldsymbol{\delta_{\mathrm{exp}}^{\mathrm{para}}}$	$\boldsymbol{\delta_{\mathrm{vdH-S}}^{\mathrm{para}}}$	$\boldsymbol{\delta_{\mathrm{PCS}}^{\mathrm{para}}}$
**Nd(THF)₁ opt**	4.9	2.9	3.0	-5.0	-2.7	-2.8	6.3	4.7	9.2	-	0.3	8.2

**$Nd(THF)_0$ opt**		-5.8	-5.4		-0.1	0.3		-9.8	22.9		-0.9	5.8

12. References

1. *WSolids1 ver. 1.21.7*, Eichele, K., Universität Tübingen, 2021.
